# Nine New Gingerols from the Rhizoma of *Zingiber officinale* and Their Cytotoxic Activities

**DOI:** 10.3390/molecules23020315

**Published:** 2018-02-02

**Authors:** Zezhi Li, Yanzhi Wang, MeiLing Gao, Wanhua Cui, Mengnan Zeng, Yongxian Cheng, Juan Li

**Affiliations:** 1 School of Pharmacy, Henan University of Chinese Medicine, Zhengzhou 450046, China; lzzdyq1992@163.com (Z.L.); gaoxiaomei6266@126.com (M.G.); qingyixin@163.com (W.C.); 13598851831@139.com (M.Z.); yxcheng@szu.edu.cn (Y.C.); jli_henantcm2017@163.com (J.L.); 2 Collaborative Innovation Center for Respiratory Disease Diagnosis, Treatment and New Drug Research and Development of Henan Province, Henan University of Chinese Medicine, Zhengzhou 450046, China; 3 Guangdong Key Laboratory for Genome Stability & Disease Prevention, School of Pharmaceutical Sciences, School of Medicine, Shenzhen University Health Science Center, Shenzhen 518060, China

**Keywords:** *Zingiber officinale*, gingerols, cytotoxic activity

## Abstract

Nine new gingerols, including three 6-oxo-shogaol derivatives [(*Z*)-6-oxo-[6]-shogaol (**1**), (*Z*)-6-oxo-[8]-shogaol (**2**), (*Z*)-6-oxo-[10]-shogaol (**3**)], one 6-oxoparadol derivative [6-oxo-[6]-paradol (**4**)], one isoshogaol derivative [(*E*)-[4]-isoshogaol (**5**)], and four paradoldiene derivatives [(4*E*,6*Z*)-[4]-paradoldiene (**8**), (4*E*,6*E*)-[6]-paradoldiene (**9**), (4*E*,6*E*)-[8]-paradoldiene (**10**), (4*E*,6*Z*)-[8]-paradoldiene (**11**)], together with eight known analogues, were isolated from the rhizoma of *Zingiber officinale*. Their structures were elucidated on the basis of spectroscopic data. It was noted that the isolation of 6-oxo-shogaol derivatives represents the first report of gingerols containing one 1,4-enedione motif. Their structures were elucidated on the basis of spectroscopic and HRESIMS data. All the new compounds were evaluated for their cytotoxic activities against human cancer cells (MCF-7, HepG-2, KYSE-150).

## 1. Introduction

**Figure 1:**
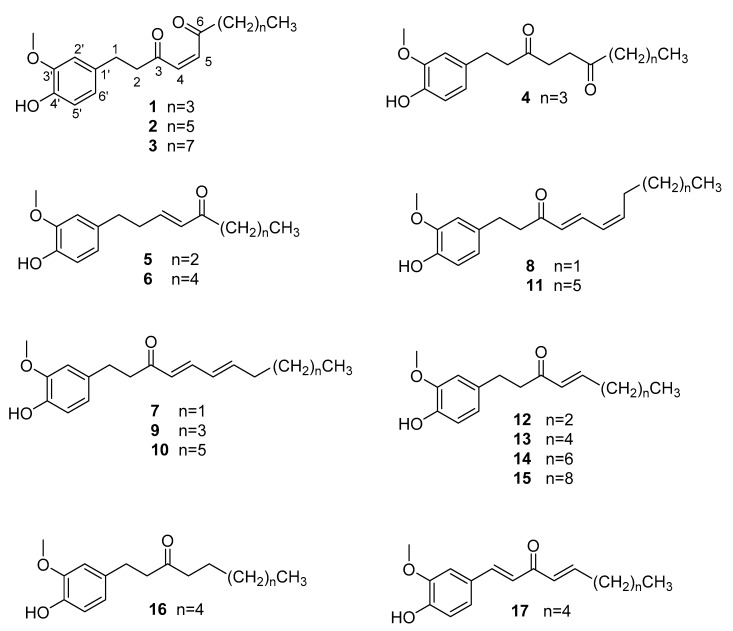
Structures of compounds **1**–**17**.

Ginger, also known as white ginger, is the dry rhizoma of *Zingiber officinale*, which has been a popular spice world-wide. As a Chinese medicine, it has been used in the treatment of nausea and vomiting, coughs, cold and so forth, for more than 2000 years [1,2,3]. In the traditional Chinese medicine theory system, *Z. officinale* is a warm medicine which can warm the spleen and stomach to dispel cold and warm the lungs, to reduce or eliminate dampness and phlegm (Huiyang Tongmai). Pharmacological studies on *Z. officinale* showed its effects on oxidative stress [4,5], tumor [6,7], degenerative diseases [8], vomiting [9], the cardiovascular system [10], and indigestion [11,12]. Chemical investigations have revealed the presence of volatile oils, gingerols, and diarylheptanoids, responsible for its pungency and other pharmacological properties [13]. Among the various activities of gingerols, numerous documents have reported anti-tumor activities, so we were interested in finding new types of gingerols with anti-tumor activity. Our efforts resulted in the isolation of nine new gingerols and eight known analogues (Figure 1), whose cytotoxic activities against human cancer cells (MCF-7, HepG-2, KYSE-150) were subsequently evaluated. 

## 2. Results and Discussion

### Structure Elucidation of the Compounds

The EtOAc extract of *Z. officinale* rhizoma was submitted to a combination of column chromatography to produce compounds **1**–**17** (Figure 1). 

**Figure 2:**
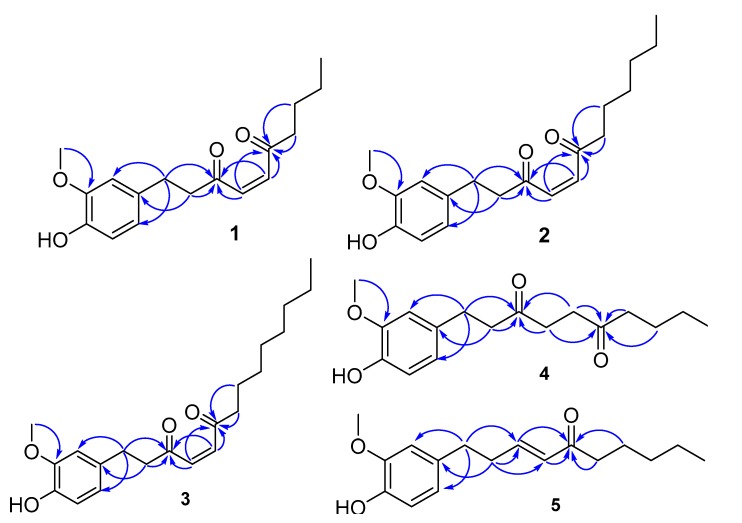
Key HMBC correlations of compounds **1**–**5**.

Compound **2** was isolated as yellow oil and is demonstrated by a molecular formula of C_19_H_26_O_4_, based on the HRESIMS ion peak at *m*/*z* 341.1727 [M + Na]^+^ (calcd. for 341.1729).The ^1^H-NMR (Table 1) spectrum of **2** shows the typical pattern of a coupling group of 1,3,4-trisubstituted benzene rings at *δ*_H_ 6.81 (1H, d, *J* = 8.0 Hz, H-5′), 6.67 (1H, d, *J* = 1.5 Hz, H-2′), 6.66 (1H, dd, *J* = 8.0, 1.5 Hz, H-6′), two olefinic protons [*δ*_H_ 6.83 (2H, s, H-4, H-5)], a methoxy group [*δ*_H_ 3.85 (3H, s, 3′-OCH_3_)], seven methylene groups [*δ*_H_ 2.86 (2H, m, H-1), 2.94 (2H, m, H-2), 2.59 (2H, t, *J* = 7.4 Hz, H-7), 1.60 (2H, m, H-8), 1.28 (6H, m, H-9, H-10, H-11)] and a methyl group [*δ*_H_ 0.85 (3H, t, *J* = 6.4 Hz, H-12)]. In the ^13^C-NMR spectrum of **2**, 19 carbon signals were observed, which included two carbonyls [*δ*_C_ 200.7 (C-3) and 199.8 (C-6)], six aromatic carbons [*δ*_C_ 146.4 (C-3′), 144.0 (C-4′), 132.4 (C-1′), 120.8 (C-6), 114.4 (C-5), 111.0 (C-2)], two olefinic carbons [*δ*_C_ 136.4 (C-4) and 136.1 (C-5)], and eight aliphatic carbons [*δ*_C_ 43.5 (C-2), 41.7 (C-7), 31.5 (C-10), 29.3 (C-1), 28.8 (C-9), 23.7 (C-8), 22.4 (C-11), 14.0 (C-12)]. The planar structure of **2** was further demonstrated by analyses of 2D NMR spectra. The HSQC showed that there is a correlation from *δ*_H_ 6.83 (2H, s, H-4, H-5) to two olefinic carbons (*δ*_C_ 136.4, C-4 and 136.1, C-5); while the HMBC presented a correlation from *δ*_H_ 6.83 (2H, s, H-4, H-5) to two carbonyl carbons [*δ*_C_ 200.7 (C-3) and 199.8 (C-6)], as well as a correlation from the methylene protons (H-1, H-2, H-7 and H-8) to two carbonyl carbons, but lacking the correlations from H-4 and H-5 to C-2 and C-7 or from H-2 and H-7 to C-4 and C-5 (Figure 2). It was found that there are two olefinic carbons between two carbonyl carbons. In addition, the methoxy group at *δ*_H_ 3.85 (3′-OCH_3_) was located at C-3′ by the HMBC correlations from 3′-OCH_3_ to C-3′, the substituent at C-4′was identified as a hydroxy group because of its ^13^C-NMR chemical shift, and the side chain was located at C-1′ by HMBC correlations from H-1 to C-1′, C-2′ and C-3′ and from H-2 to C-1′.

Finally, our focus was on determining the *cis*/*trans* configuration of double bonds. Due to the molecular symmetry, the magnetically equivalent vinylic protons of **2** behave as a singlet peak. Thus, it was impossible to directly determine the *E*/*Z*-configuration of the double bonds by the size of the vicinal coupling constants. We tested a variety of methodologies to solve this problem, including trying to cultivate single crystals, changing different deuterated reagents, and looking for more evidence on 2D NMR spectra, but none of them succeeded. After reviewing relevant literature [14], we found that olefinic protons split obviously when the compounds containing 1,4-enedione are in NMR spectra with deuterated benzene as a solvent. Subsequently, we changed the solvent to deuterated benzene, and we found that the singlet peak of olefinic protons became a quadruplet of AB system. The AB system is an advanced coupling and the coupling constant cannot be calculated directly. We calculated the actual chemical shift of the quadruplet, and the coupling constant was calculated to be 10.4 Hz by the chemical shift. Therefore, the two olefinic protons were *cis*-oriented, and the structure of **2** was determined to be (*Z*)-6-oxo-[8]-shogaol.

Compound **3** was obtained as yellow oil and shows a [M + Na]^+^ ion at *m*/*z* 369.2042 in the HRESIMS, consistent with a molecular formula of C_21_H_30_O_4_ (calcd. for 369.2042). Compound **1** was obtained as yellow oil, with the molecular formula C_17_H_22_O_4_, as determined by the HREIMS at *m*/*z* 313.1414 for the [M + Na]^+^ (calcd. for 313.1416). The ^1^H- and ^13^C-NMR data (Table 1) of **3** and **1** are similar to those of **2**, suggesting that they have the same carbon skeleton. The significant differences in these three compounds are the number of carbons in the aliphatic region of the ^13^C-NMR and the ^1^H-NMR spectra. Compound **3** has two more methylene groups in its side-chain than **2**, while **1** has two fewer methylene groups in side-chain than **2**. Similarly, the double bond moieties of **3** and **1** both behaved as a typical AB system in their ^1^H-NMR spectrum when deuterated benzene was used as the solvent, and the coupling constants of these two compounds were calculated to be 10.4 Hz. On the basis of this evidence and literature comparison, the structures of **3** and **1** were determined to be (*Z*)-6-oxo-[10]-shogaol and (*Z*)-6-oxo-[6]-shogaol, respectively.

Compound **4** was obtained as brown oil. The molecular formula of **4** is assigned as C_17_H_24_O_4_ on the basis of HRESIMS (*m*/*z* 315.1572 [M + Na]^+^, calcd. for 315.1573). The NMR data (Table 1) of **4** closely resemble those of **1**; the analysis of the ^1^H-NMR and HSQC data of **1** revealed that the significant differences in **4** are the absence of two olefinic protons at C-4 and C-5 and the presence of two additionally multiplet methylene groups at *δ*_H_ 2.65 and *δ*_H_ 2.62. This indicates that the 1,4-enedione-like moiety in **1** was replaced by a 1,4-dicarbonyl-like group in **4**. This deduction was supported by the HMBC correlations from the methylene protons, H-4 and H-5 (*δ*_H_ 2.65 and 2.62), to two carbonyl carbons (*δ*_C_ 209.7, C-3 and 208.7, C-6), from H-1 (*δ*_H_ 2.73) and H-2 (*δ*_H_ 2.80), to C-3 and from H-7 (*δ*_H_ 2.42), and H-8 (*δ*_H_ 1.54) to C-6 (Figure 2). Accordingly, the structure of **4** was further confirmed by the combined analyses of HSQC and HMBC data, and elucidated as 6-oxo-[6]-paradol.

**Figure 3:**
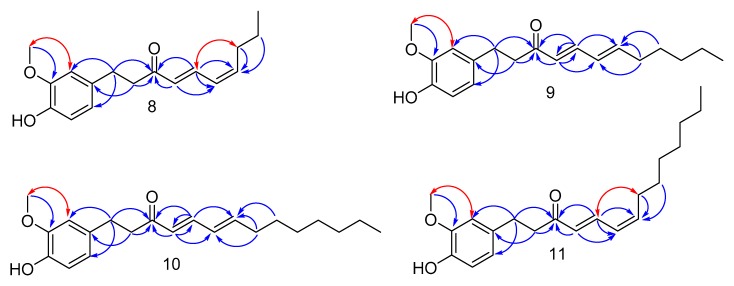
Key HMBC (blue) and NOESY (red) correlations of compounds **8**–**11**.

Compound **5** was obtained as yellow oil. Its molecular formula, C_15_H_20_O_3_, was established by HRESIMS at *m*/*z* 271.1312 for the [M + Na]^+^ (calcd. for 271.1310). The ^1^H-NMR spectrum (Table 2) of **5** exhibited the 1,3,4-tetrasubstituted aromatic moiety [*δ*_H_ 6.82(1H, d, *J* = 8.5 Hz, H-5′), 6.65 (1H, dd, *J* = 8.5, 1.9 Hz, H-6′), 6.64 (1H, s, H-2′)], two *trans*-conformational olefinic protons [*δ*_H_ 6.81(1H, m, H-3), 6.10 (1H, dd, *J* = 16.0, 1.5 Hz, H-4)], a methoxy group [*δ*_H_ 3.85 (3H, s, 3′-OCH_3_)], four methylene groups [*δ*_H_ 2.70 (2H, t, *J* = 7.2 Hz, H-1), 2.48 (2H, t, *J* = 7.2 Hz, H-2), 2.47 (2H, t, *J* = 7.0 Hz, H-6) and 1.61 (2H, m, H-7)], and a methyl group [*δ*_H_ 0.83 (3H, t, *J* = 7.4 Hz, H-8) ]. The ^13^C-NMR and HSQC spectra of **5** revealed the presence of a carbonyl carbon [*δ_C_* 200.7 (C-5) ], two olefinic carbons [*δ*_C_ 145.9 (C-3) and *δ*_C_ 130.7 (C-4) ], six aromatic carbons [*δ*_C_ 146.4 (C-3′), 143.9 (C-4′), 132.7 (C-1′), 120.9 (C-6′), 114.3 (C-5′) and 110.9 (C-2′)], a methoxy carbon [*δ*_C_ 55.85 (3′-OCH_3_)], four methylene carbons [*δ*_C_ 42.0 (C-6), 34.47 (C-2), 34.16 (C-1), and 17.5 (C-7)], a methyl carbon [*δ_C_* 13.8 (C-8)]. The NMR data of **5** are similar to those of [5]-shogaol [15], a typical α,β-unsaturated ketone-type structure. The significant difference in **5** is that the position of the olefinic carbons have changed. This deduction was supported by the HMBC correlations from the methylene protons, H-1 to C-2 and C-3, from H-2 to C-1, C-3 and C-4 and from H-6 and H-7 to C-5. In addition, H–H COSY correlations between H-2 and H-3 also proved this deduction (Figure 3). Accordingly, the structure of **5** was further confirmed by the combined analyses of HSQC and HMBC data. Thus, the structure of **5** was determined as (*E*)-[4]-isoshogaol.

Compound **9**, obtained as yellow oil has the molecular formula, C_19_H_26_O_3_, based on the HRESIMS showing the [M + Na]^+^ ion at *m*/*z* 325.1780 (calcd. for 325.1782). The ^1^H-NMR data (Table 2) of **9** suggest the presence of a typical pattern of a coupling group of 1,3,4-trisubstituted benzene rings at *δ*_H_ 6.80 (1H, d, *J* = 8.0 Hz, H-5′), 6.68 (1H, d, *J* = 1.5 Hz, H-2′) and, 6.66 (1H, dd, *J* = 8.0, 1.5 Hz, H-6′), four olefinic protons [*δ*_H_ 7.10(1H, m, H-5), 6.14 (1H, m, H-6) at 6.13 (1H, m, H-7) and 6.05 (1H, d, *J* = 15.5 Hz, H-4)], a methoxy group [*δ*_H_ 3.85 (3H, s, 3′-OCH_3_)], six methylene groups [*δ*_H_ 2.85 (2H, m, H-2) at 2.82 (2H, m, H-1), 2.15 (2H, m, H-8), 1.40 (2H, m, H-9) and 1.27 (4H, m, H-10 and H-11)], and a methyl group [*δ*_H_ 0.86 (3H, t, *J* = 7.0 Hz, H-12)]. The ^13^C-NMR spectrum of **9** displays 19 signals, assigned by HSQC data to six aromatics [*δ*_C_ 146.4 (C-3′), 143.8 (C-4′), 133.2 (C-1′), 120.8 (C-6′), 114.3 (C-5′) and 111.1 (C-2′)], six methylene [*δ*_C_ 42.4 (C-2), 33.1 (C-8), 31.3 (C-10), 29.9 (C-1), 28.3 (C-9), 22.4 (C-11)], four olefinic carbons [*δ*_C_ 146.0 (C-7), 143.3 (C-5), 128.8 (C-6), 127.7 (C-4)], one ketone carbonyl [*δ*_C_ 199.9 (C-3)], and two methyls [*δ*_C_ 55.83 (3′-OCH_3_), 14.0 (C-12)], with the former being a methoxy. The linkage of the conjugate double bond moiety to carbonyl carbon (C-3) was determined by the HMBC correlations of H-4 and H-5 to C-3. In addition, the significant chemical shift differences in ^13^C-NMR between C-4 and C-5, C-6 and C-7 also proved this deduction (Figure 3).

A large coupling constant between H-4 and H-5 (*J* = 15.5 Hz) indicated that the two olefinic protons are *trans*-oriented. We made a bold guess to determine the orientation of the other two olefins in the conjugated double bonds. It is well known that a conjugated double bond forms a conjugated π system by resonance hybrid, where the double bond between carbon and carbon tends to become longer and the single bond tends to become shorter; thus, the bond between each carbon has the characteristics of double bonds, and each bond cannot be rotated. Therefore, a NOESY correlation should be effective in this conjugated double bond moiety. So, if H-6 and H-7 are *cis*-oriented, the NOESY correlation between H-5 and H-8 can be observed, but if they are *trans*-oriented, this correlation cannot occur. Hence, the absolute configuration of **9** was determined by detailed analysis of its NOESY spectrum, and we did not observe the NOESY interaction between H-5 and H-8 in the NOESY spectrum; in this case, we speculated that H-6 and H-7 must be *trans*-oriented, and previously reported literature [16,17,18] with similar structures confirms our conjecture. Thus, on the basis of this evidence and literature comparison, the structure of **9** was determined to be (4*E*,6*E*)-[6]-paradoldiene.

The HRESIMS of Compound **10** was obtained as yellow oil and showed a [M + Na]^+^ ion at *m*/*z* 353.2091, consistent with a molecular formula of C_21_H_30_O_3_ (calcd. for 353.2093). The ^1^H- and ^13^C-NMR data (Table 2) of **10** closely resemble those of **9**, with a slight difference in the number of carbons in the aliphatic region of the NMR spectra. From this, it can be seen that the side chain of compound **10** should have two more methylene groups than that of compound **9**. Thus, the structure of **10** was determined to be (4*E*,6*E*)-[8]-paradoldiene.

Compound **8** was obtained as yellow oil, and has the molecular formula, C_17_H_22_O_3_, by HRESIMS [found *m*/*z* 297.1465 (calcd. for 297.1467) [M + Na]^+^].The ^1^H- and ^13^C-NMR spectroscopic data (Table 2) of **8** are similar to those of **9**. The main difference is the chemical shift of the carbons and protons of the conjugated double bond. A large coupling constant between H-4 and H-5 (*J* = 15.3 Hz) indicated that the two protons were *trans*-oriented, and a smaller coupling constant between H-6 and H-7 (*J* = 11 Hz) indicated that the two protons were *cis*-oriented. In addition, the NOESY correlation of H-5 and H-8 demonstrates that H-6 and H-7 are *cis*-oriented (Figure 3). Thus, the structure of **8** was determined to be (4*E*,6*Z*)-[4]-paradoldiene.

Compound **11** was obtained as yellow oil and has the same molecular formula (C_21_H_30_O_3_) as **10** by HRESIMS analysis. The ^1^H- and ^13^C-NMR data (Table 2) of **11** closely resemble those of **8**, with a slight difference in the number of carbons in the aliphatic region of the NMR spectra. From this, it can be seen that the side chain of compound **11** should have four more methylene groups than that of compound **8**. In addition, the NOESY correlation of H-2′ and 3′-OCH_3_ also demonstrates that the methoxy is located at C-3′ (Figure 3). Thus, the structure of **11** was determined to be (4*E*,6*Z*)-[8]-paradoldiene.

Known metabolites were identified as (4*E*)-[4]-shogaol (**12**) [15], (4*E*)-[6]-shogaol (**13**) [15], (4*E*)-[8]-shogaol (**14**) [15], (4*E*)-[10]-shogaol (**15**) [15,19], [8]-paradol (**16**) [15], (1*E*,4*E*)-[6]-dehydroshogaol (**17**) [15] (Figure 1) by comparing their spectroscopic data (^1^H-NMR, ^13^C-NMR, ^1^H-^1^H COSY, HSQC, HMBC, and NOESY in Supplementary Materials) with literature values. In addition, by comparing the spectral data of the new compounds that have been identified, we have identified the following two compounds: (3*E*)-[6]-isoshogaol (**6**) and (4*E*,6*Z*)-[4]-paradoldiene (**7**).

## 3. Experimental Section

### 3.1. General Experimental Procedures

UV spectra were measured on a Thermo EVO 300 spectrophotometer (Thermo Fisher Scientific, Madison, WI, USA). IR spectra were recorded on a Thermo Nicolet IS 10 spectrometer (Thermo Fisher Scientific, Madison, WI, USA). NMR spectra were recorded on a Bruker Avance III 500 spectrometer (Bruker Biospin, Fallanden, Switzerland) with TMS as an internal standard. HRESIMS data were recorded on a Bruker maxis HD Mass Q-TOF LC/MS spectrometer (Bruker Daltonics, Billerica, MA, USA). Preparative HPLC was performed on a Sepuruisi LC-52 instrument (Beijing Sepurusi Scientific Co., Ltd., Beijing, China) with an UV200 detector (Beijing Sepurusi Scientific Co., Ltd., Beijing, China), using a YMC-Pack ODS-A column (250 mm × 20 mm, 5 μm). Column chromatography was undertaken on HP-20 macroporous resin (Mitsubishi Chemical Co., Ltd., Tokyo, Japan), SephadexLH-20 (Amersham Pharmacia, Uppsala, Sweden), ODS (50 μm, YMC Co. Ltd., Kyoto, Japan), and silica gel (200–300 mesh, 100–200 mesh, Qingdao Marine Chemical Inc., Qingdao, China). TLC was carried out with glass that was pre-coated with silica gel GF 254 (Qingdao Marine Chemical Inc.) The human heptocelluar (HepG-2) cell line, human mammary cancer (MCF-7) cell line, and human esophageal cancer (KYSE-150) cell line were purchased from the Institute of Materia Medica, Chinese Academy of Medical Sciences and Peking Union Medical College, Beijing, China.

### 3.2. Plant Material

The rhizoma of *Z. officinale* was collected from Bozhou herbal medicine market in Anhui province, China in November 2014, and the locality of growth was Luoping County, Yunnan Province. It was identified by Prof. Sui-Qing Chen at Henan University of Chinese Medicine. A voucher specimen (No. 20141192A) was deposited at the Department of Natural Medicinal Chemistry, School of Pharmacy, Henan University of Chinese Medicine, Zhengzhou, China.

### 3.3. Extraction and Isolation

The dried rhizoma of *Z. officinale* (50 kg) was crushed into coarse powder and macerated for 1 h with 200 L EtOAc, refluxed at 75 °C (3 × 200 × 1.5 h). After removing the solvent under reduced pressure, the EtOAc extract (4 kg) was dissolved in 60% EtOH and divided into three parts (Fr.1–Fr.3) using Diaion HP-20 macroporous resin column eluted with gradient aqueous EtOH (60%, 80%, and 100%). Fr.1 (1.5 kg) was applied to a silica gel (100–200 mesh) column and eluted successively with CH_2_Cl_2_: MeOH (100:0, 30:1, 20:1, 10:1, 5:1, 2:1, 0:100) to yield seven subfractions (Fr.1.1–Fr.1.7). Fraction Fr.1.1 (500 g) was subjected to another silica gel (200–300 mesh) column eluting with a step-gradient of petroleum ether/acetone (from 100:0 to 0:100) to provide nine subfractions: Fr.1.1.1–Fr.1.1.9. Fr.1.1.4 (80 g) was separated by a MCI gel column (MeOH/H_2_O, 10–70%) to get five fractions (Fr.1.1.4.1–Fr.1.1.4.5), of which, fraction Fr.1.1.4.3 (12 g) was purified by Sephadex LH-20 (MeOH), followed by semi-preparative HPLC (MeCN/H_2_O, 65%, flow rate: 3 mL/min) to yield **17** (21.2 mg, R_t_ = 16.8 min), **2** (11.2 mg, R_t_ = 17.6 min), **13** (33.6 mg, R_t_ = 20.1 min), **16** (18.1 mg, R_t_ = 23.5 min) and **14** (78.5 mg, R_t_ = 43.8 min). Fr.1.1.4.2 (10 g) was chromatographed over a silica gel (200–300 mesh) column, eluted with a step-gradient of petroleum ether/EtOAc (from 100:0 to 0:100) and was further purified with semi-preparative HPLC (MeCN/H_2_O, 55%, flow rate: 3 mL/min) to yield **1** (91.2 mg, R_t_ = 8.9 min), **4** (43.3 mg, R_t_ = 9.6 min), **12** (16.7 mg, R_t_ = 10.7 min) and **3** (27.3 mg, R_t_ = 35.5 min). Fr.1.1.4.5 (9 g) was subjected to a silica gel (400–500 mesh) column, eluted with a step-gradient of petroleum ether/EtOAc (from 100: 0 to 0: 100) to get six fractions (Fr.1.1.4.5.1–Fr.1.1.4.5.6). The Fr.1.1.4.5.3 (1.2 g) was further purified by semi-preparative HPLC (MeCN/H_2_O, 65%, flow rate: 3 mL/min) to yield **5** (22.3 mg, R_t_ = 19.8 min) and **6** (17.6 mg, R_t_ = 14.3 min). Fr.1.1.4.5.1 (1.7 g) was further purified by semi-preparative HPLC (MeCN/H_2_O, 80%, flow rate: 3 mL/min) to yield **7** (28.8 mg, R_t_ = 8.7 min), **8** (34.7 mg, R_t_ = 9.1 min), **9** (59.9 mg, R_t_ = 15.2 min), **10** (71.6 mg, R_t_ = 27.2 min), **11** (55.7 mg, R_t_ = 27.7 min), and **15** (59.1 mg, R_t_ = 35.6 min). 

### 3.4. Spectroscopic and Physical Data

Compound **1**: yellow oil; UV (MeOH) *λ*_max_ (log*ε*) 281 (2.83), 202(3.91) nm; IR (neat) *ν*_max_ 3391, 2957, 1682, 1516, 1272, 1033 cm^−1^. ^1^H (500 MHz, CDCl_3_) and ^13^C (125 MHz, CDCl_3_) data are presented in Table 1. HREIMS *m*/*z*: 313.1414 [M + Na] ^+^ (calcd. for C_17_H_22_O_4_, 313.1416).

Compound **2**: brown oil; UV (MeOH) *λ*_max_ (log*ε*) 281 (3.09), 203 (4.15) (0.2) nm; IR (neat) *ν*_max_ 3407, 2931, 1682, 1516, 1272, 1033 cm^−1^. ^1^H (500 MHz, CDCl_3_) and ^13^C (125 MHz, CDCl_3_) data are presented in Table 1. HREIMS *m*/*z*: 341.1727 [M + Na] ^+^ (calcd. for C_19_H_26_O_4_, 341.1729).

Compound **3**: yellow oil; UV (MeOH) *λ*_max_ (log*ε*) 281 (2.85), 203 (3.95) nm; IR (neat) *ν*_max_ 3372, 2922, 1681, 1521, 1278, 1030 cm^−1^. ^1^H (500 MHz, CDCl_3_) and ^13^C (125 MHz, CDCl_3_) data are presented in Table 1. HREIMS *m*/*z*: 369.2042 [M + Na]^+^ (calcd. for C_21_H_30_O_4_, 369.2042).

Compound **4**: yellow oil; UV (MeOH) *λ*_max_ (log*ε*) 281 (2.85), 206 (3.96) nm; IR (neat) *ν*_max_ 3409, 2956, 1707, 1515, 1268, 1033 cm^−1^. ^1^H (500 MHz, CDCl_3_) and ^13^C (125 MHz, CDCl_3_) data are presented in Table 1. HREIMS *m*/*z*: 315.1572 [M + Na]^+^ (calcd. for C_17_H_24_O_4_, 315.1573).

Compound **5**: yellow oil; UV (MeOH) *λ*_max_ (log*ε*) 281 (2.79), 203 (3.98) nm; IR (neat) *ν*_max_ 3356, 2963, 2940, 1664, 1516, 1273, 1035 cm^−1^. ^1^H (500 MHz, CDCl_3_) and ^13^C (125 MHz, CDCl_3_) data are presented in Table 2. HREIMS *m*/*z*: 271.1312 [M + Na]^+^ (calcd. for C_15_H_20_O_3_, 271.1310).

Compound **8**: yellow oil; UV (MeOH) *λ*_max_ (log*ε*) 279 (3.58), 202 (4.02) nm; IR (neat) *ν*_max_ 3381, 2957, 1631, 1593, 1515, 1271, 1034 cm^−1^. ^1^H (500 MHz, CDCl_3_) and ^13^C (125 MHz, CDCl_3_) data are presented in Table 2. HREIMS *m*/*z*: 297.1465 [M + Na]^+^ (calcd. for C_17_H_22_O_3_, 297.1467).

Compound **9**: yellow oil; UV (MeOH) *λ*_max_ (log*ε*) 279(3.44), 203 (3.93) nm; IR (neat) *ν*_max_ 3427, 2929, 1633, 1595, 1516, 1271, 1033 cm^−1^. ^1^H (500 MHz, CDCl_3_) and ^13^C (125 MHz, CDCl_3_) data are presented in Table 2. HREIMS *m*/*z*: 325.1780 [M + Na]^+^ (calcd. for C_19_H_26_O_3_, 325.1782).

Compound **10**: yellow oil; UV (MeOH) *λ*_max_ (log*ε*) 278 (3.60), 202 (4.07) nm; IR (neat) *ν*_max_ 3368, 2927, 1634, 1595, 1516, 1271, 1034 cm^−1^. ^1^H (500 MHz, CDCL_3_) and ^13^C (125 MHz, CDCl_3_) data are presented in Table 2. HREIMS *m*/*z*: 353.2091 [M + Na]^+^ (calcd. for C_21_H_30_O_3_, 353.2093).

Compound **11**: yellow oil; UV (MeOH) *λ*_max_ (log*ε*) 279 (3.47), 202 (4.15) nm; IR (neat) *ν*_max_ 3358, 2927, 1597, 1515, 1270, 1035 cm^−1^. ^1^H (500 MHz, CDCl_3_) and ^13^C (125 MHz, CDCl_3_) data are presented in Table 2. HREIMS *m*/*z*: 353.2094 [M + Na]^+^ (calcd. for C_21_H_30_O_3_, 353.2093).

### 3.5. Cytotoxic Assay

Tumor cells were maintained in RPMI-1640 medium containing 10% heat-inactivated fetal bovine serum, penicillin (100 U/mL) and streptomycin (100 μg/mL), under humidified air with 5% CO_2_ at 37 °C. Exponentially growing cells were seeded into 96-well tissue culture-treated plates and precultured for 24 h; the cells were treated with serum-free medium containing various concentrations of the compounds. After 48 h of incubation, 20 μL of MTT (5 mg/mL in PBS) was added to each well. The cells were incubated at 37 °C for 4 h. After removal of the medium, the cells were treated with 100 μL dimethyl sulfoxide (DMSO) for 10 min, and then the optical density was measured at 570 nm using a microplate reader (iMARK TM microplate reader, Bio-Rad, Hercules, CA, USA). The cytotoxic activities of isolated compounds were tested against MCF-7, HepG-2 and KYSE-150 cell lines. The positive control was 5-fluorouracil.

As the major effective constituent of *Z. officinale*, gingerols have attracted considerable interest in the fields of chemistry and biology. A rising number of papers have reported the biological activities of gingerols, especially on their antitumor activity [2,3,4,5,6,7,8]. Among these compounds, 6-oxo-shogaol derivatives, paradoldiene derivatives and 6-oxo-paradol derivatives are three rare kinds of structures and there are few reports of their biological activities. 

We tested all the newly isolated compounds against HepG-2, MCF-7 and KYSE-150 tumor cells (Table 3). The 6-oxo-shogaol derivatives showed cytotoxic activities against both HepG-2 and MCF-7 cells. Among them, (*Z*)-6-oxo-[6]-shogaol (**1**) was the most prominent one, in terms of its cytotoxic activities, whereas this kind of compound has little cytotoxic effect on the KYSE-150 cell line. In addition, we also found that the cytotoxic activities of these compounds against HepG-2 and MCF-7 cells gradually decreased with an increase in the carbon chain. In contrast, among the paradoldiene derivatives, only (4*E*,6*Z*)-[4]-paradoldiene (**8**) showed better activity against HepG-2 and MCF-7 cells, while the other compounds had no obvious effects on these three kinds of cells. It indicated that with an increase in the carbon chain, the cytotoxic activities of HepG-2 and MCF-7 cells decreased dramatically. We only isolated one 6-oxo-paradol derivative, 6-oxo-[6]-paradol (**4**), which was very similar to the structure of (*Z*)-6-oxo-[6]-shogaol (**1**), with only one double bond between the carbonyl groups on the carbon chain, but it had no significant activity against these three tumor cells. We also isolated one isoshogaol derivative, [4]-isoshogaol (**5**), which was found to produce better activity on all three tested cells. 

### 3.6. Statistical Analysis

The results of each group are expressed as mean ± SD values. Data were analyzed through the one-way ANOVA between control and sample treated groups, in Microsoft Excel. A statistically significant difference among groups was considered to be represented by *p* < 0.05.

## 4. Conclusions

In-depth research on ginger has been conducted by scholars across the world. In addition, gingerols, the main component of ginger, have been studied and applied in many fields. Though gingerols contain many varieties, it is not an easy task to find new gingerols. In this experiment, nine new compounds were isolated, and among them, 6-oxo-shogaol, 6-oxo-paradol and paradoldiene are rare structures in gingerol derivatives. Moreover, isolation of 6-oxo-shogaol derivatives from the rhizoma of *Z. officinale* is reported here for the first time, which also enriches our knowledge about the chemical diversity of this plant. 

A wide range of gingerol derivatives, and many kinds of activities have been reported [1,2,3,4,5,6,7,8,9,10]. However, the 6-oxo-shogaol derivatives, paradoldiene derivatives and 6-oxo-paradol derivatives were rarely investigated, and the tested activity of these three derivatives against HepG-2, MCF-7 and KYSE-150 cells is reported here for the first time. To evaluate the efficacy of these nine compounds, the MTT cytotoxicity assay was performed, and significant inhibitory effects were found on the proliferation of these three kinds of cell lines. By comparing the activity of these three kinds of derivatives, we hypothesized that the α,β-unsaturated ketone structure is a key construction unit to cytotoxic activities of gingerol compounds, and the cytotoxic activity decreases rapidly with an increase in the carbon chain.

Our research enriched the number of types of gingerol derivatives, and these results will broaden the application field of gingerols.

## Figures and Tables

**Table 1:** ^1^H- (500 MHz) and ^13^C-NMR (125 MHz) data for compounds **1**–**4** in CDCl_3_ (*δ* in ppm, *J* in Hz).

	1		2		3		4	
Position	*δ*_C_	*δ*_H_	*δ*_C_	*δ*_H_	*δ*_C_	*δ*_H_	*δ*_C_	*δ*_H_
1′	132.4		132.4		132.4		132.9	
2′	111.0	6.67, d (1.5)	111.0	6.67, d (1.5)	111.0	6.67, s	111.0	6.66, d (1.9)
3′	146.4		146.4		146.4		146.4	
4′	144.0		144.0		144.0		143.9	
5′	114.4	6.81, d (8.0)	114.4	6.81, d (8.0)	114.4	6.80, d (8.0)	114.2	6.79, d (8.0)
6′	120.8	6.65, dd (8.0,1.5)	120.8	6.66, dd (8.0,1.5)	120.8	6.65, d (8.0)	120.7	6.62, dd (8.0,1.9)
1	29.3	2.86, m	29.3	2.86, m	29.3	2.88, m	29.4	2.73, m
2	43.4	2.92, m	43.5	2.94, m	43.5	2.92, m	44.6	2.80, m
3	200.7		200.7		200.7		208.8	
4	136.4	6.82, s	136.4	6.83, s	136.4	6.83, s	36.1	2.65, m
5	136.0	6.82, s	136.1	6.83, s	136.0	6.83, s	35.9	2.62, m
6	199.8		199.8		199.8		209.7	
7	41.3	2.59, t (7.4)	41.7	2.59, t (7.4)	41.6	2.58, t (7.4)	42.5	2.42, t (7.4)
8	25.7	1.58, m	23.7	1.60, m	23.7	1.59, m	25.9	1.54, m
9	22.1	1.31, m	28.8	1.28, m	29.0	1.24, m	22.	1.27, m
10	13.7	0.88, t (7.3)	31.5	1.28, m	29.3	1.24, m	13.8	0.87, t (7.4)
11			22.4	1.28, m	29.3	1.24, m		
12			14.0	0.85, t (6.4)	31.7	1.24, m		
13					22.3	1.24, m		
14					14.0	0.85, t (6.4)		
3′-OCH_3_	55.8	3.85, s	55.9	3.85, s	55.8	3.85, s	55.8	3.85, s

**Table 2:** ^1^H- (500 MHz) and ^13^C-NMR (125 MHz) data for compounds **5** and **8**–**11** in CDCl_3_ (*δ* in ppm, *J* in Hz).

	5		8		9		10		11	
Position	*δ*_C_	*δ*_H_	*δ*_C_	*δ*_H_	*δ*_C_	*δ*_H_	*δ*_C_	*δ*_H_	*δ*_C_	*δ*_H_
1′	132.7		133.1		133.2		133.2		133.2	
2′	110.9	6.64, s	111.1	6.69, d(1.5)	111.1	6.68, d(1.5)	111.1	6.69, d(1.6)	111.1	6.68, d (1.5)
3′	146.4		146.4		146.4		146.4		146.4	
4′	143.9		143.8		143.8		143.8		143.9	
5′	114.3	6.82, d (8.5)	114.3	6.81, d (8.0)	114.3	6.80, d (8.0)	114.3	6.80, d (8.0)	114.3	6.81, d (8.0)
6′	120.9	6.65, dd (8.5, 1.9)	120.7	6.66, dd (8.0, 1.5)	120.8	6.66, dd (8.0, 1.5)	120.7	6.66, dd (8.0, 1.6)	120.8	6.66, dd (8.0, 1.5)
1	34.2	2.70, t (7.2)	29.9	2.83, m	29.9	2.82, m	29.9	2.82, m	29.9	2.83, m
2	34.5	2.48, t (7.2)	42.8	2.85, m	42.4	2.85, m	42.3	2.85, m	42.90	2.85, m
3	145.9	6.81, m	200.0		199.9		199.9		199.9	
4	130.7	6.10, dd (16.0,1.5)	129.3	6.14, d (15.3)	127.7	6.05, d (15.5)	127.7	6.05, d (15.5)	129.3	6.14, d (15.5)
5	200.7		137.4	7.48, m	143.3	7.10, m	143.4	7.10, m	137.4	7.45, m
6	42.0	2.47, t (7.0)	127.0	6.11, q (11.2)	128.8	6.14, m	128.7	6.14, m	126.8	6.09, q (11.0)
7	17.5	1.61, m	142.6	5.89, m	146.0	6.13, m	146.0	6.13, m	143.0	5.88, m
8	13.8	0.83, t (7.4)	30.3	2.25, m	33.1	2.15, m	33.1	2.15, m	29.3	2.26, q (7.5)
9			22.5	1.42, m	28.3	1.40, m	28.6	1.40, m	28.3	1.38, m
10			13.6	0.90, t (7.4)	31.3	1.27, m	29.1	1.25, m	29.1	1.25, m
11					22.4	1.27, m	29.0	1.25, m	29.0	1.25, m
12					14.0	0.86, t (7.0)	31.6	1.25, m	31.7	1.25, m
13							22.6	1.25, m	22.6	1.25, m
14							14.0	0.86, t (6.7)	14.1	0.85, t (6.1)
3′-OCH_3_	55.85	3.85	55.81	3.85, s	55.83	3.8, s	55.8	3.85, s	55.85	3.85, s

**Table 3:** Cytotoxicities of compounds **1**–**5** and **13**–**16** against MCF-7, HepG-2 and KYSE-150 cell lines (IC_50_, μM).

Compound	IC_50_ (μM)		
	HepG-2	Mcf-7	KYSE-150
**1**	8.92 ± 0.34	6.27 ± 0.21	>50
**2**	45.14 ± 1.69	47.22 ± 2.31	>50
**3**	>50	>50	>50
**4**	>50	>50	>50
**5**	14.87 ± 0.57	>50	>50
**13**	21.56 ± 1.47	22.85 ± 1.01	20.41 ± 0.53
**14**	>50	>50	>50
**15**	>50	>50	>50
**16**	>50	>50	>50
5-Fluorouracil	8.18 ± 0.53	7.35 ± 0.37	13.26 ± 0.47

## Supplementary Materials

The following are available online.
