# Supplementary material for: Nine New Gingerols from the Rhizoma of Zingiber officinale and Their Cytotoxic Activities
Source: Molecules. 2018 Feb 2;23(2):315. doi: 10.3390/molecules23020315 (PMC6017651; doi:10.3390/molecules23020315)

Nine new Gingerols from the rhizoma of *Zingiber officinale* Rosc. and their cytotoxic activities.

Zezhi Li[a], Yanzhi Wang[a,b,*], MeiLing Gao[a], Wanhua Cui[a], Mengnan Zeng[a], Juan Li[a]

a. School of Pharmacy, Henan University of Chinese Medicine, Zhengzhou 450046, People's Republic of China.

b. Collaborative Innovation Center for Respiratory Disease Diagnosis, Treatment and New Drug Research and Development of Henan Province, Henan University of Chinese Medicine, Zhengzhou 450046, People's Republic of China.

Supporting Information

*Corresponding authors at: School of Pharmacy, Henan University of Chinese Medicine, Zhengzhou 450046, People's Republic of China.

E-mail addresses: wangyzlb@126.com (Y.-Z. Wang).

For compound 1

S1. ^{1}H NMR spectrum of **1** (500MHz, $CDCL_3$)
S2. ^{13}C NMR spectrum of **1** (125MHz, $CDCL_3$)
S3. HSQC spectrum of **1**
S4. HMBC spectrum of **1**
S5. ^{1}H NMR spectrum of **1** (500MHz, C_6D_6)
S6. HRESIMS spectrum of **1**
S7. IR spectrum of **1**
S8. UV spectrum of **1**

For Compound 2

S9. ^{1}H NMR spectrum of **2** (500MHz $_6$, $CDCL_3$)
S10. ^{13}C NMR spectrum of **2** (125MHz, $CDCL_3$)
S11. HSQC spectrum of **2**
S12. HMBC spectrum of **2**
S13. ^{1}H NMR spectrum of **3** (500MHz $_6$, C_6D_6)
S14. HRESIMS spectrum of **2**
S15. IR spectrum of **2**
S16. UV spectrum of **2**

For compound 3

S17. ^{1}H NMR spectrum of **3** (500MHz, $CDCL_3$)
S18. ^{13}C NMR spectrum of **3** (125MHz, $CDCL_3$)
S19. HSQC spectrum of **3**
S20. HMBC spectrum of **3**
S21. NOESY spectrum of **3**
S22. ^{1}H NMR spectrum of **3** (500MHz, C_6D_6)
S23. HRESIMS spectrum of **3**
S24. IR spectrum of **3**
S25. UV spectrum of **3**

For compound 4

S26. ^{1}H NMR spectrum of **4** (500MHz, $CDCL_3$)
S27. ^{13}C NMR spectrum of **4** (125MHz, $CDCL_3$)
S28. DEPT135 spectrum of **4**
S29. HSQC spectrum of **4**
S30. HMBC spectrum of **4**
S31. ^{1}H-^{1}H COSY spectrum of **4**
S32. HRESIMS spectrum of **4**
S33. IR spectrum of **4**
S34. UV spectrum of **4**

For compound 5

S35. ^{1}H NMR spectrum of **5** (500MHz, $CDCL_3$)

S36. ^{13}C NMR spectrum of **5** (125MHz, $CDCL_3$)
S37. HSQC spectrum of **5**
S38. HMBC spectrum of **5**
S39. ^{1}H-^{1}H COSY spectrum of **5**
S40. HRESIMS spectrum of **5**
S41. IR spectrum of **5**
S42. UV spectrum of **5**

For compound 6
S43. ^{1}H NMR spectrum of **6** (500MHz, $CDCL_3$)
S44. ^{13}C NMR spectrum of **6** (125MHz $_6$, $CDCL_3$)

For compound 7
S45. ^{1}H NMR spectrum of **7** (500MHz, $CDCL_3$)
S46. ^{13}C NMR spectrum of **7** (125MHz, $CDCL_3$)
S47. NOESY spectrum of **7**

For compound 8
S48. ^{1}H NMR spectrum of **8** (500MHz, $CDCL_3$)
S49. ^{13}C NMR spectrum of **8** (125MHz, $CDCL_3$)
S50. HSQC spectrum of **8**
S51. HMBC spectrum of **8**
S52. NOESY spectrum of **8**
S53. HRESIMS spectrum of **8**
S54. IR spectrum of **8**
S55. UV spectrum of **8**

For compound 9
S56. ^{1}H NMR spectrum of **9** (500MHz, $CDCL_3$)
S57. ^{13}C NMR spectrum of **9** (125MHz, $CDCL_3$)
S58. HSQC spectrum of **9**
S59. HMBC spectrum of **9**
S60. NOESY spectrum of **9**
S61. HRESIMS spectrum of **9**
S62. IR spectrum of **9**
S63. UV spectrum of **9**

For compound 10
S64. ^{1}H NMR spectrum of **10** (500MHz, $CDCL_3$)
S65. ^{13}C NMR spectrum of **10** (125MHz, $CDCL_3$)
S66. HSQC spectrum of **10**
S67. HMBC spectrum of **10**
S68. NOESY spectrum of **10**
S69. HRESIMS spectrum of **10**

S70. IR spectrum of **10**
S71. UV spectrum of **10**

For compound 11
S72. ^{1}H NMR spectrum of **11** (500MHz, $CDCL_3$)
S73. ^{13}C NMR spectrum of **11** (125MHz, $CDCL_3$)
S74. HSQC spectrum of **11**
S75. HMBC spectrum of **11**
S76. NOESY spectrum of **11**
S77. HRESIMS spectrum of **11**
S78. IR spectrum of **11**
S79. UV spectrum of **11**

For compound 12
S80. ^{1}H NMR spectrum of **12** (500MHz, $CDCL_3$)
S81. ^{13}C NMR spectrum of **12** (125MHz, $CDCL_3$)

For compound 13
S82. ^{1}H NMR spectrum of **13** (500MHz, $CDCL_3$)
S83. ^{13}C NMR spectrum of **13** (125MHz, $CDCL_3$)

For Compound 14:
S84. ^{1}H NMR spectrum (500MHz, $CDCL_3$) of **14**
S85. ^{13}C NMR spectrum (125MHz, $CDCL_3$) of **14**

For Compound 15:
S86. ^{1}H NMR spectrum (500MHz, $CDCL_3$) of **15**
S87. ^{13}C NMR spectrum (125MHz, $CDCL_3$) of **15**

For compound 16
S88. ^{1}H NMR spectrum of 1**6** (500MHz, $CDCL_3$)
S89. ^{13}C NMR spectrum of 1**6** (125MHz, $CDCL_3$)

For Compound 17:
S90. ^{1}H NMR spectrum (500MHz, $CDCL_3$) of **17**
S91. ^{13}C NMR spectrum (125MHz, $CDCL_3$) of **17**

S1. ^{1}H NMR spectrum of **1** (500MHz, CDCL$_3$)

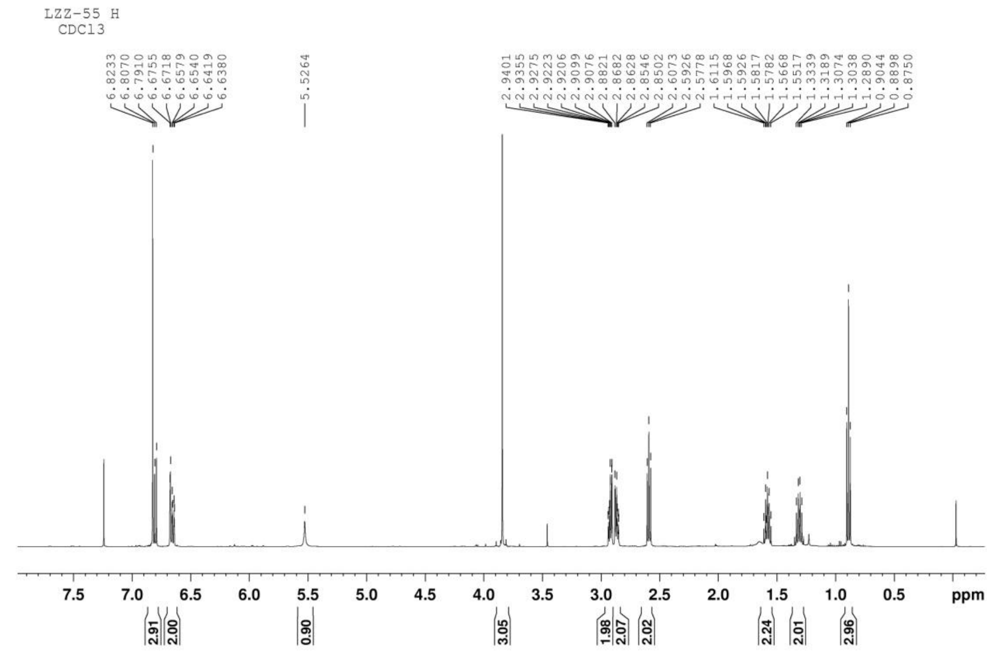

S2. ^{13}C NMR spectrum of **1** (125MHz, CDCL$_3$)

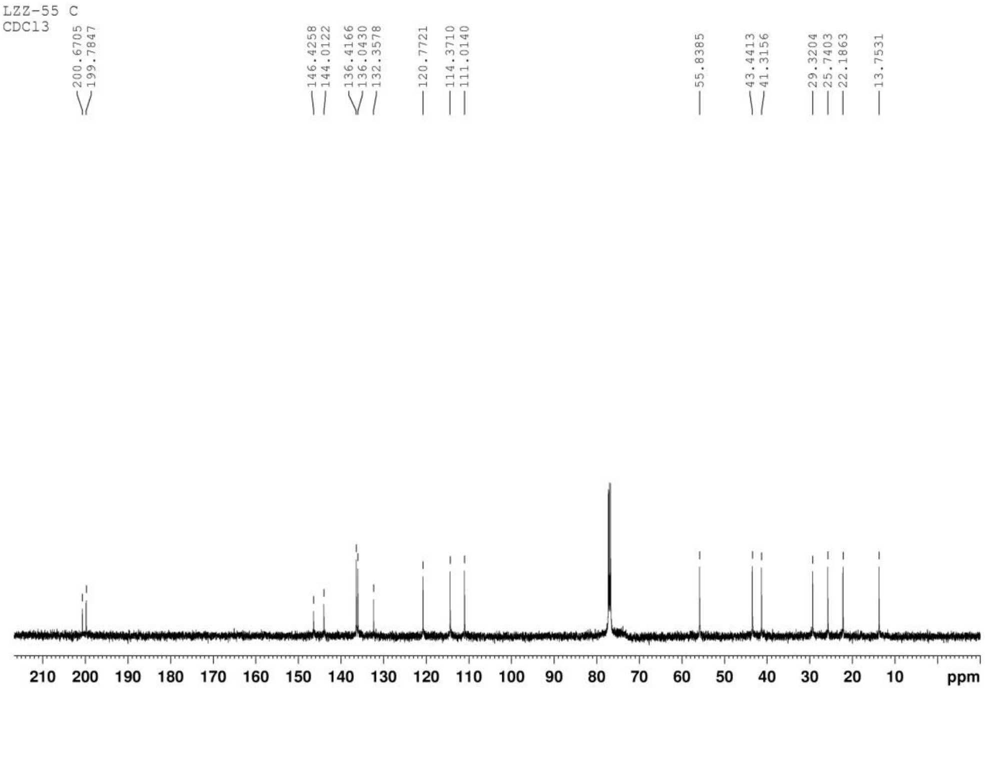

S3. HSQC spectrum of **1**

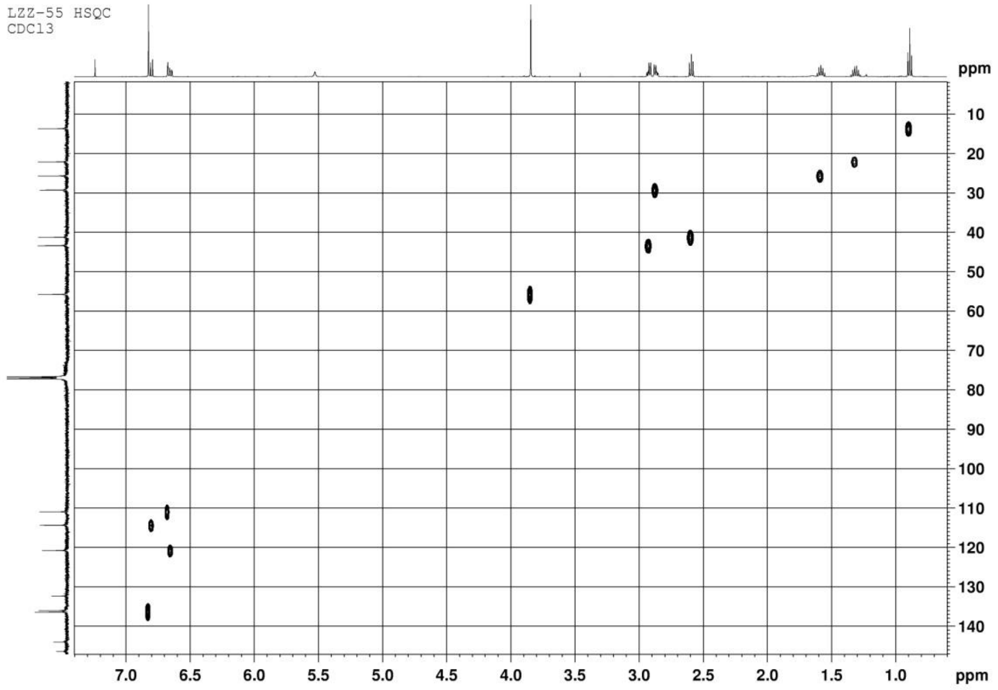

S4. HMBC spectrum of **1**

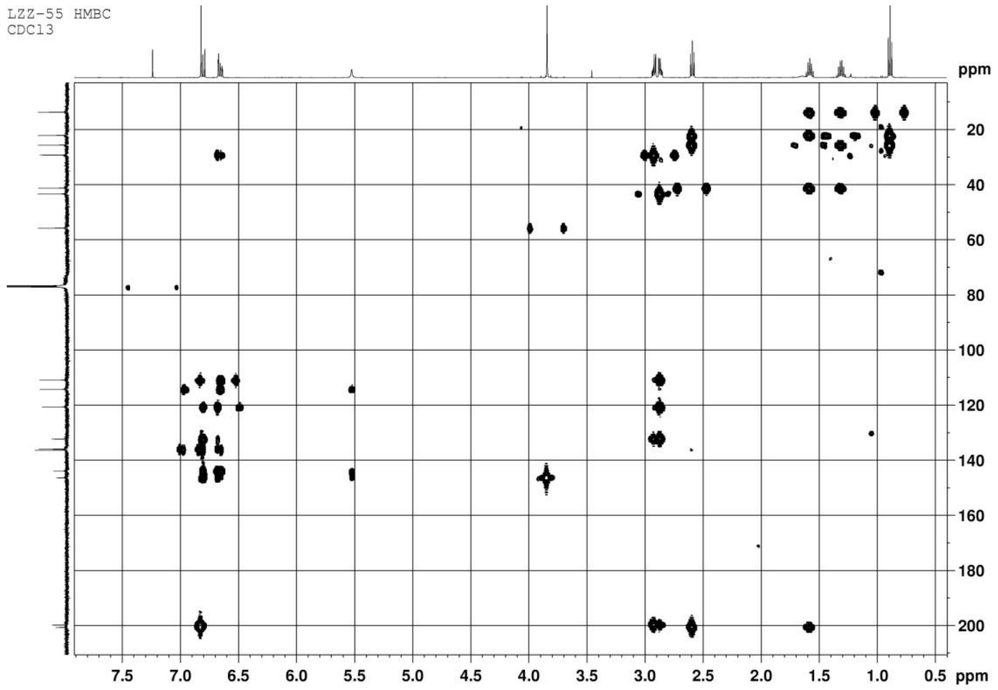

S5. ^{1}H NMR spectrum of **1** (500MHz, C_6D_6)

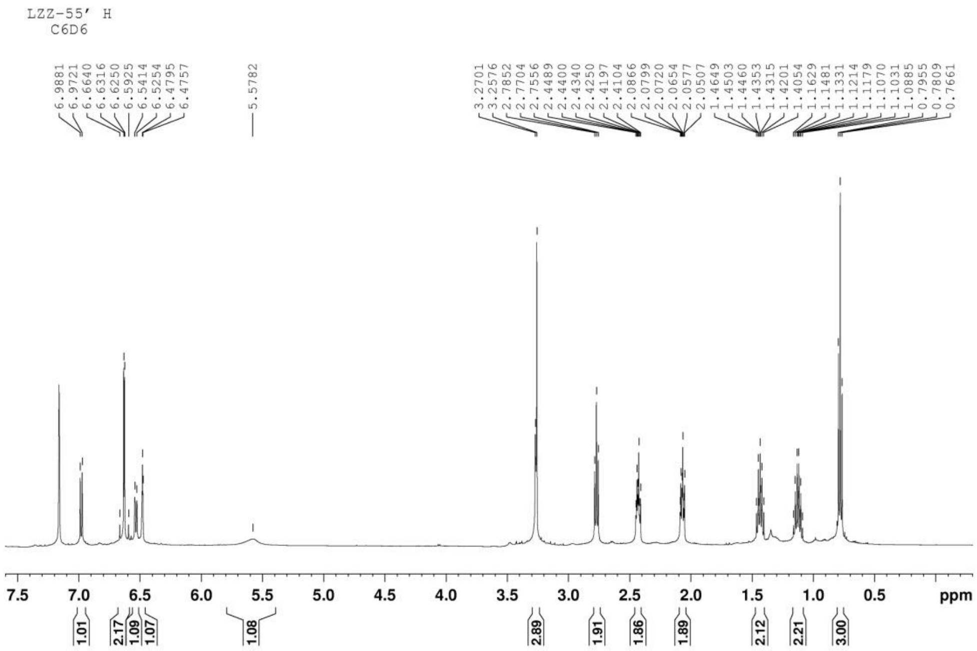

S6. HRESIMS spectrum of **1**

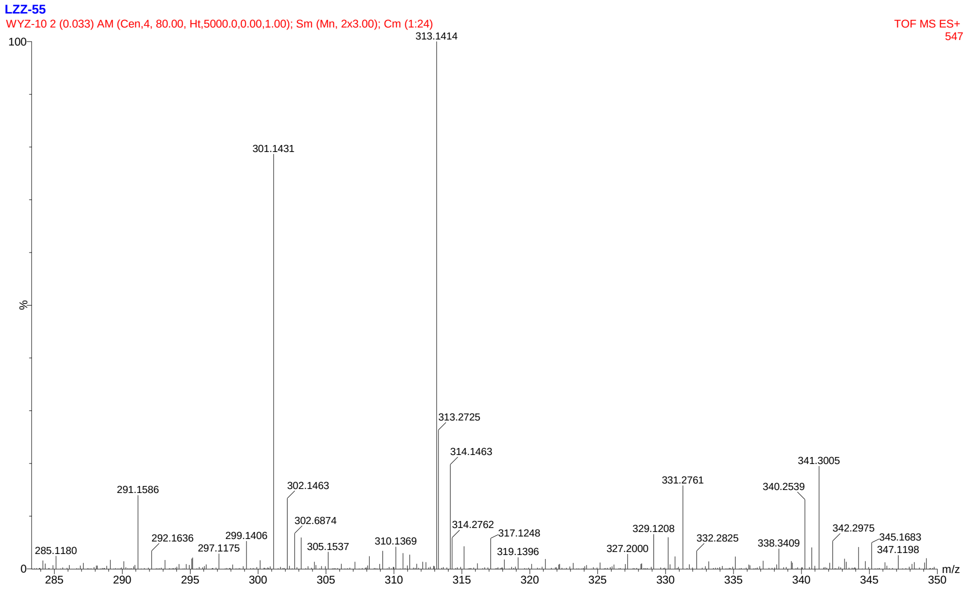

S7. IR spectrum of **1**

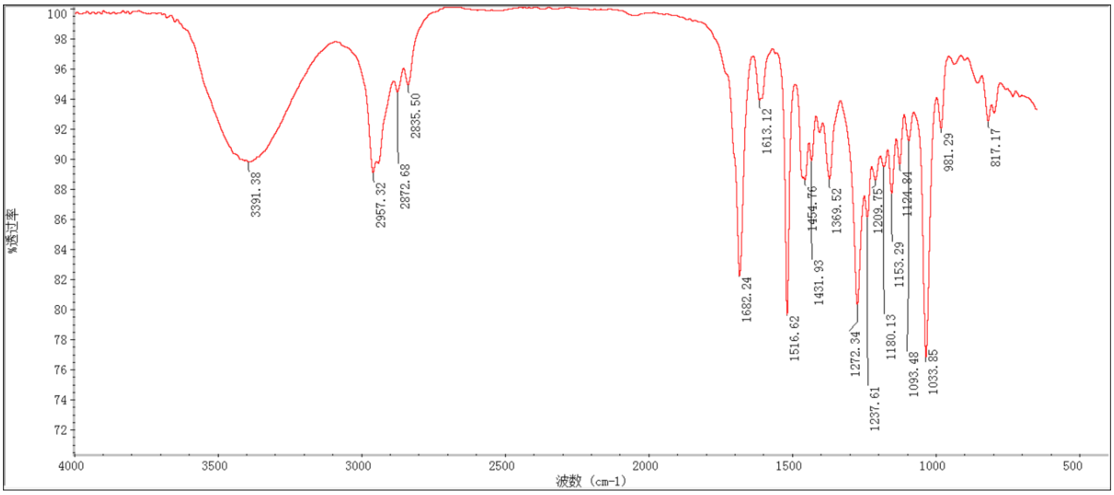

S8. UV spectrum of **1**

Thermo Scientific ~ VISIONpro SOFTWARE V4.41

Operator Name	(None Entered)	Date of Report	2017/11/29
Department	(None Entered)	Time of Report	21:00:54下午
Organization	(None Entered)		
Information	(None Entered)		

Scan Graph

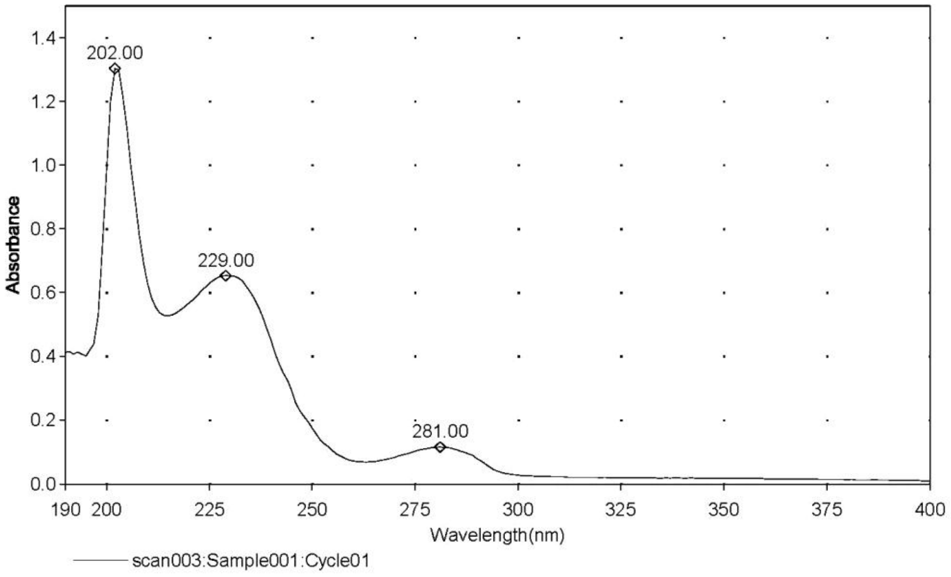

Results Table - scan003,Sample001,Cycle01

nm	A	**Manual Method**
202.00	1.303	Report Values at 3 Wavelengths
229.00	.653	202.00 nm 229.00 nm 281.00 nm
281.00	.115	Sort By Wavelength

Page 1, Scan Graph

S9. ^{1}H NMR spectrum of **2** (500MHz $_{6}$, CDCL$_3$)

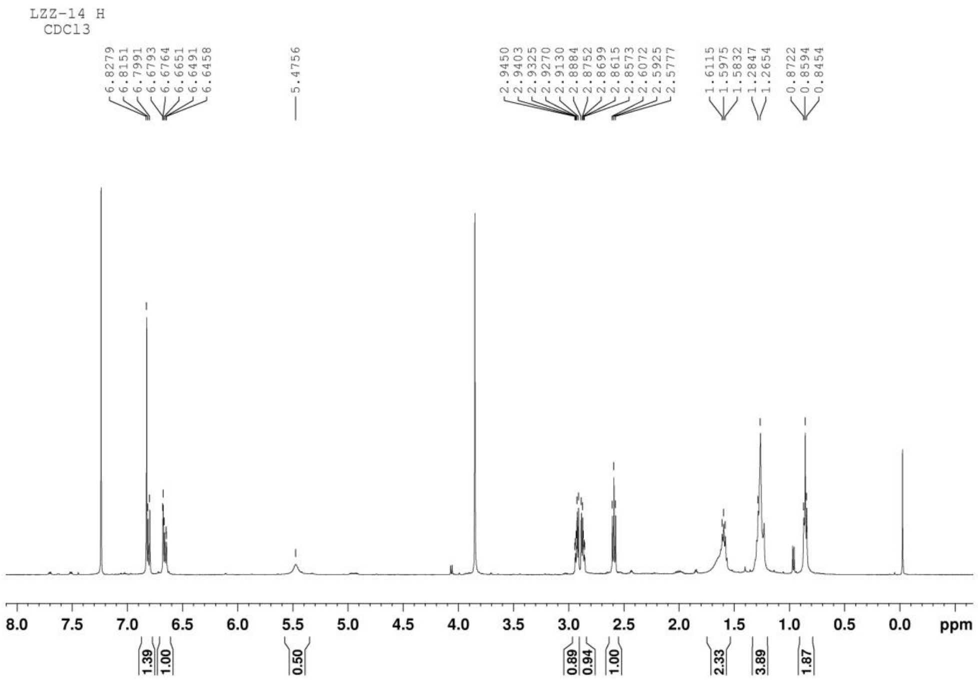

S10. ^{13}C NMR spectrum of **2** (125MHz, CDCL$_3$)

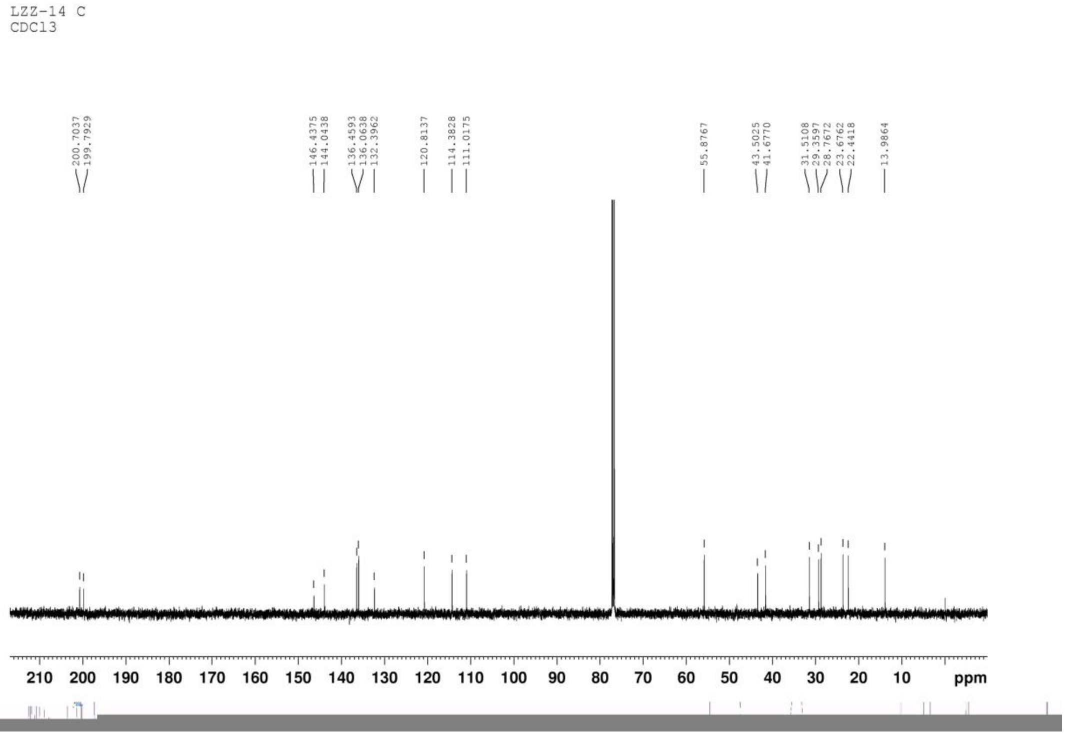

S11. HSQC spectrum of **2**

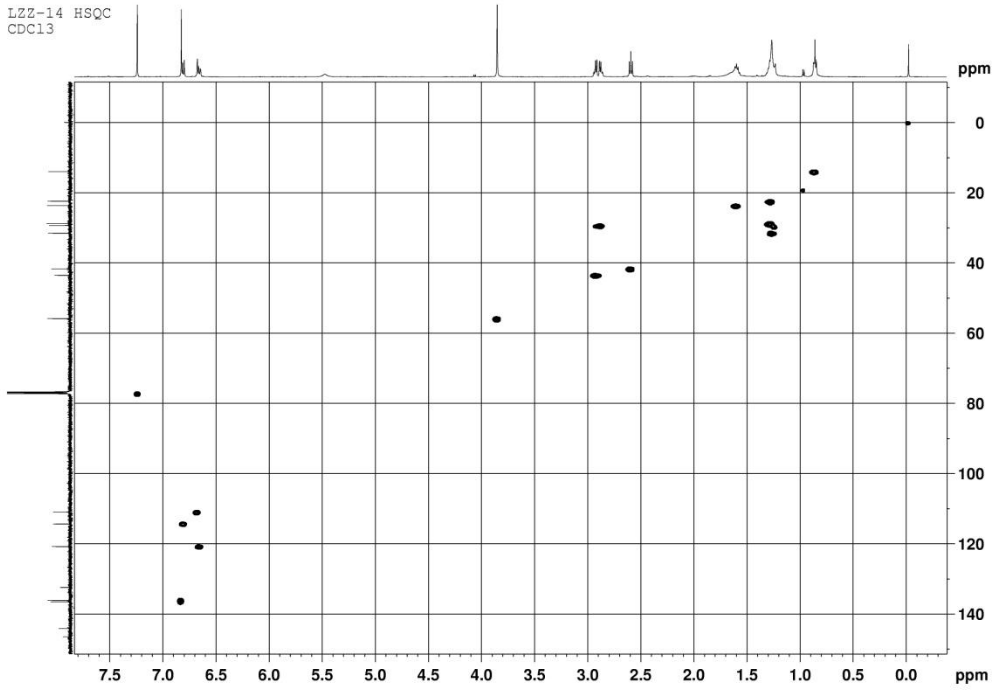

S12. HMBC spectrum of **2**

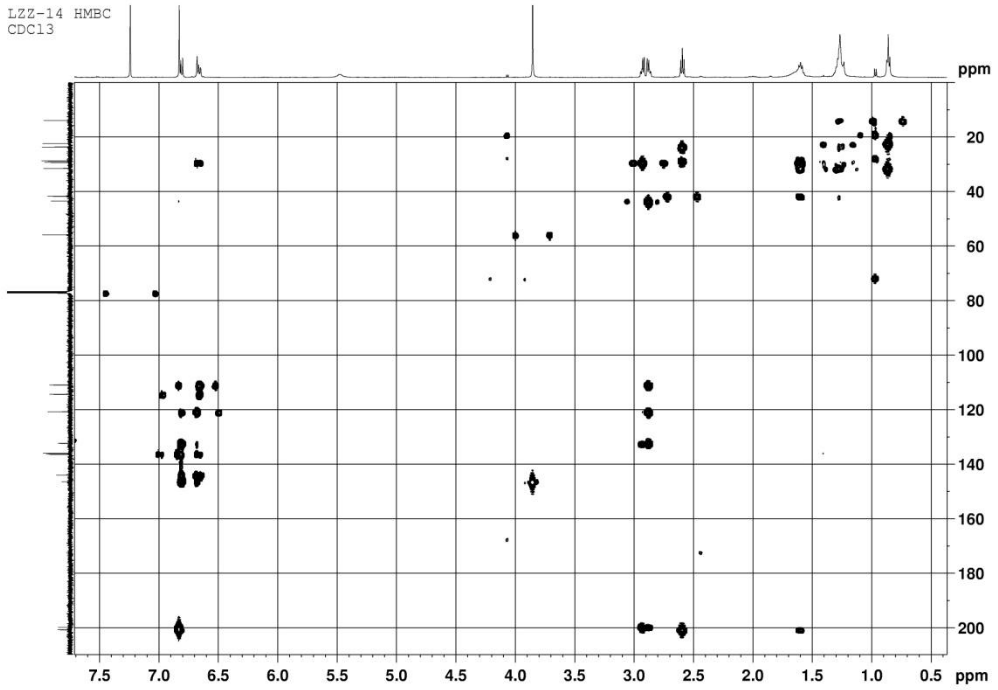

S13. ^{1}H NMR spectrum of **2** (500MHz $_{6}$, C_6D_6)

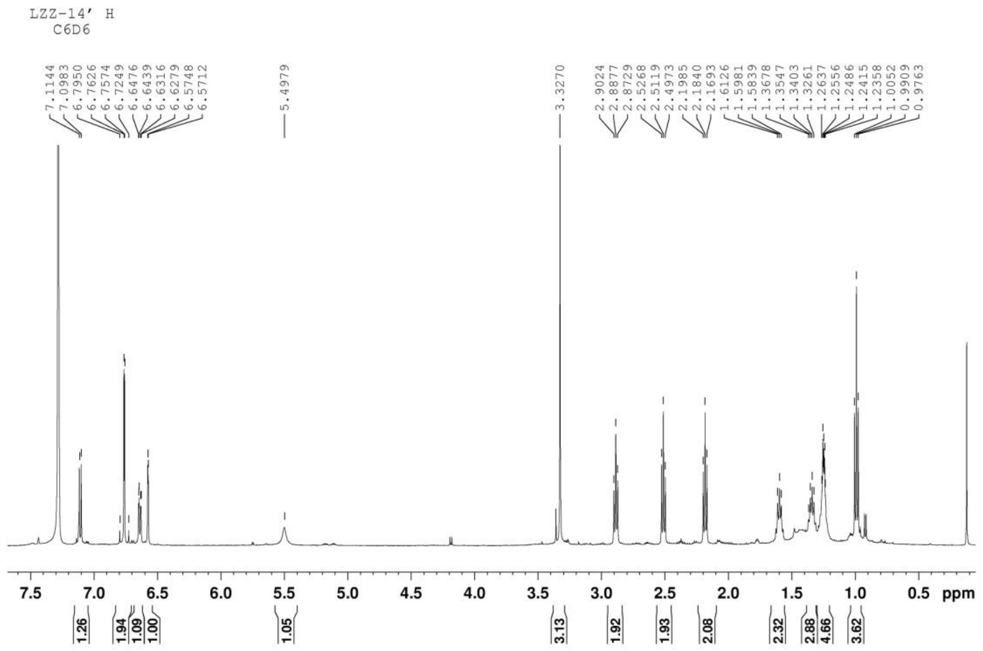

S14. HRESIMS spectrum of **2**

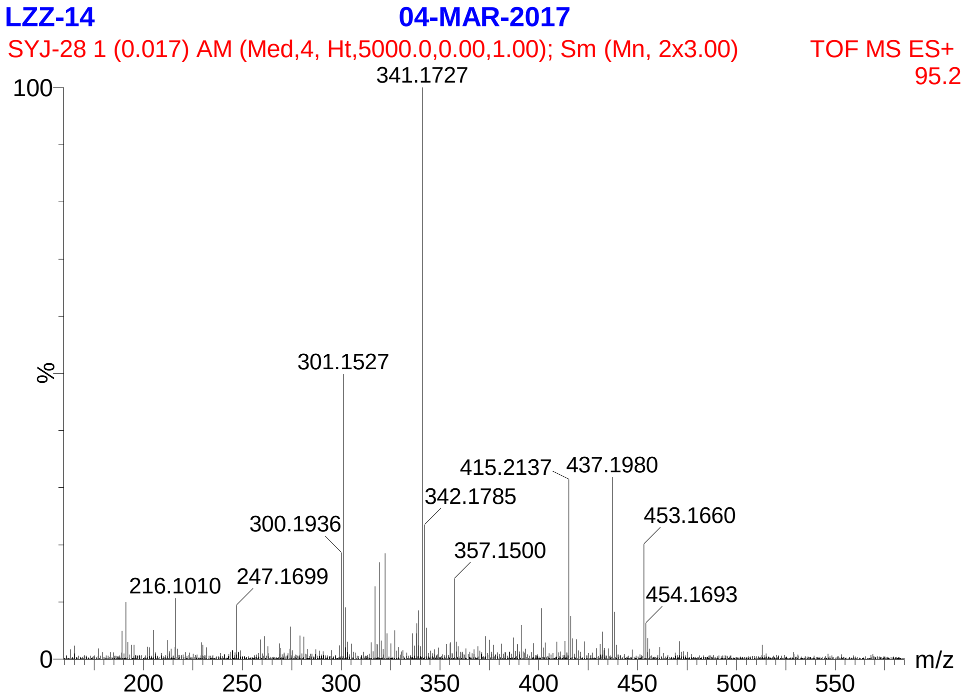

S15. IR spectrum of **2**

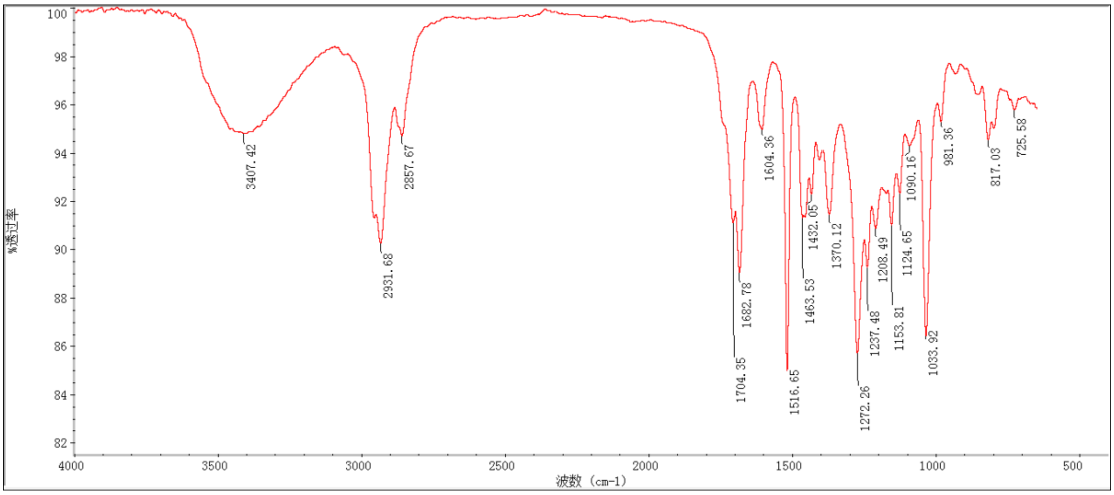

S16. UV spectrum of **2**

Thermo Scientific ~ VISIONpro SOFTWARE V4.41

Operator Name	(None Entered)	Date of Report	2017/11/29
Department	(None Entered)	Time of Report	21:07:00下午
Organization	(None Entered)		
Information	(None Entered)		

Scan Graph

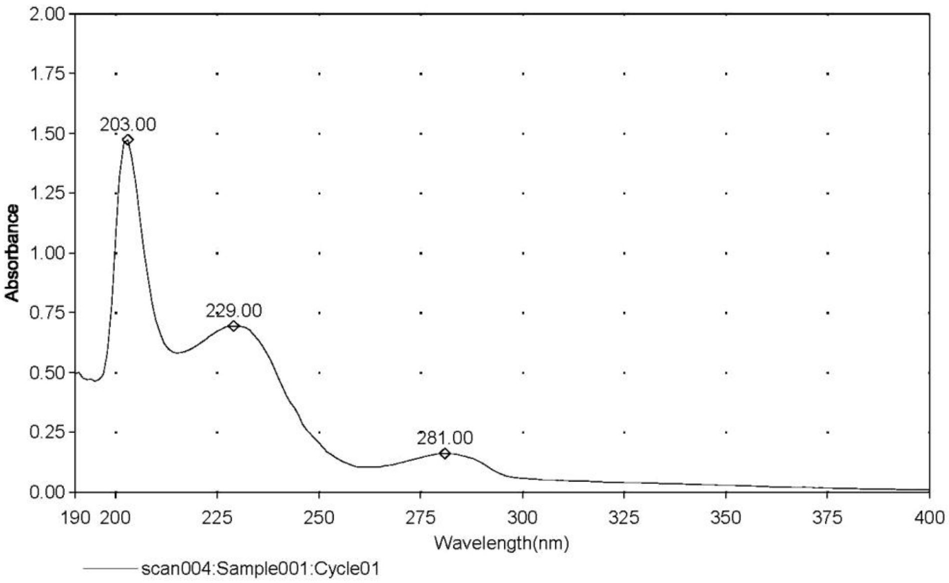

Results Table - scan004,Sample001,Cycle01

nm	A	**Manual Method**
203.00	1.473	Report Values at 3 Wavelengths
229.00	.694	203.00 nm 229.00 nm 281.00 nm
281.00	.160	Sort By Wavelength

S17. ^{1}H NMR spectrum of **3** (500MHz, CDCL$_3$)

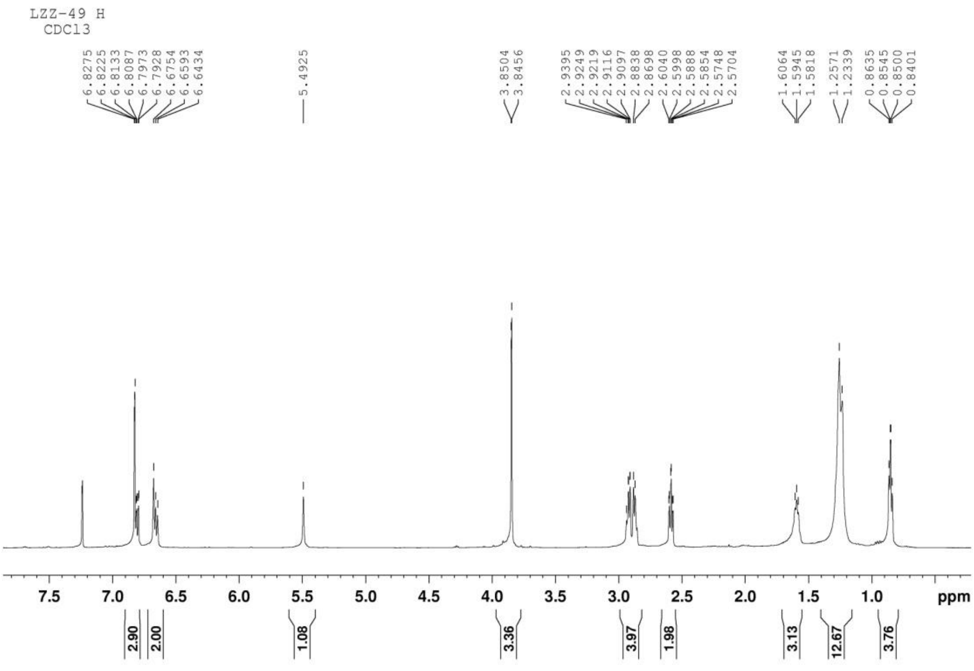

S18. ^{13}C NMR spectrum of **3** (125MHz, CDCL$_3$)

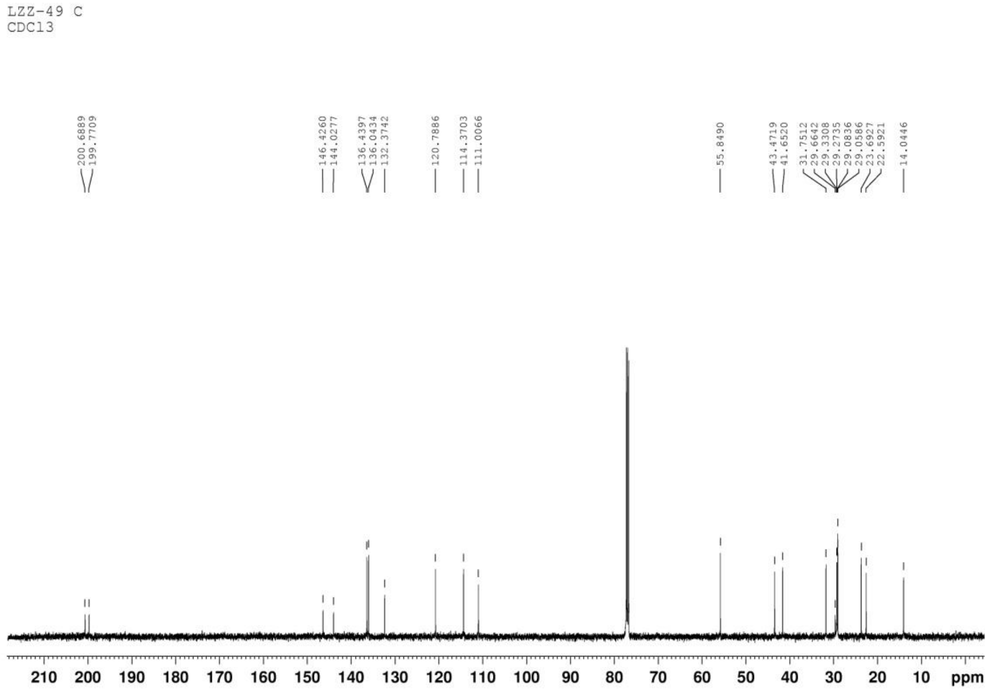

S19. HSQC spectrum of **3**

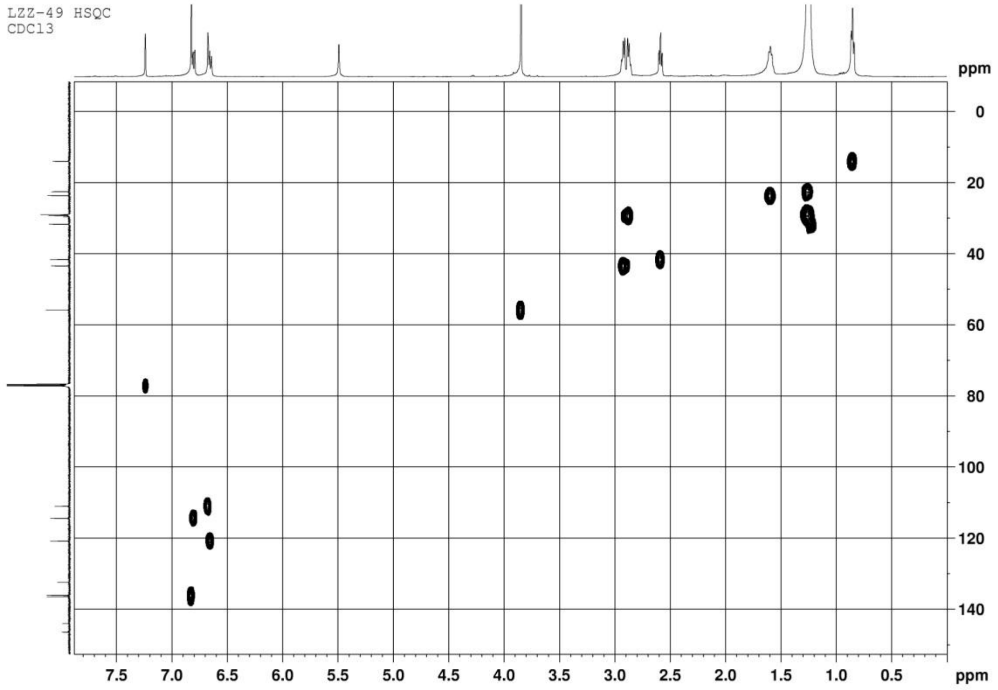

S20. HMBC spectrum of **3**

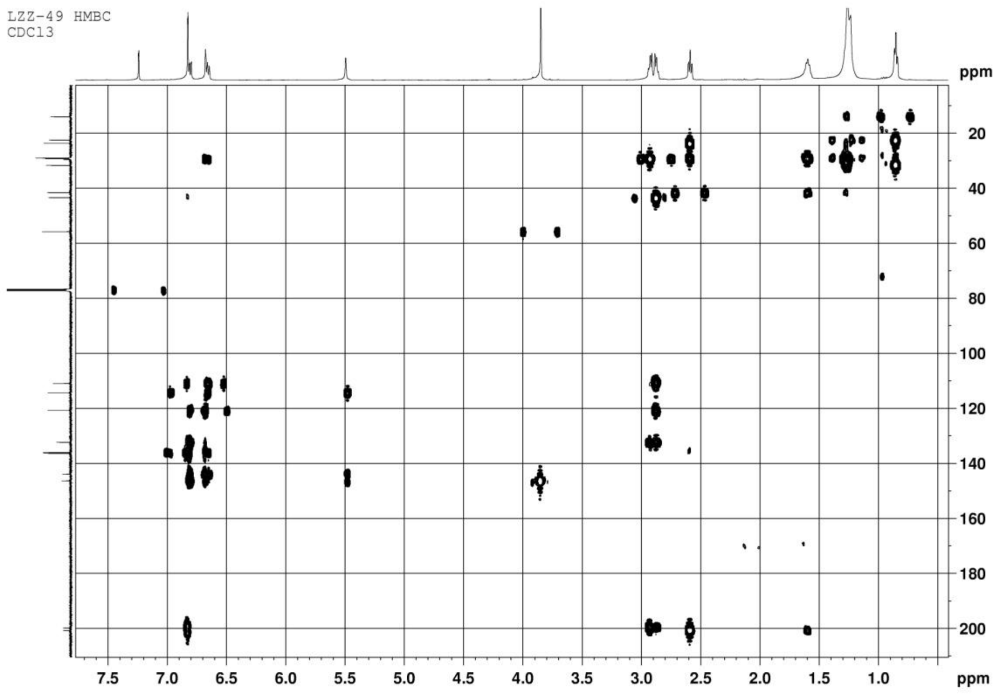

S21. NOESY spectrum of **3**

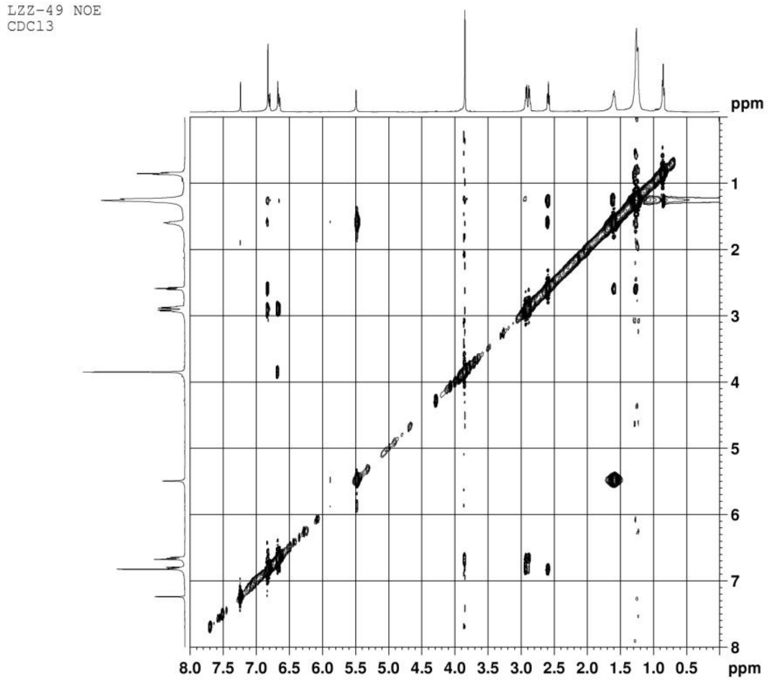

S22. ^{1}H NMR spectrum of **3** (500MHz, C_6D_6)

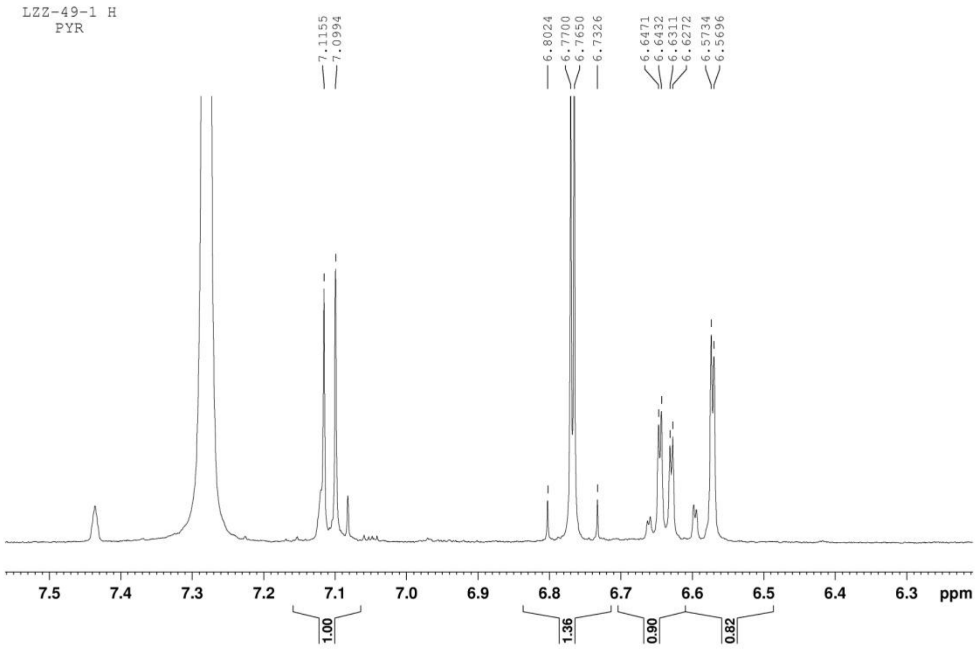

S23. HRESIMS spectrum of **3**

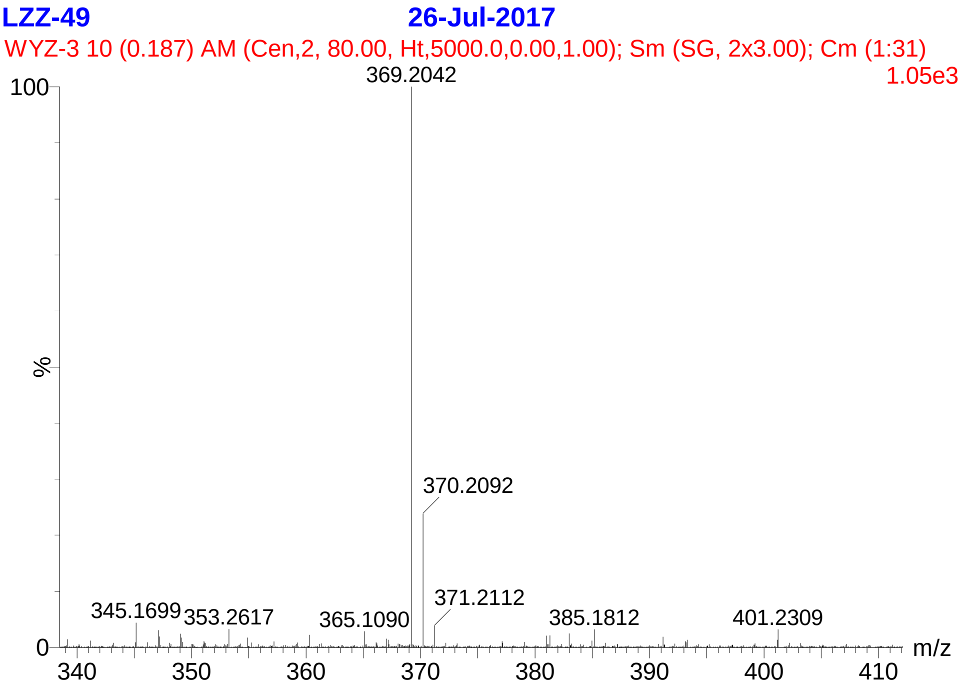

S24. IR spectrum of **3**

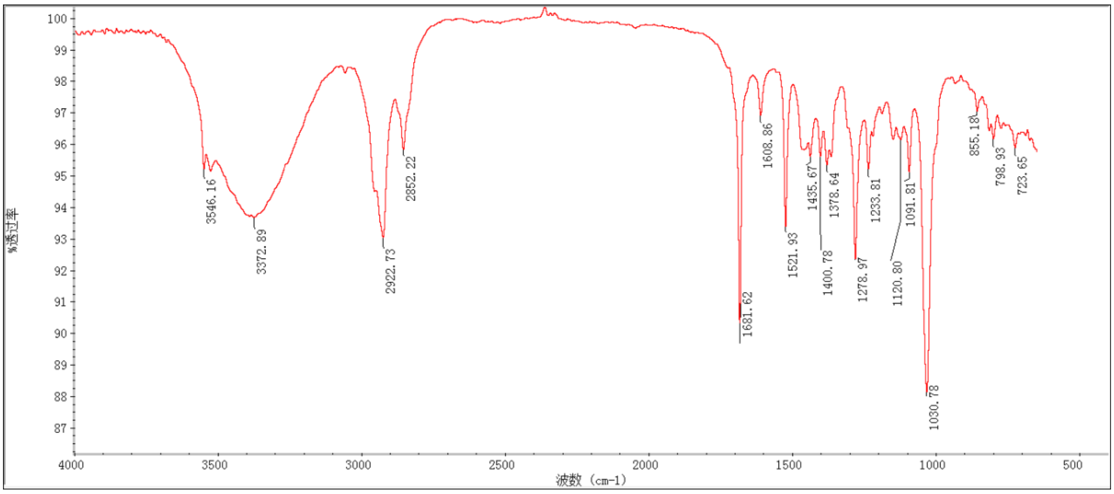

S25. UV spectrum of **3**

Thermo Scientific ~ VISIONpro SOFTWARE V4.41

Operator Name	(None Entered)	Date of Report	2017/11/7
Department	(None Entered)	Time of Report	17:15:11下午
Organization	(None Entered)		
Information	(None Entered)		

Scan Graph

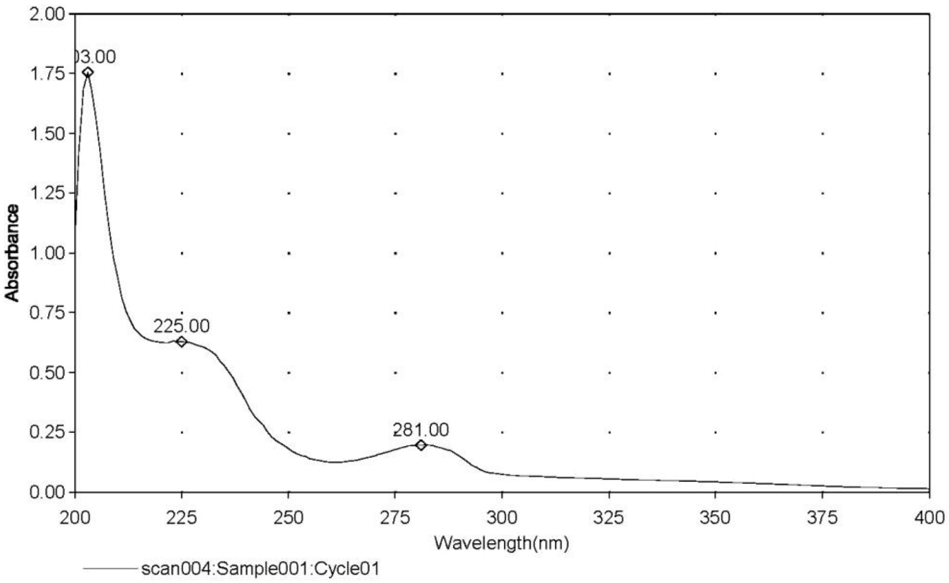

Results Table - LZZ-49.sre,Sample001,Cycle01

nm	A	**Manual Method**
203.00	1.755	Report Values at 3 Wavelengths
225.00	.627	203.00 nm 225.00 nm 281.00 nm
281.00	.196	Sort By Wavelength

S26. ^{1}H NMR spectrum of **4** (500MHz, CDCL$_3$)

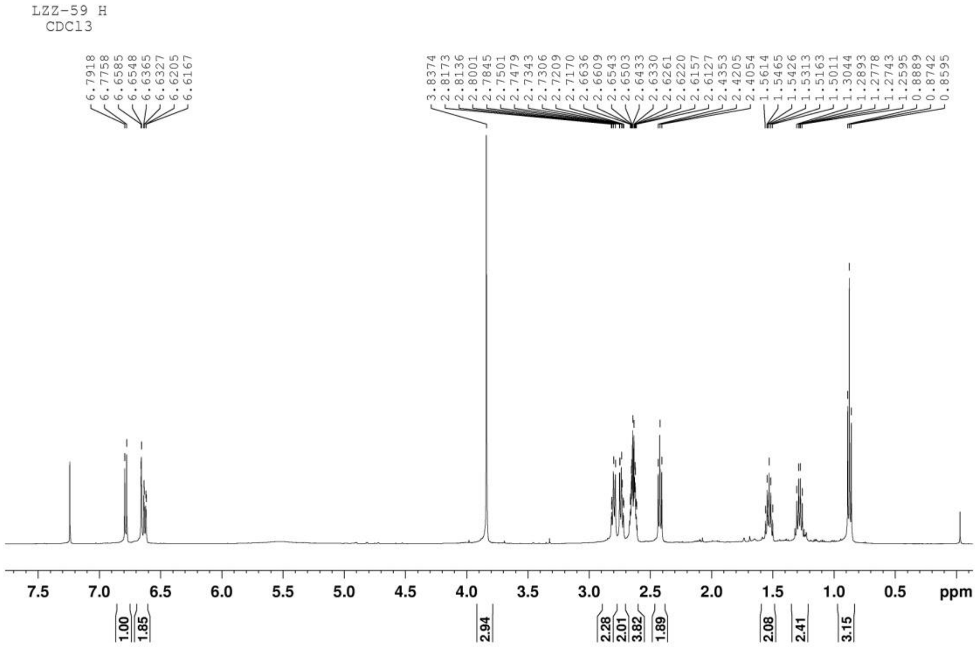

S27. ^{13}C NMR spectrum of **4** (125MHz, CDCL$_3$)

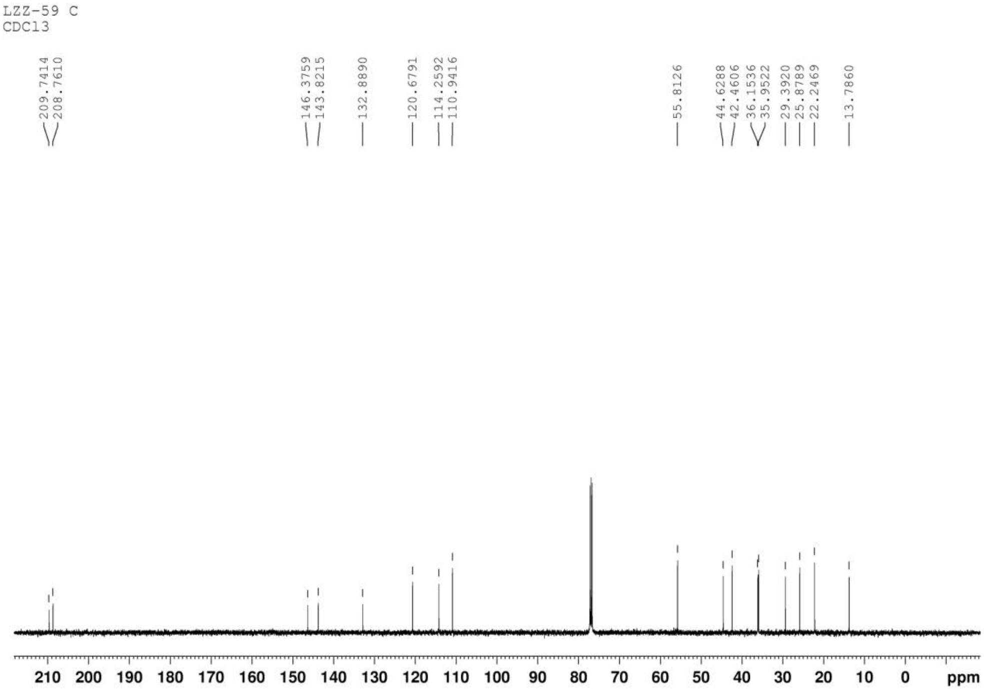

S28. DEPT135 spectrum of **4**

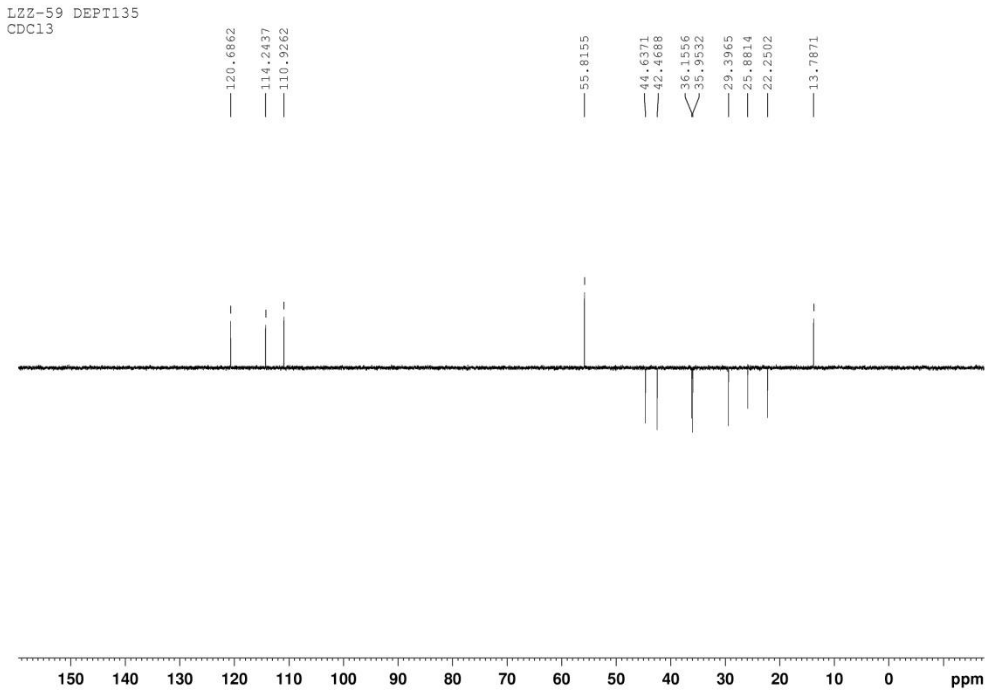

S29. HSQC spectrum of **4**

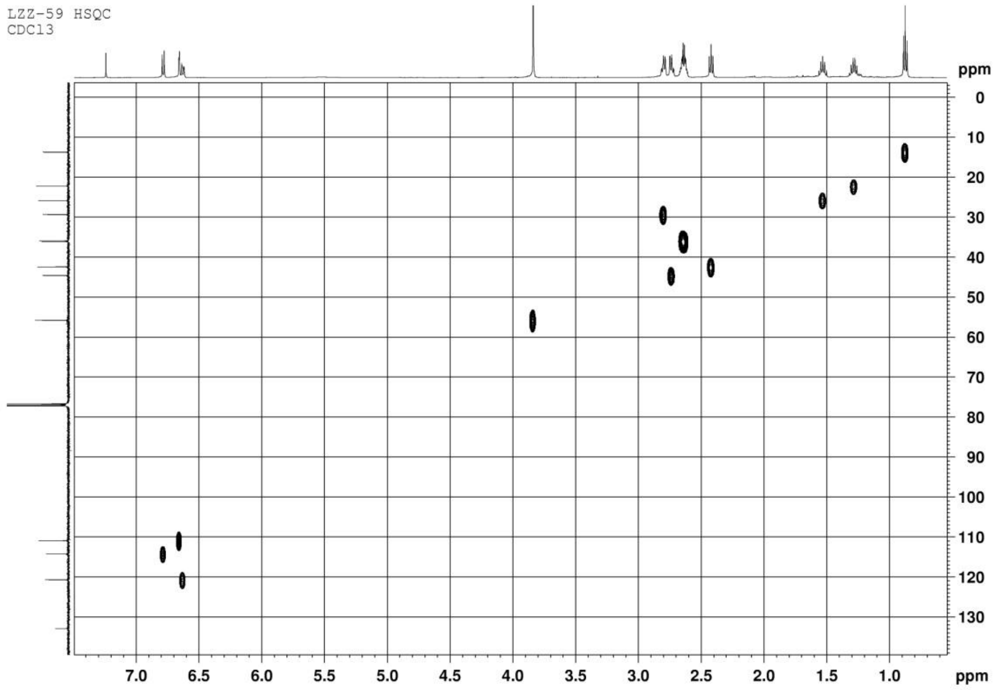

S30. HMBC spectrum of **4**

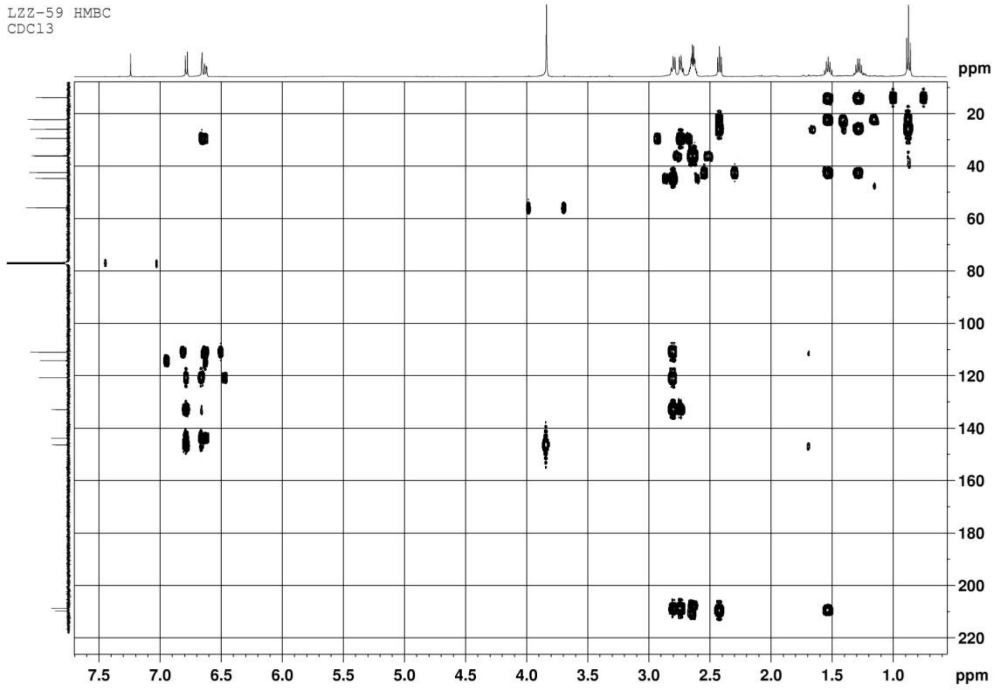

S31. ^{1}H-^{1}H COSY spectrum of **4**

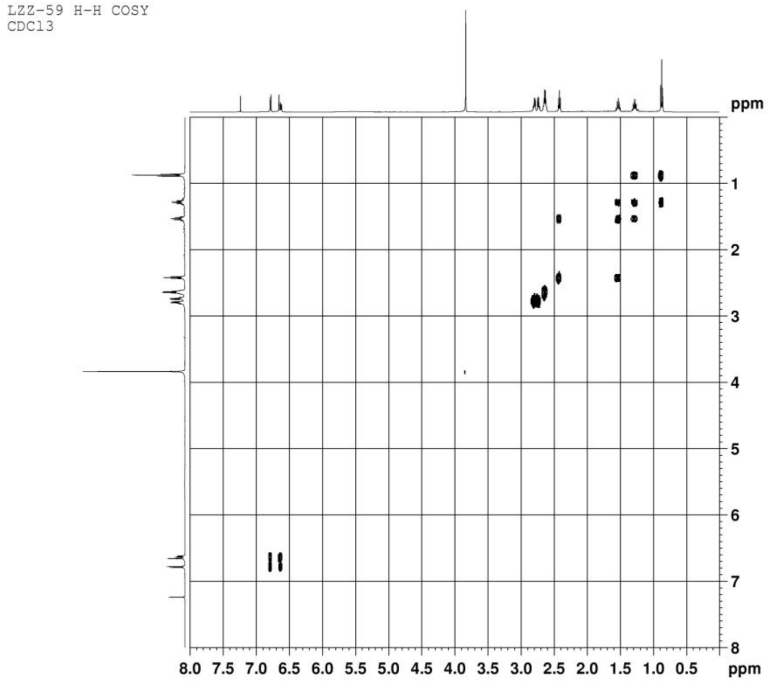

S32. HRESIMS spectrum of **4**

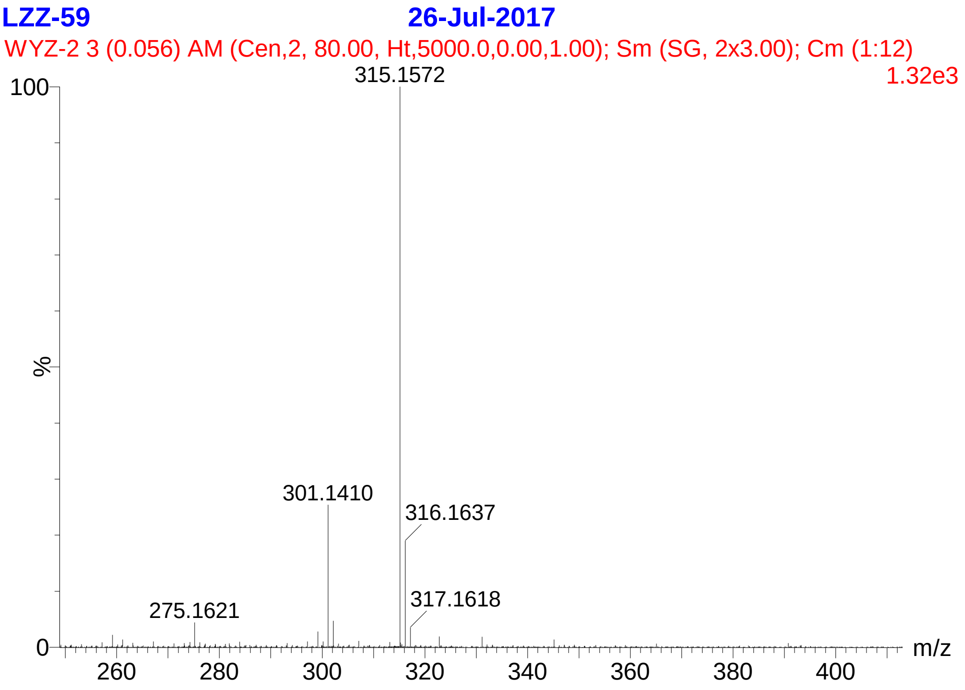

S33. IR spectrum of **4**

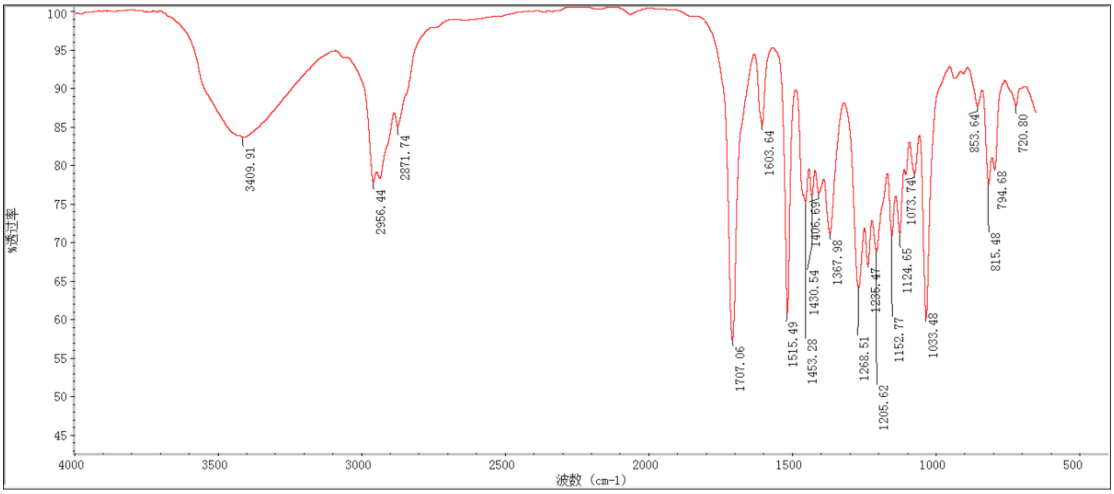

S34. UV spectrum of **4**

Thermo Scientific ~ VISIONpro SOFTWARE V4.41

Operator Name	(None Entered)	Date of Report	2017/11/7
Department	(None Entered)	Time of Report	17:19:40下午
Organization	(None Entered)		
Information	(None Entered)		

Scan Graph

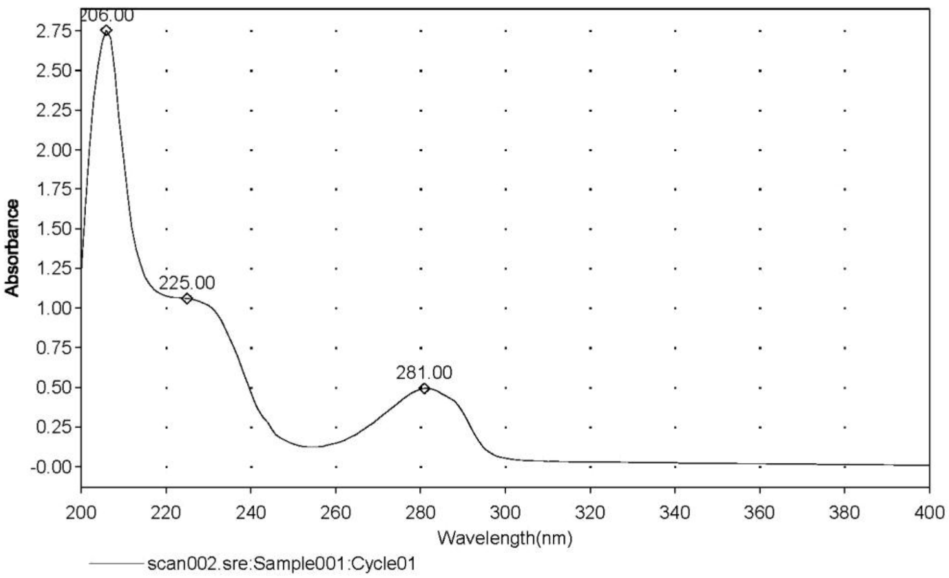

Results Table - scan002.sre,Sample001,Cycle01

nm	A	**Manual Method**
206.00	2.753	Report Values at 3 Wavelengths
225.00	1.058	206.00 nm 225.00 nm 281.00 nm
281.00	.492	Sort By Wavelength

S35. ^{1}H NMR spectrum of **5** (500MHz, CDCL$_3$)

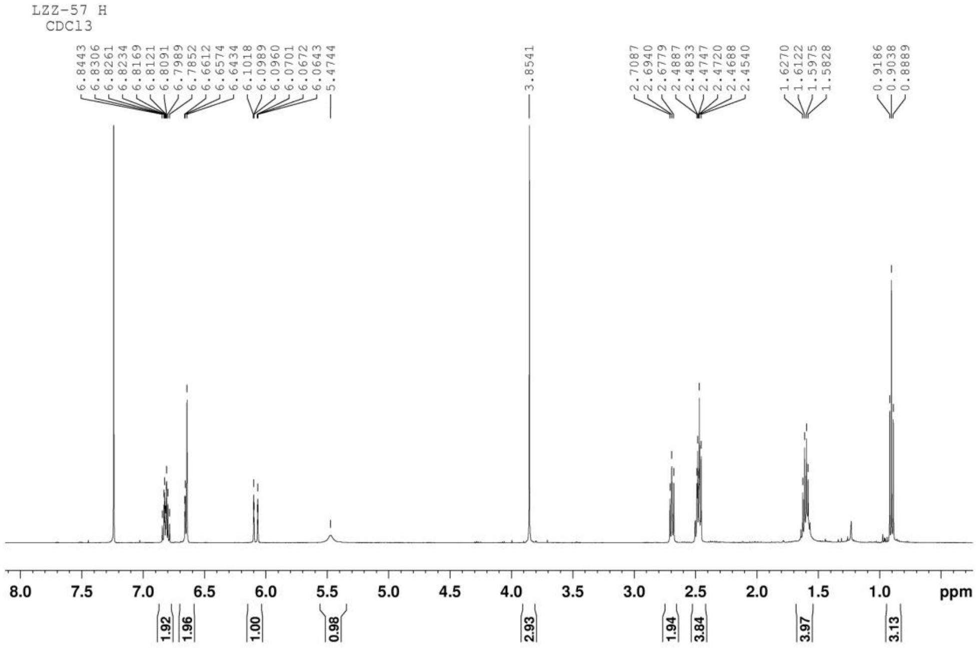

S36. ^{13}C NMR spectrum of **5** (125MHz, CDCL$_3$)

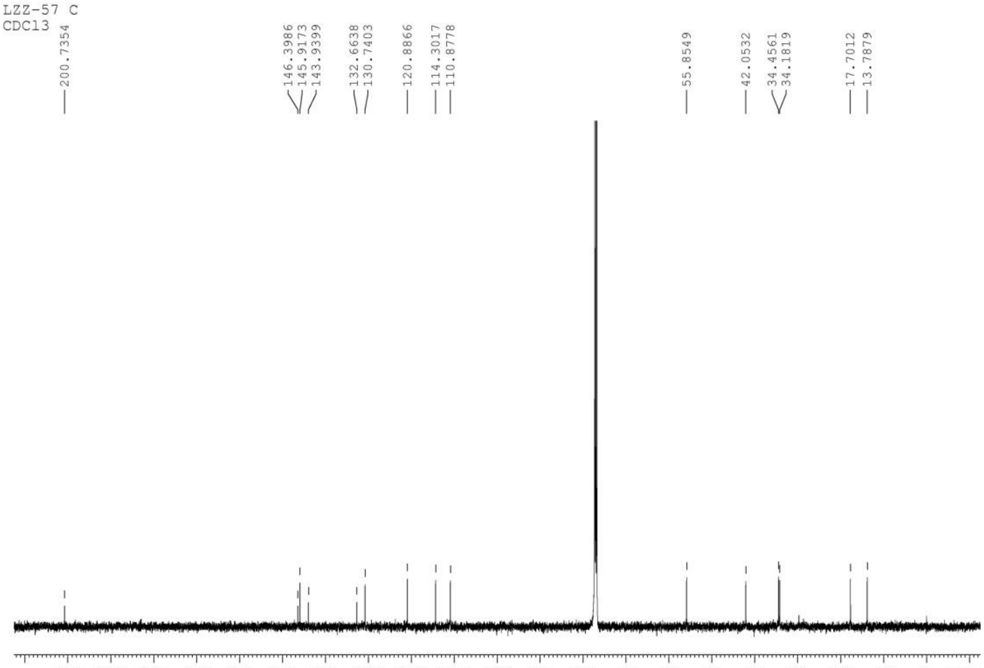

S37. HSQC spectrum of **5**

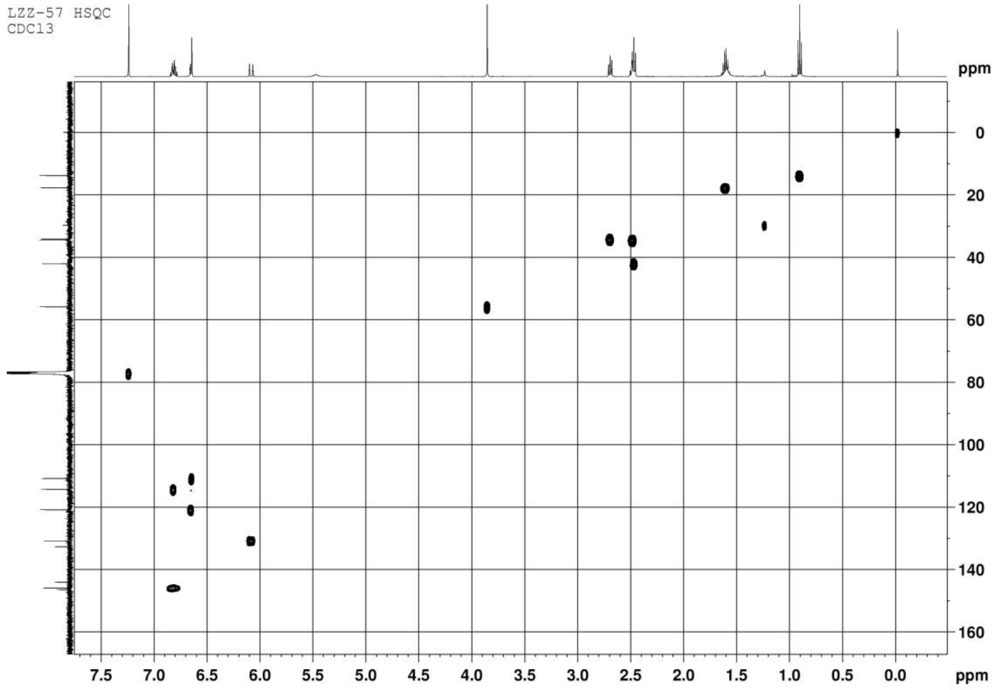

S38. HMBC spectrum of **5**

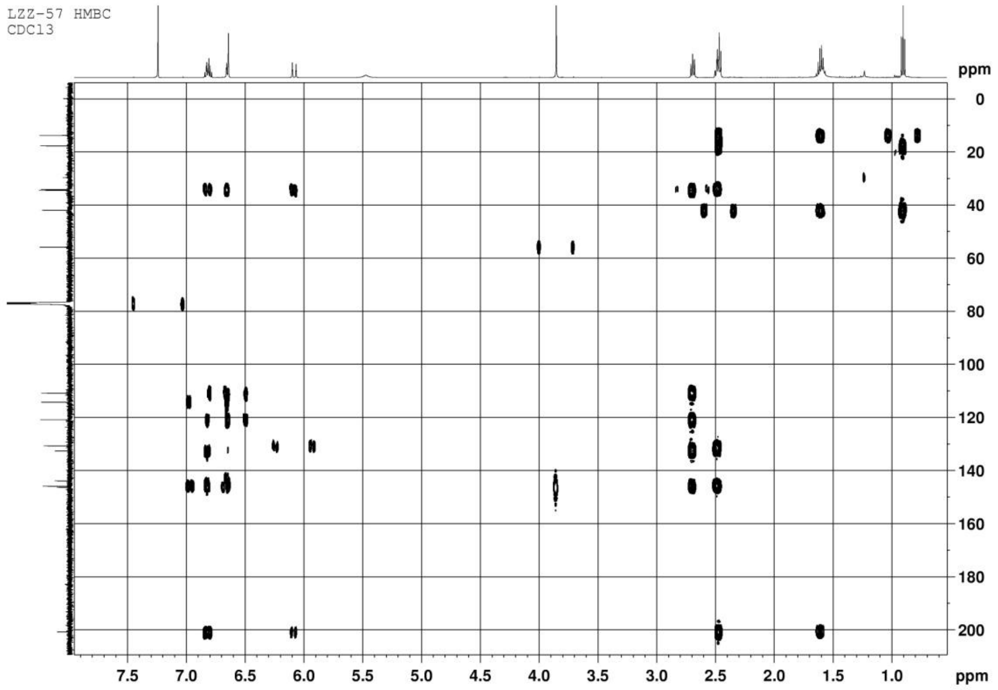

S39. ^{1}H-^{1}H COSY spectrum of **5**

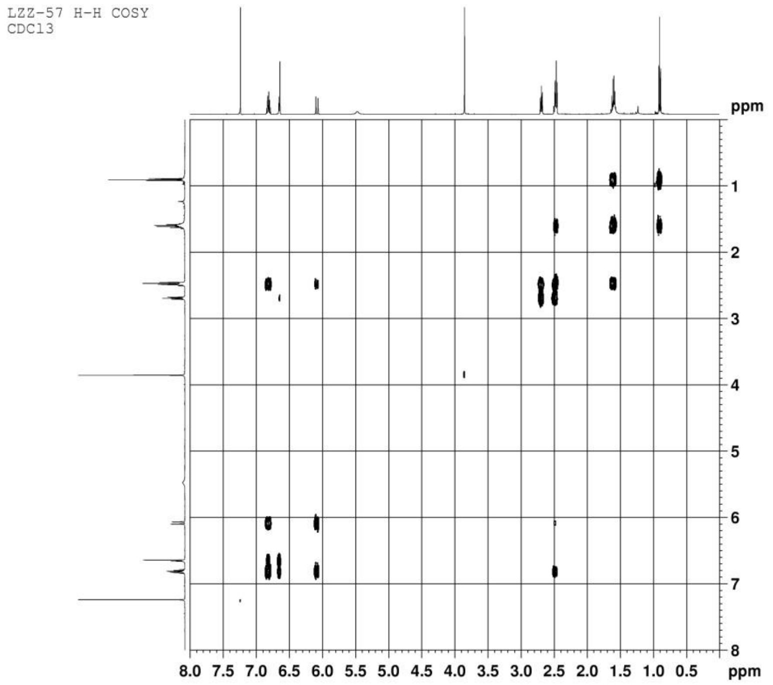

S40. HRESIMS spectrum of **5**

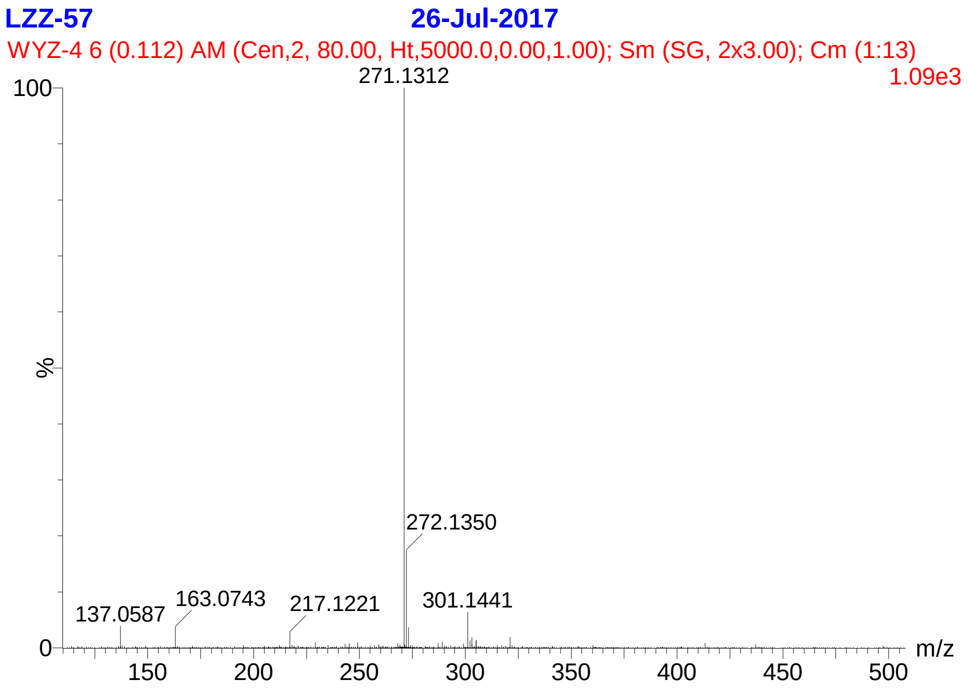

S41. IR spectrum of **5**

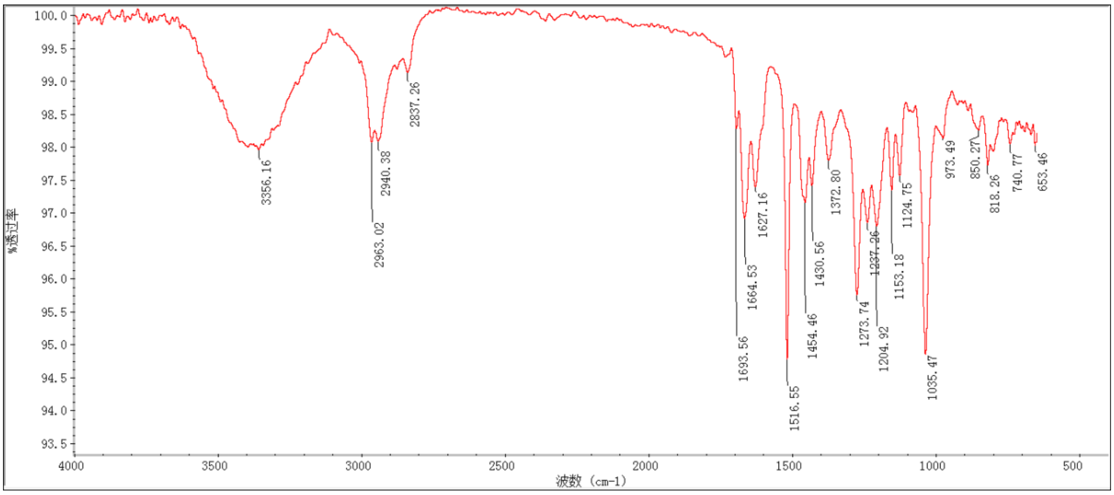

S42. UV spectrum of **5**

Thermo Scientific ~ VISIONpro SOFTWARE V4.41

Operator Name	(None Entered)	Date of Report	2017/11/29
Department	(None Entered)	Time of Report	21:23:34下午
Organization	(None Entered)		
Information	(None Entered)		

Scan Graph

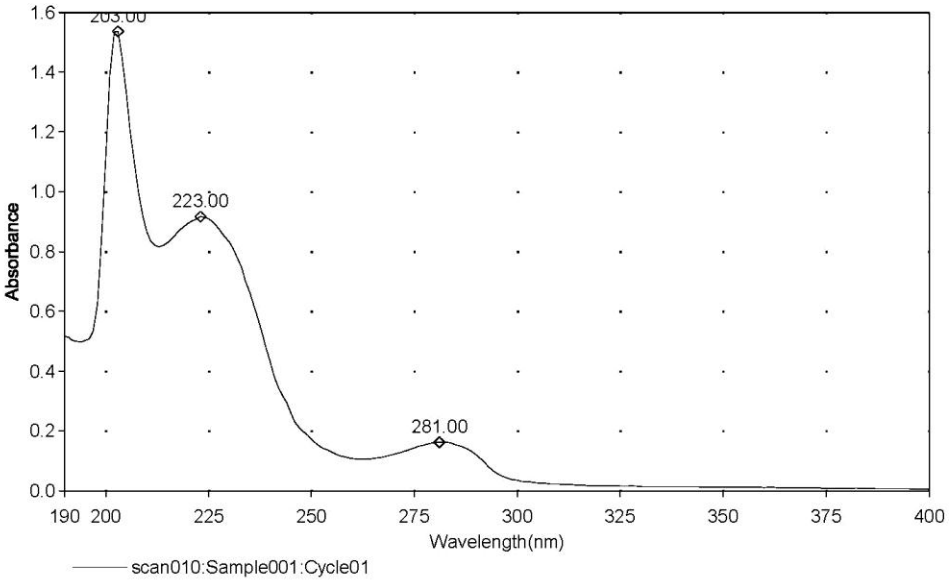

Results Table - scan010,Sample001,Cycle01

nm	A	**Manual Method**
203.00	1.536	Report Values at 3 Wavelengths
223.00	.915	203.00 nm 223.00 nm 281.00 nm
281.00	.162	Sort By Wavelength

S43. ^{1}H NMR spectrum of **6** (500MHz, CDCL$_3$)

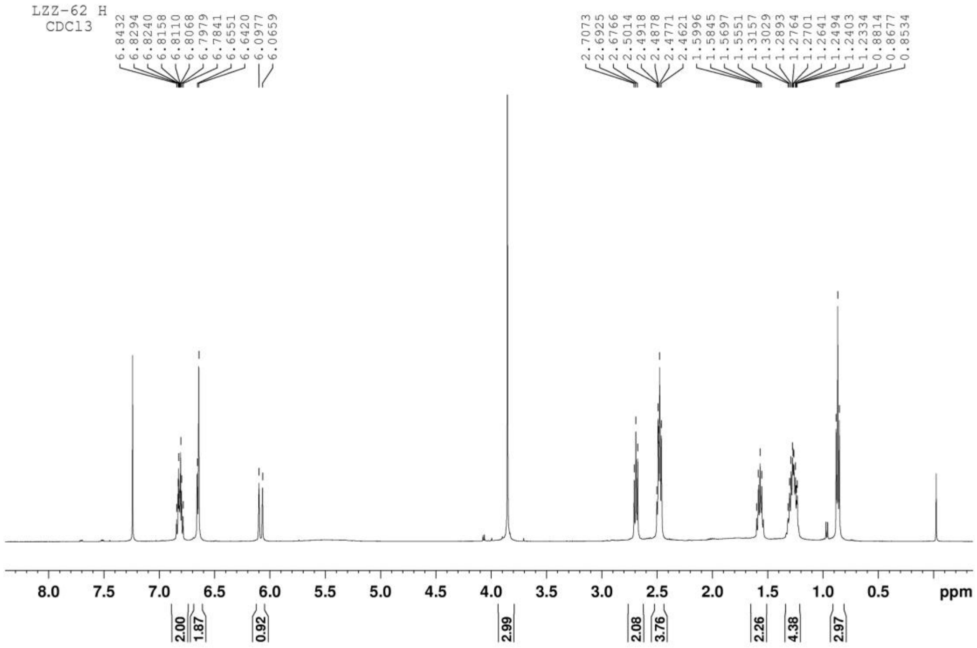

S44. ^{13}C NMR spectrum of **6** (125MHz $_6$, CDCL$_3$)

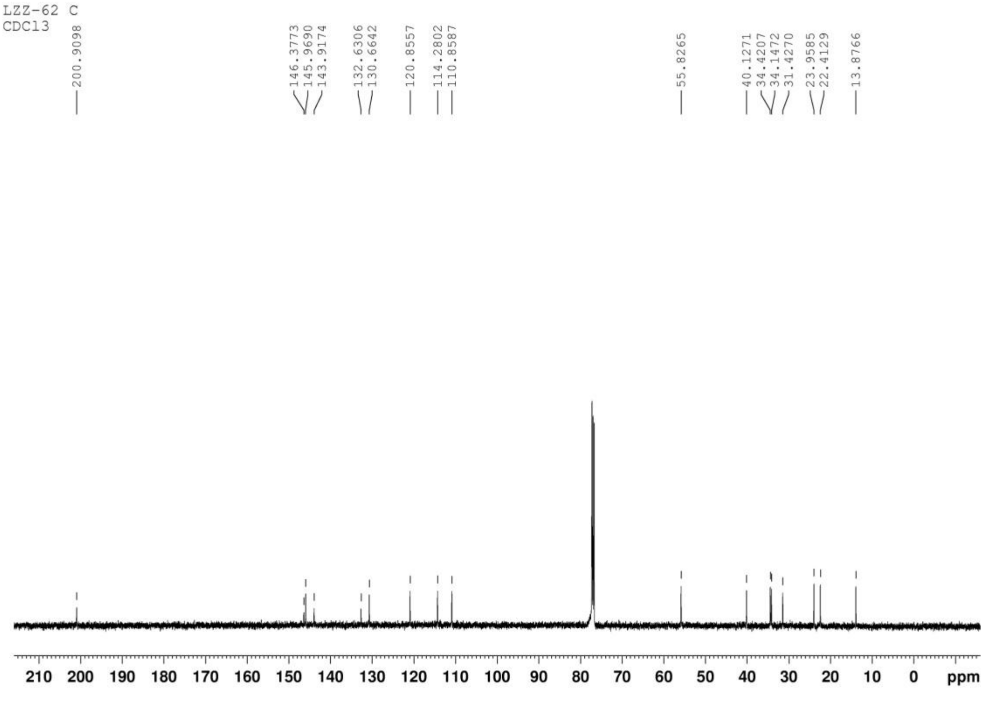

S45. ^{1}H NMR spectrum of **7** (500MHz, $CDCL_3$)

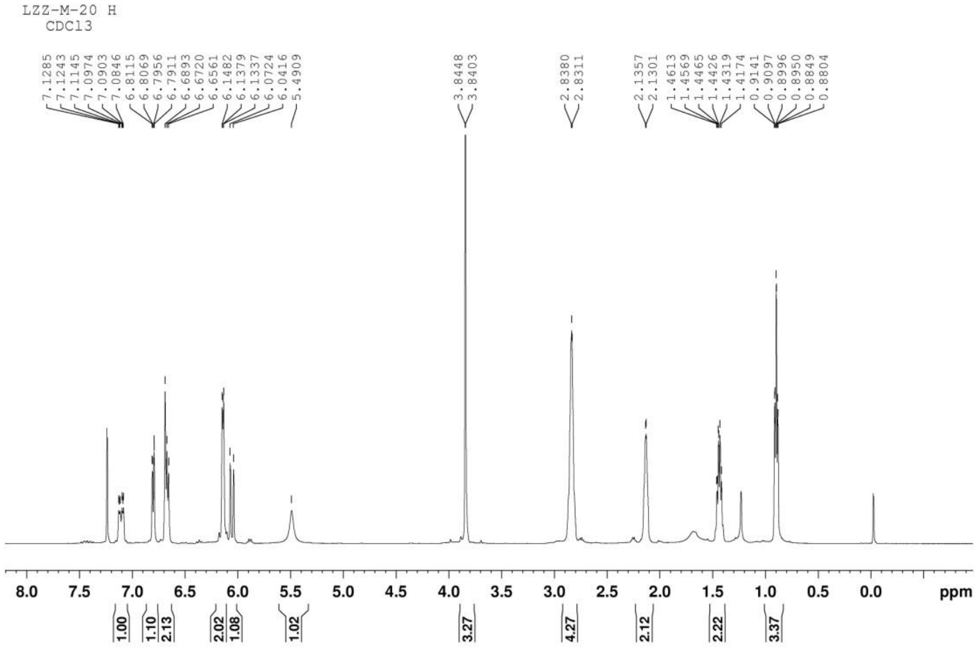

S46. ^{13}C NMR spectrum of **7** (125MHz, $CDCL_3$)

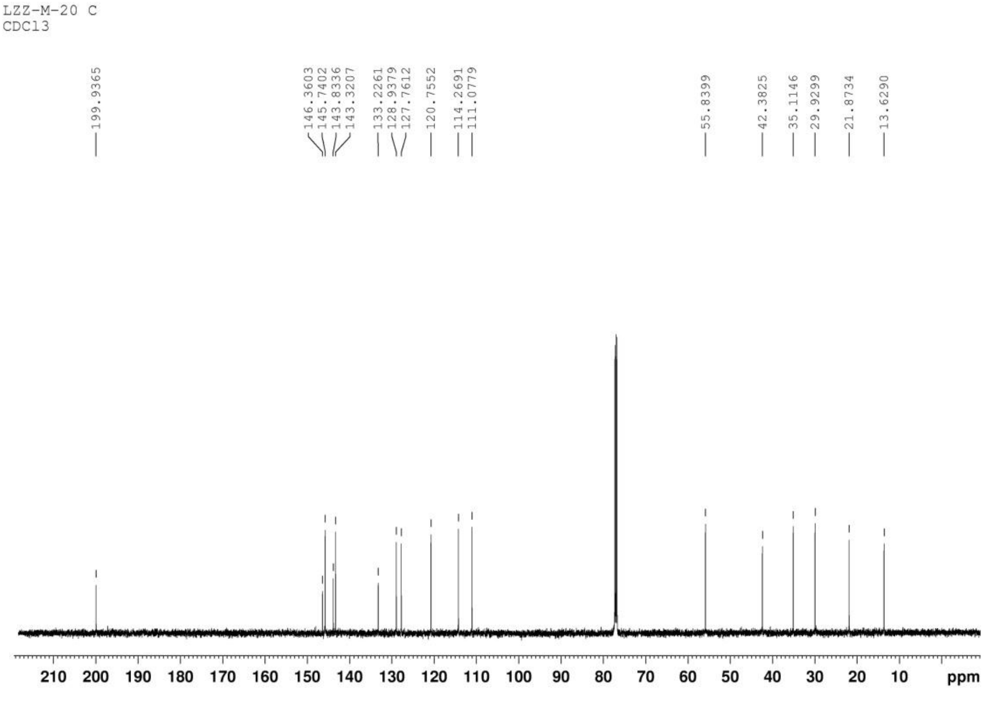

S47. NOESY spectrum of **7**

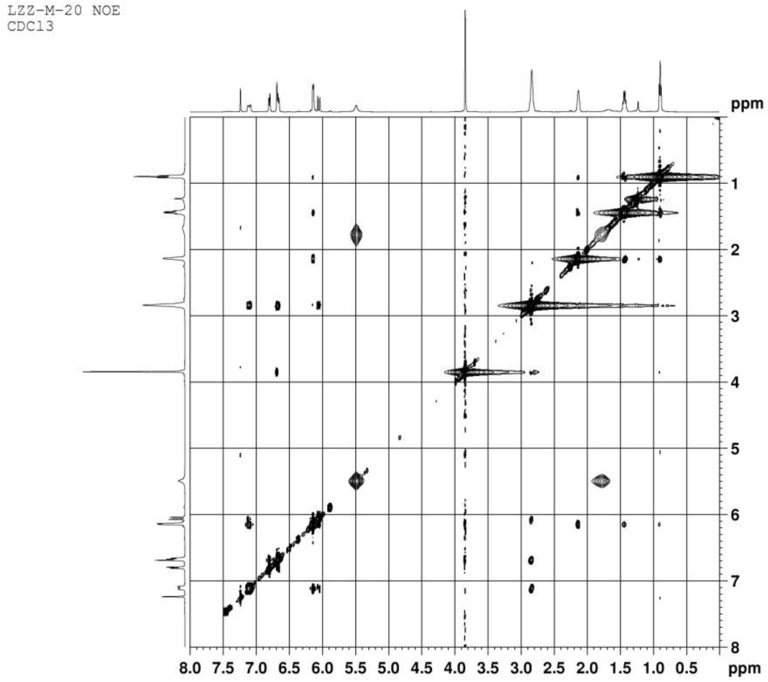

S48. ^{1}H NMR spectrum of **8** (500MHz, CDCL$_3$)

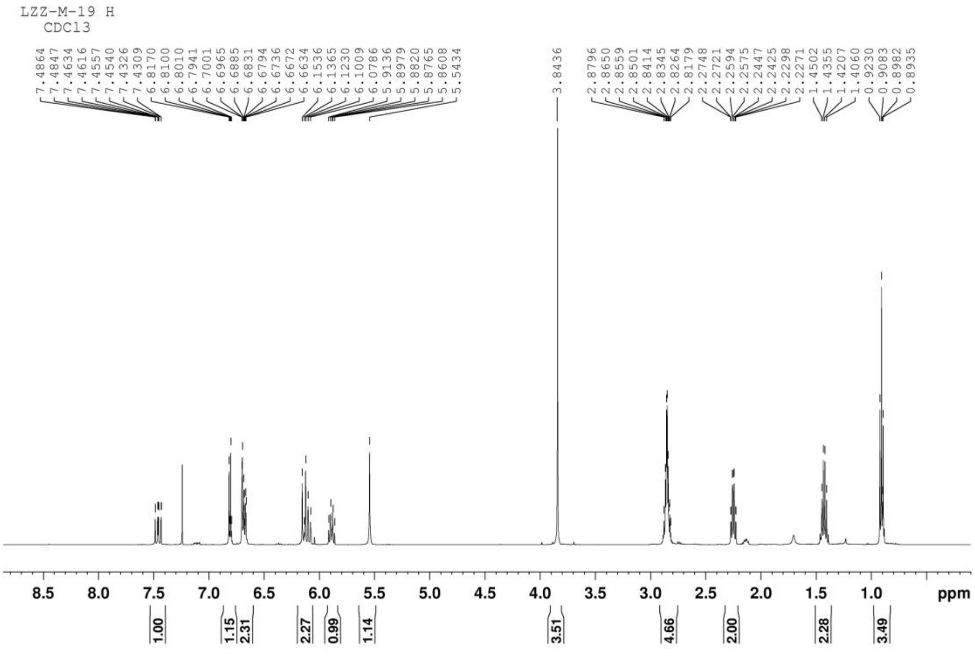

S49. ^{13}C NMR spectrum of **8** (125MHz, CDCL$_3$)

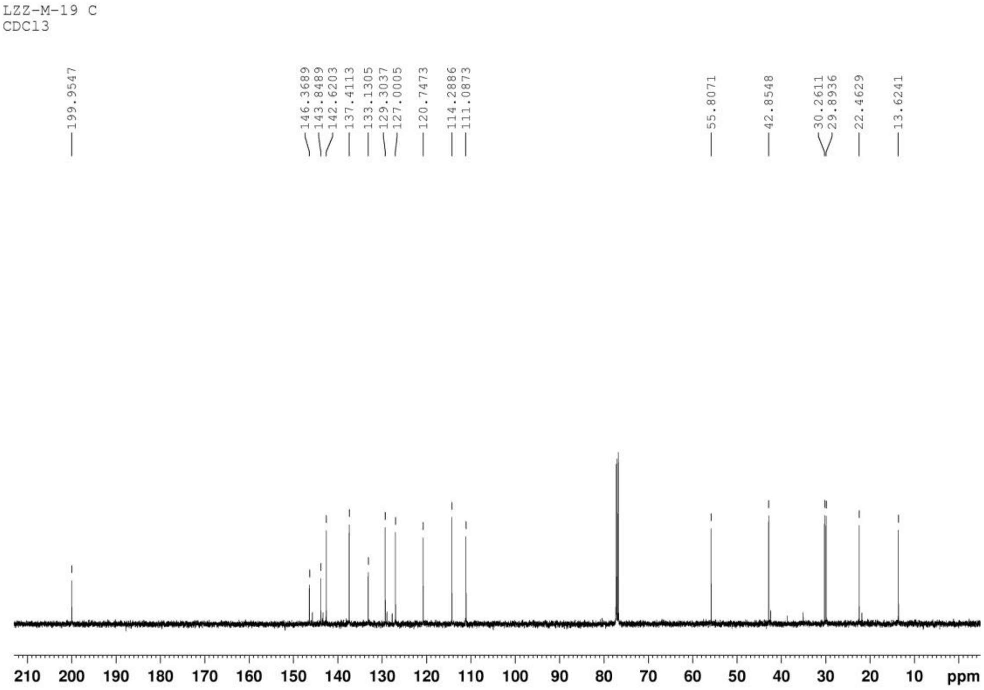

S50. HSQC spectrum of **8**

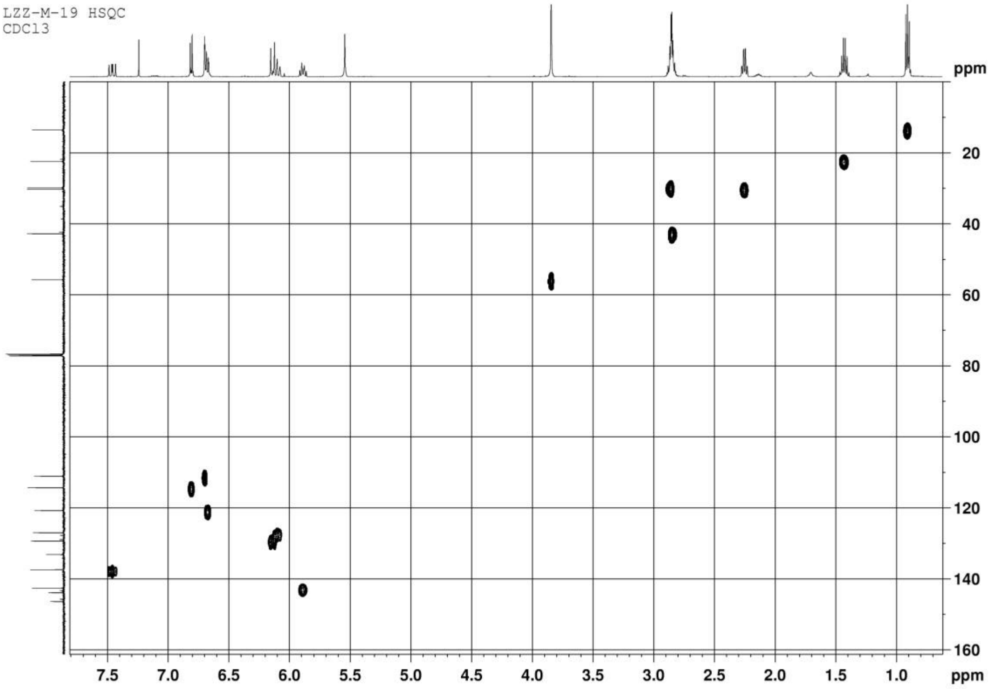

S51. HMBC spectrum of **8**

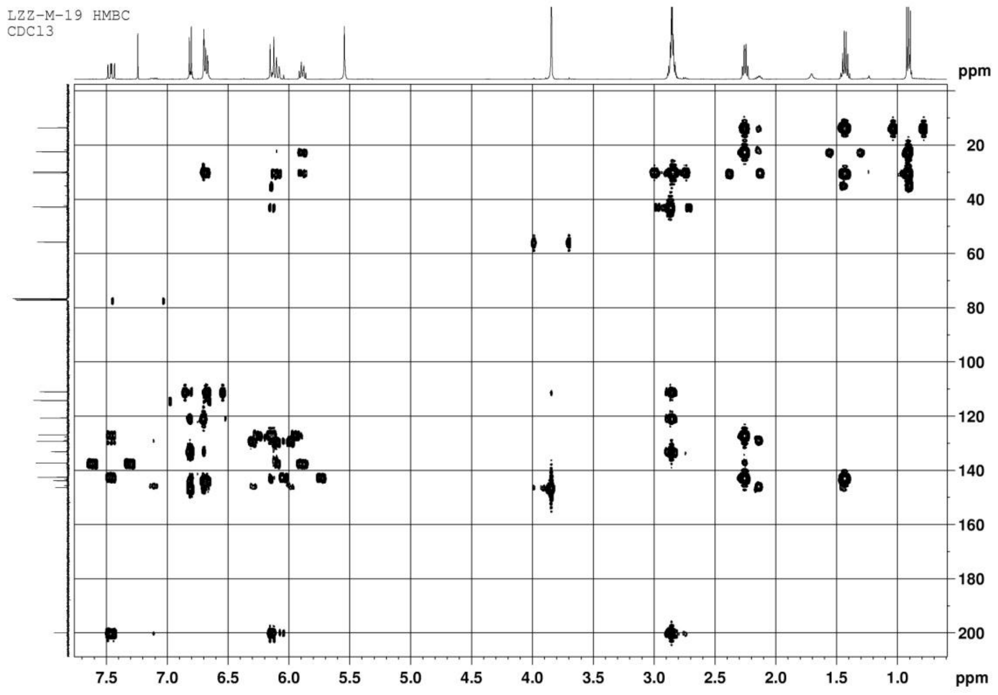

S52. NOESY spectrum of **8**

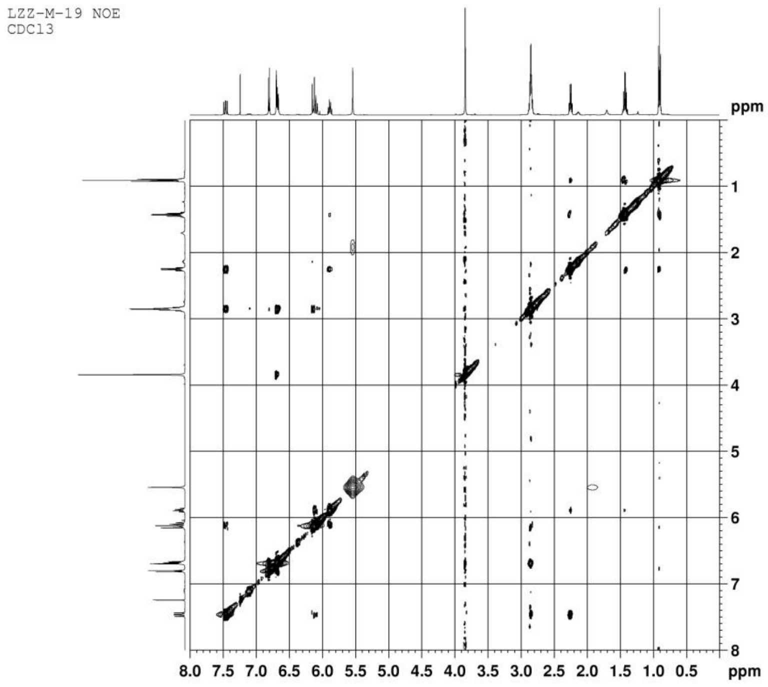

S53. HRESIMS spectrum of **8**

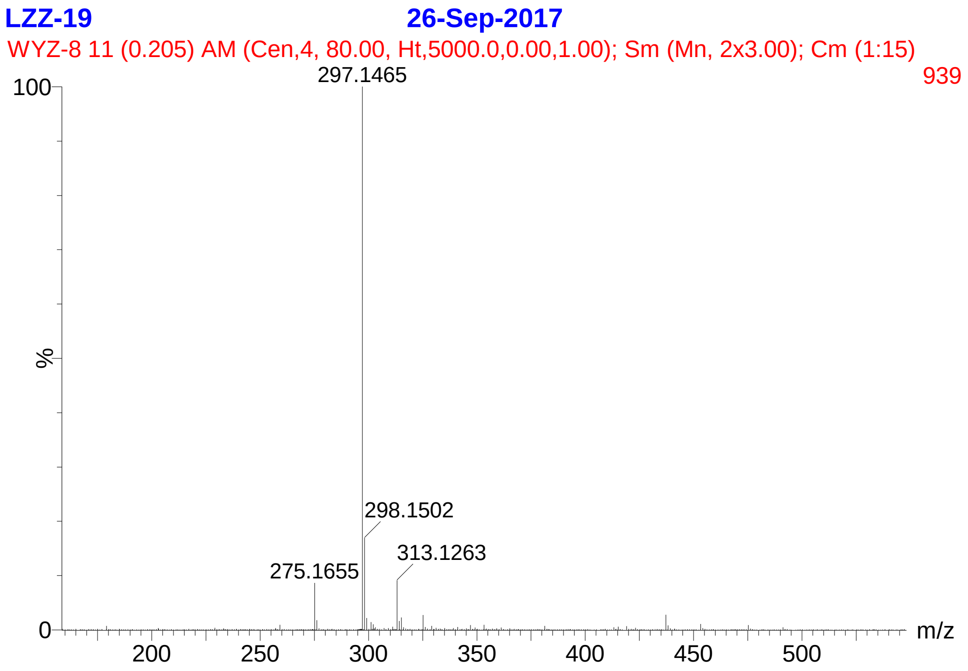

S54. IR spectrum of **8**

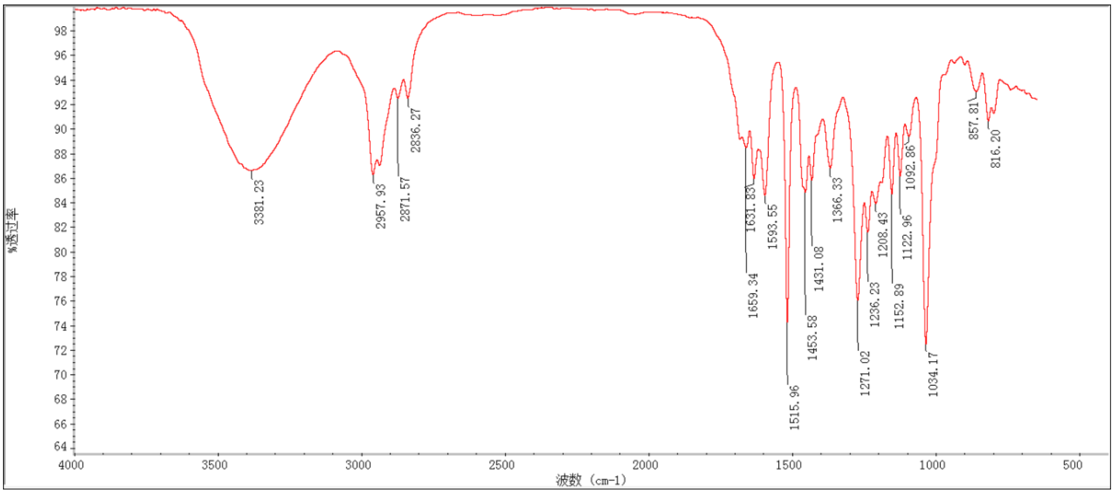

S55. UV spectrum of **8**

Thermo Scientific ~ VISIONpro SOFTWARE V4.41

Operator Name	(None Entered)	Date of Report	2017/11/7
Department	(None Entered)	Time of Report	17:32:33下午
Organization	(None Entered)		
Information	(None Entered)		

Scan Graph

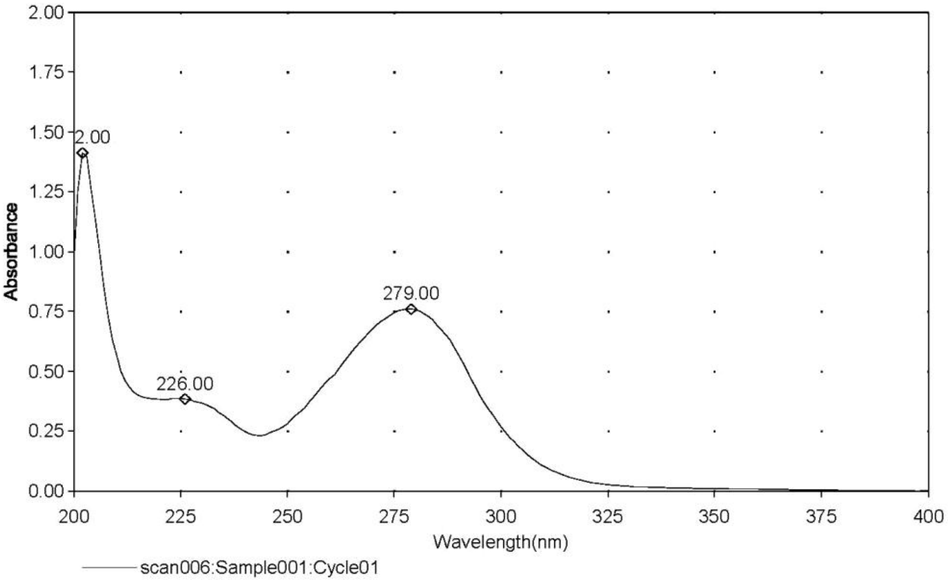

Results Table - LZZ-M-19.xps.sre,Sample001,Cycle01

nm	A	**Manual Method**
202.00	1.412	Report Values at 3 Wavelengths
226.00	.383	202.00 nm 226.00 nm 279.00 nm
279.00	.759	Sort By Wavelength

S56. ^{1}H NMR spectrum of **9** (500MHz, CDCL$_3$)

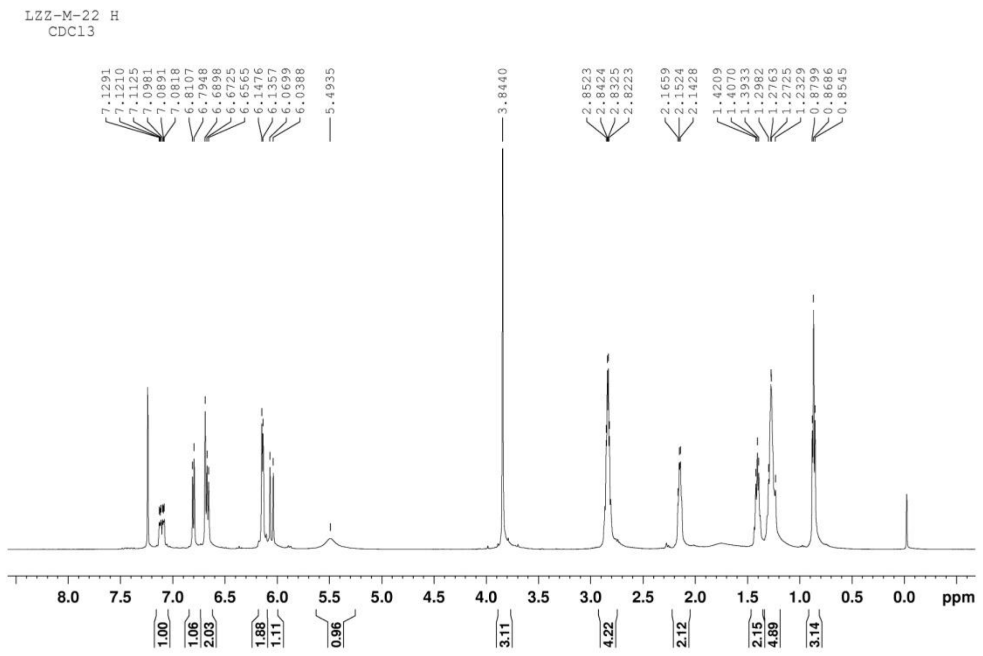

S57. ^{13}C NMR spectrum of **9** (125MHz, CDCL$_3$)

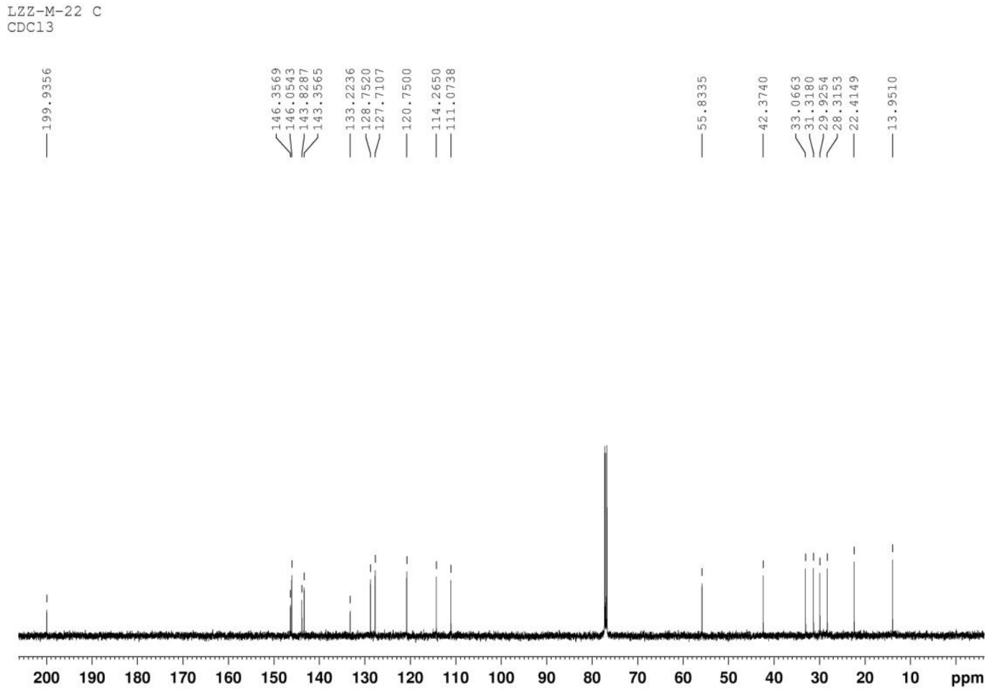

S58. HSQC spectrum of **9**

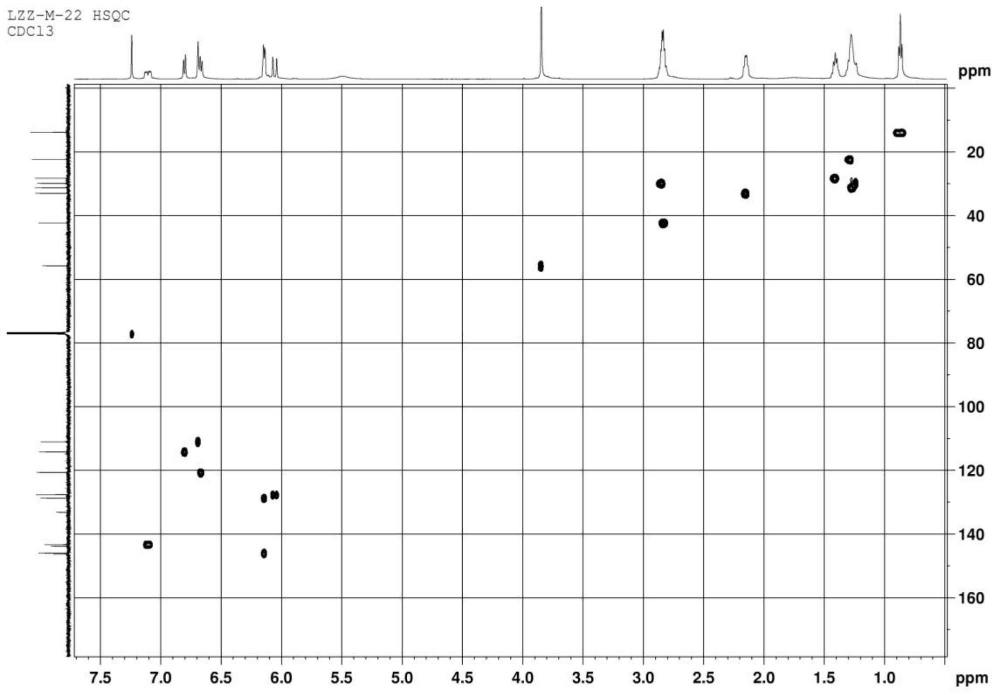

S59. HMBC spectrum of **9**

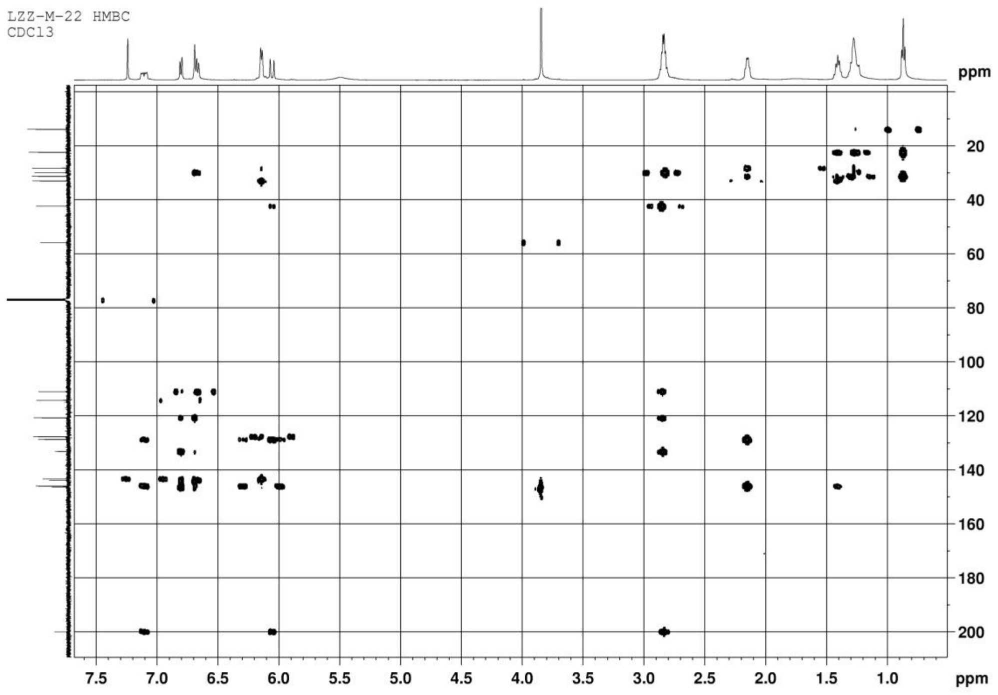

S60. NOESY spectrum of **9**

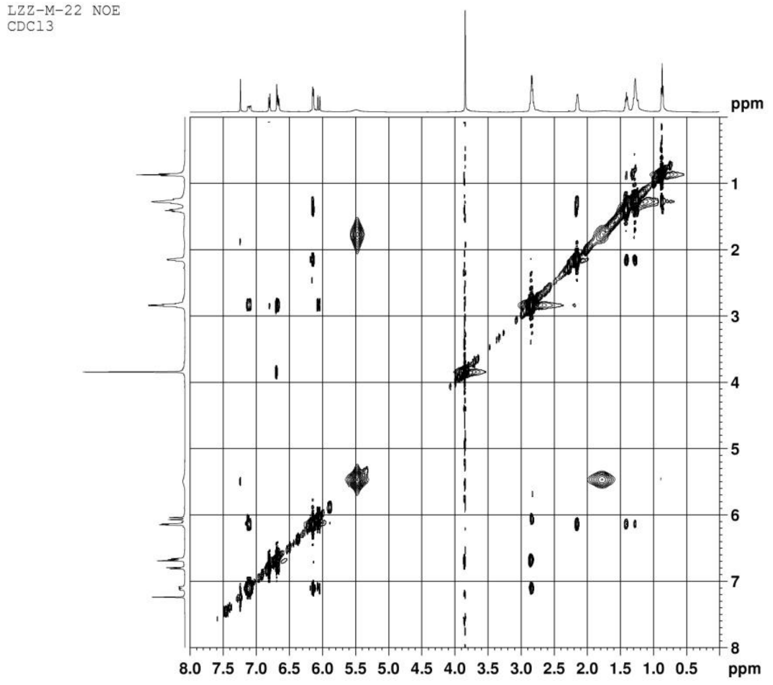

S61. HRESIMS spectrum of **9**

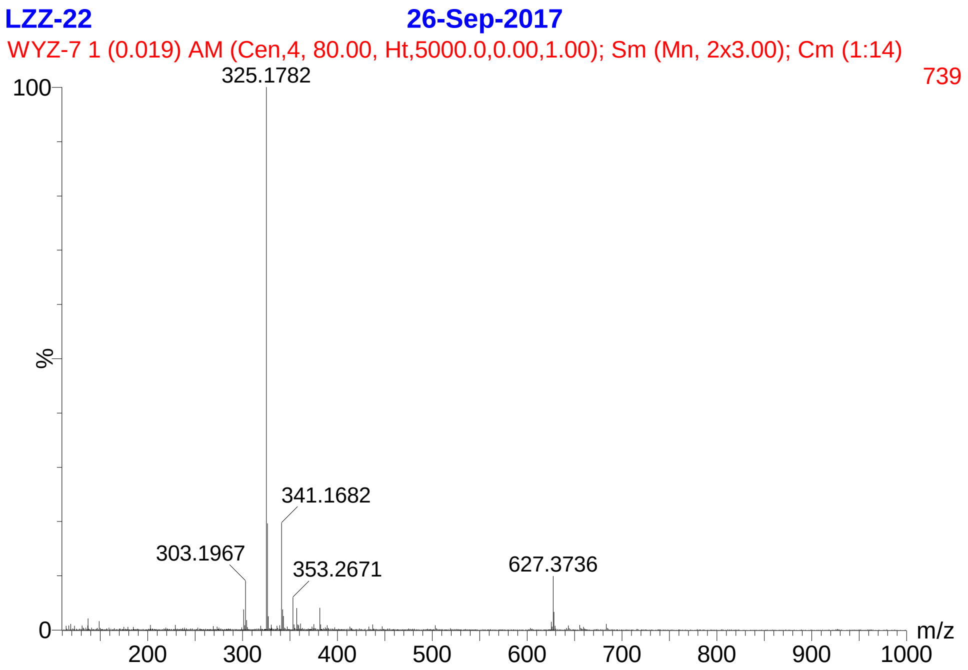

S62. IR spectrum of **9**

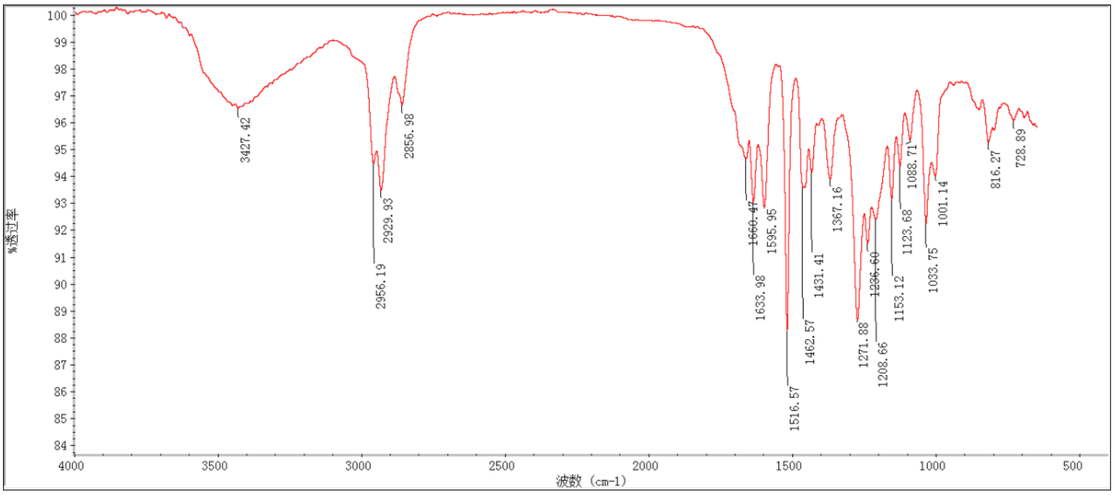

S63. UV spectrum of **9**

Thermo Scientific ~ VISIONpro SOFTWARE V4.41

Operator Name	(None Entered)	Date of Report	2017/11/7
Department	(None Entered)	Time of Report	17:42:20下午
Organization	(None Entered)		
Information	(None Entered)		

Scan Graph

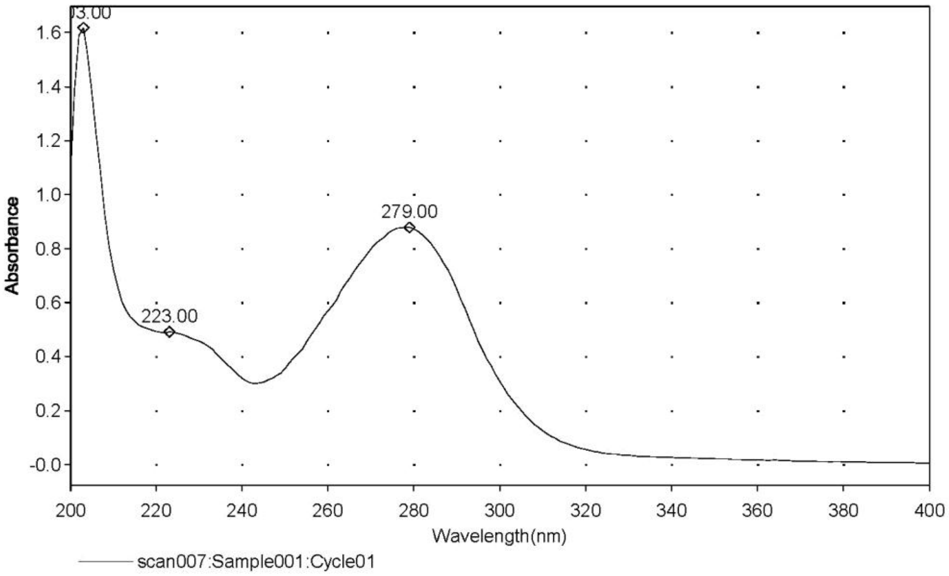

Results Table - scan007,Sample001,Cycle01

nm	A	**Manual Method**
203.00	1.618	Report Values at 3 Wavelengths
223.00	.492	203.00 nm 223.00 nm 279.00 nm
279.00	.878	Sort By Wavelength

S64. ^{1}H NMR spectrum of **10** (500MHz, $CDCL_3$)

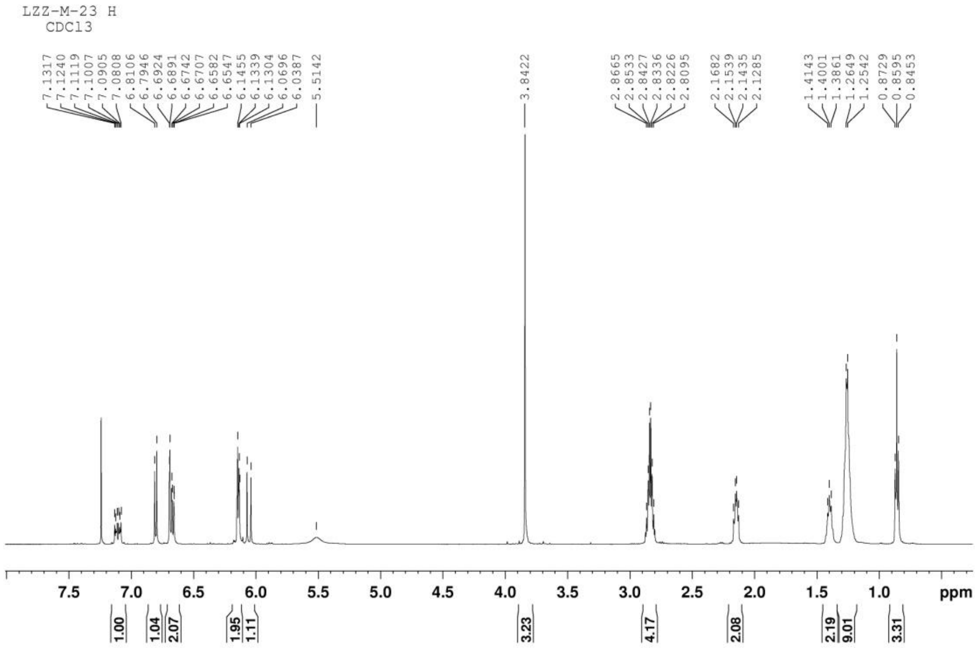

S65. ^{13}C NMR spectrum of **10** (125MHz, $CDCL_3$)

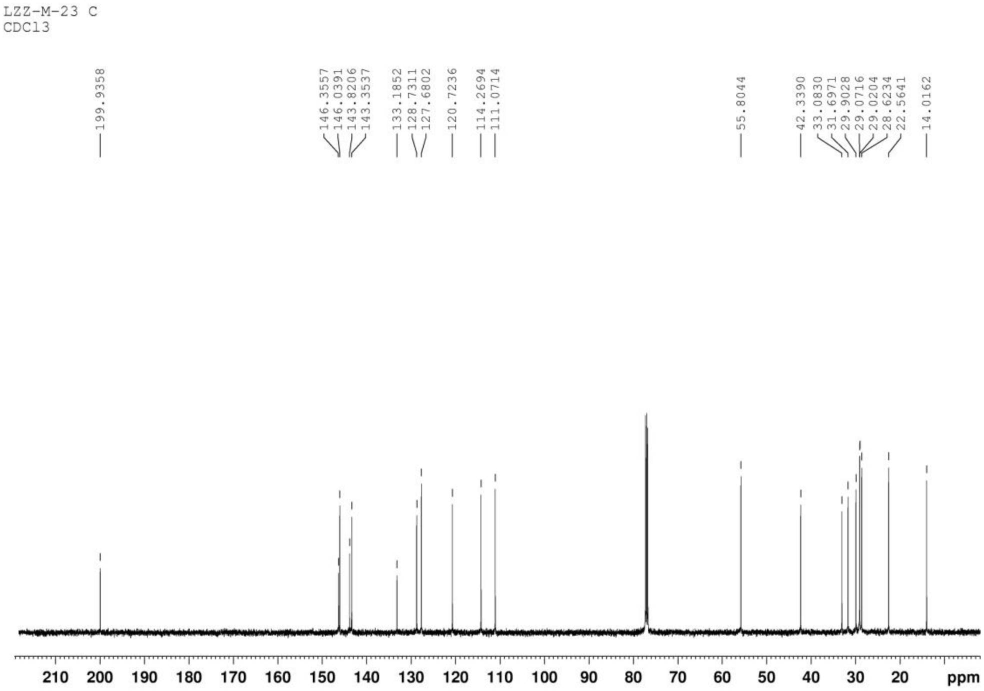

S66. HSQC spectrum of **10**

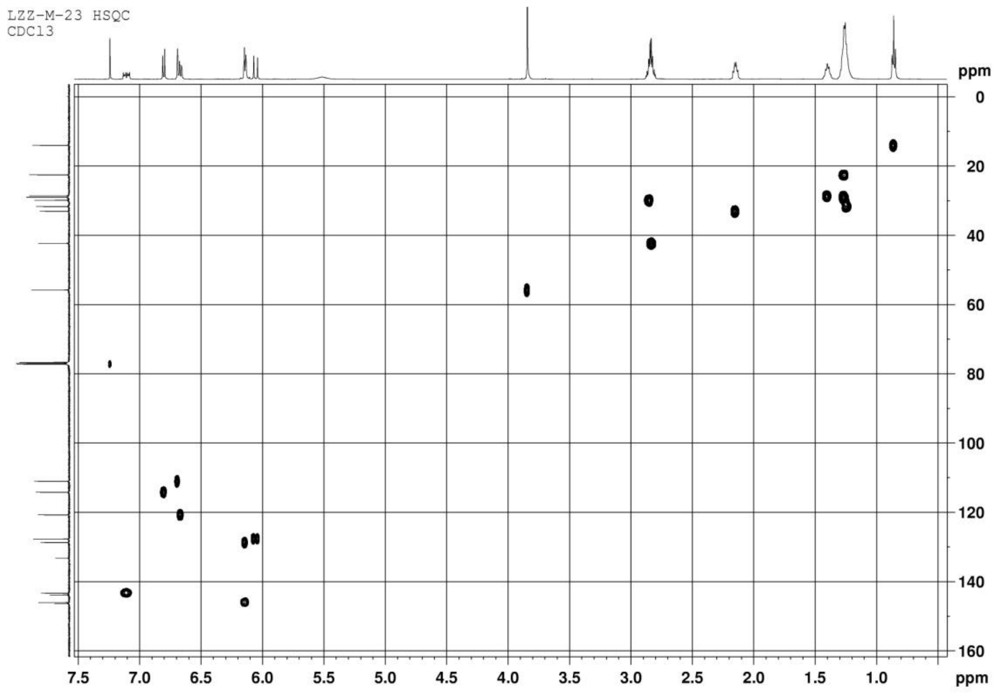

S67. HMBC spectrum of **10**

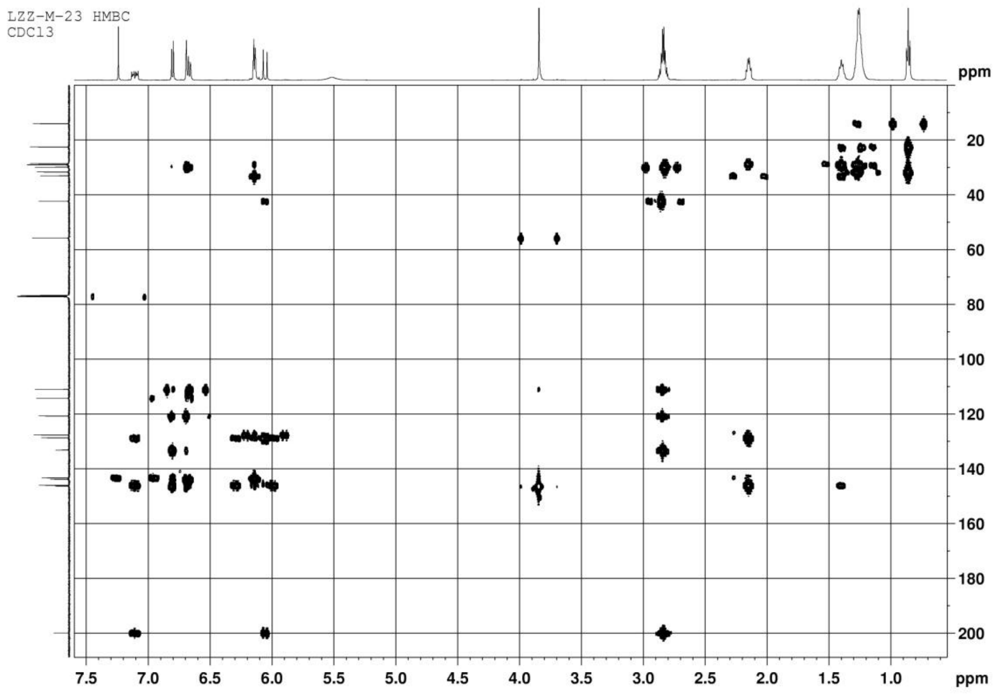

S68. NOESY spectrum of **10**

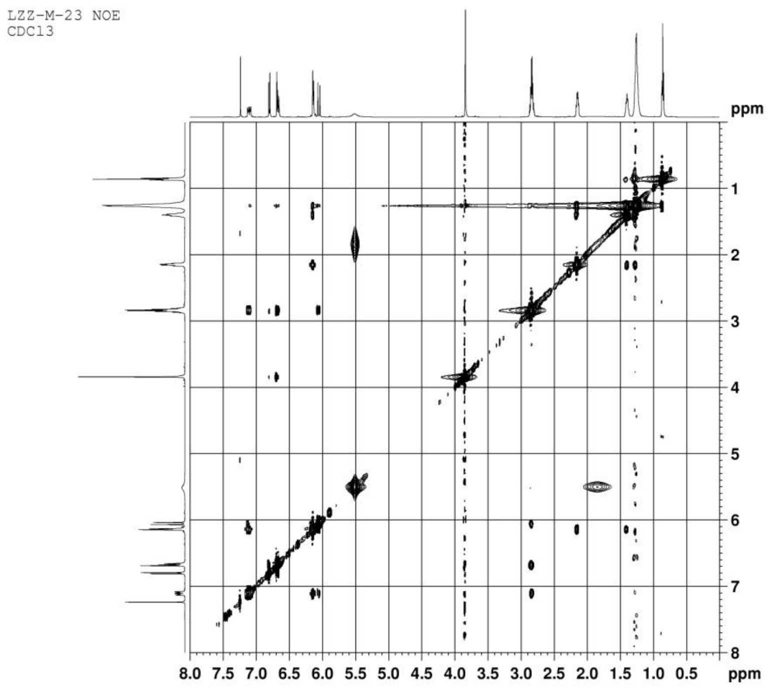

S69. HRESIMS spectrum of **10**

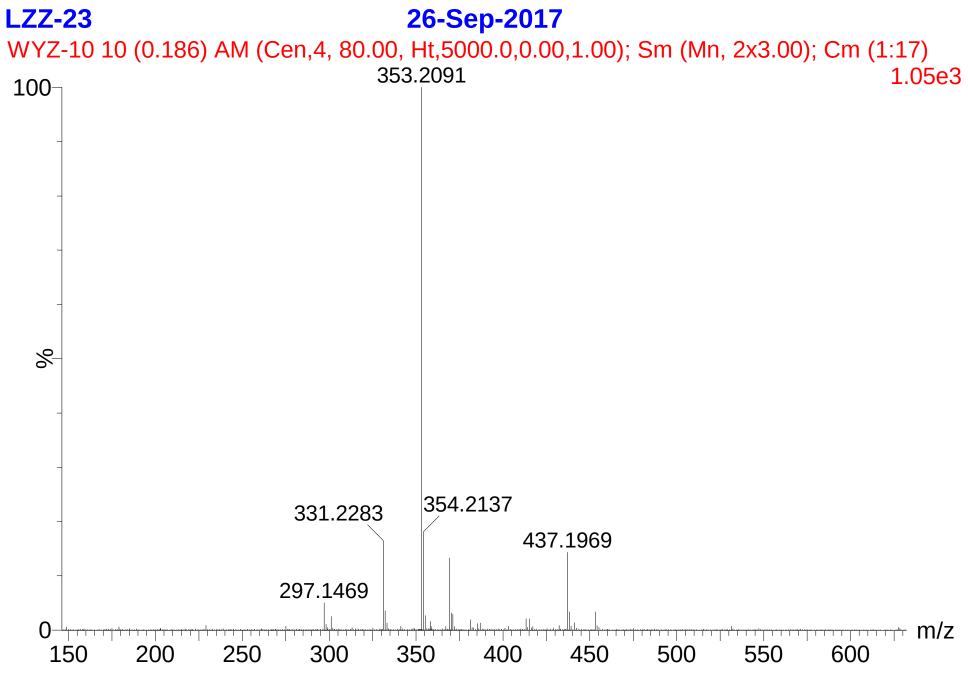

S70. IR spectrum of **10**

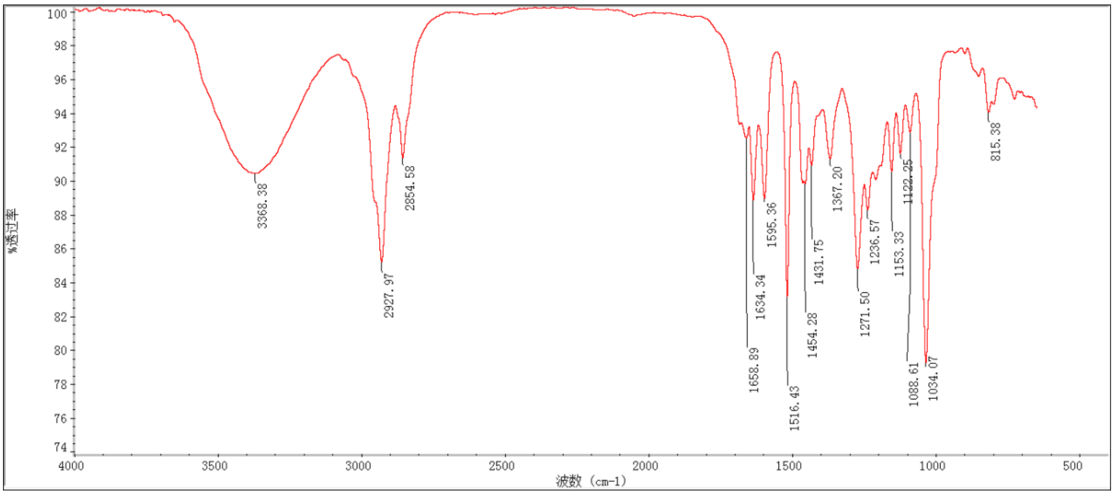

S71. UV spectrum of **10**

Thermo Scientific ~ VISIONpro SOFTWARE V4.41

Operator Name	(None Entered)	Date of Report	2017/11/7
Department	(None Entered)	Time of Report	17:48:59下午
Organization	(None Entered)		
Information	(None Entered)		

Scan Graph

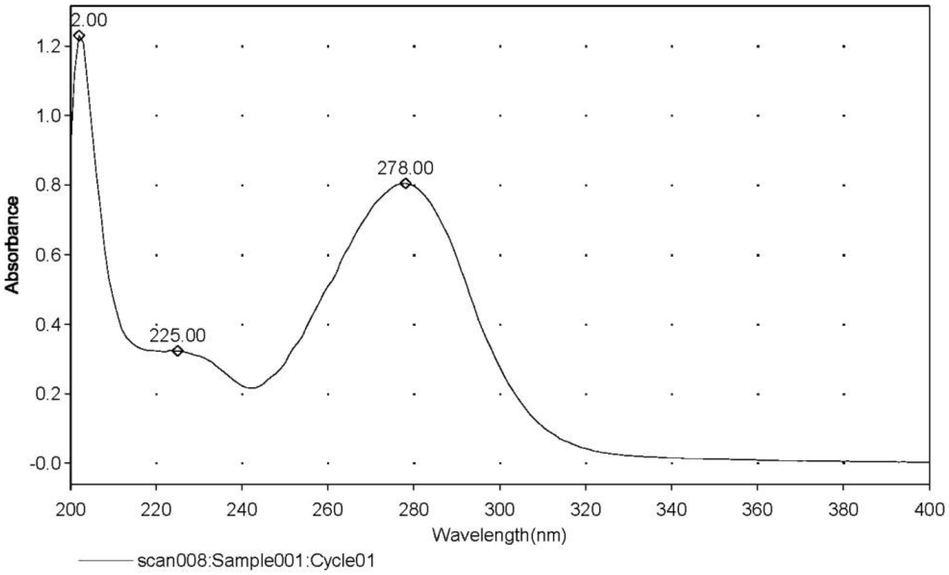

Results Table - scan008,Sample001,Cycle01

nm	A	**Manual Method**
202.00	1.229	Report Values at 3 Wavelengths
225.00	.322	202.00 nm 225.00 nm 278.00 nm
278.00	.804	Sort By Wavelength

S72. ^{1}H NMR spectrum of **11** (500MHz, $CDCL_3$)

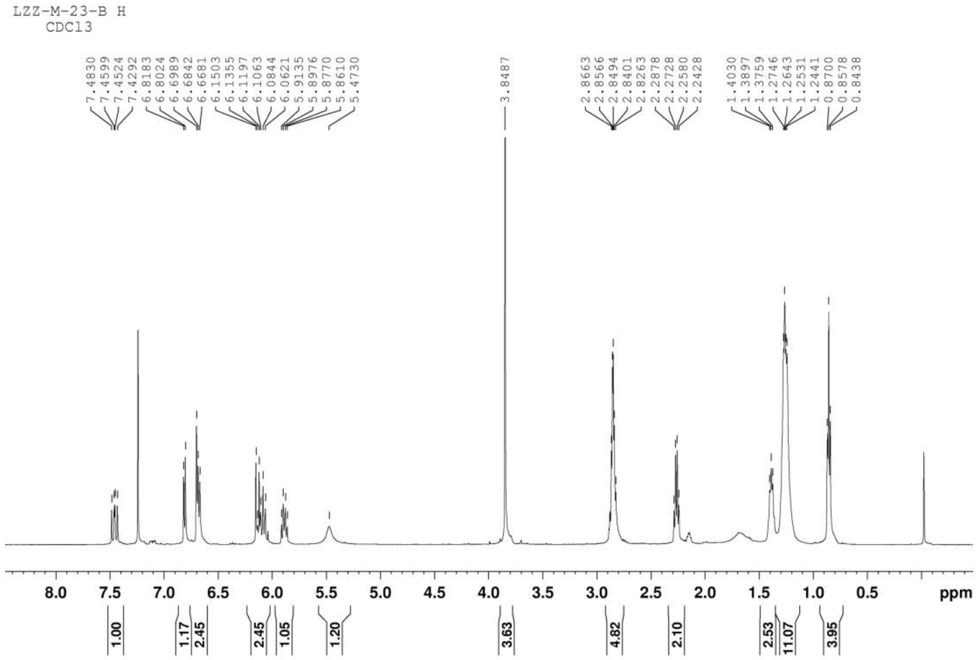

S73. ^{13}C NMR spectrum of **11** (125MHz, $CDCL_3$)

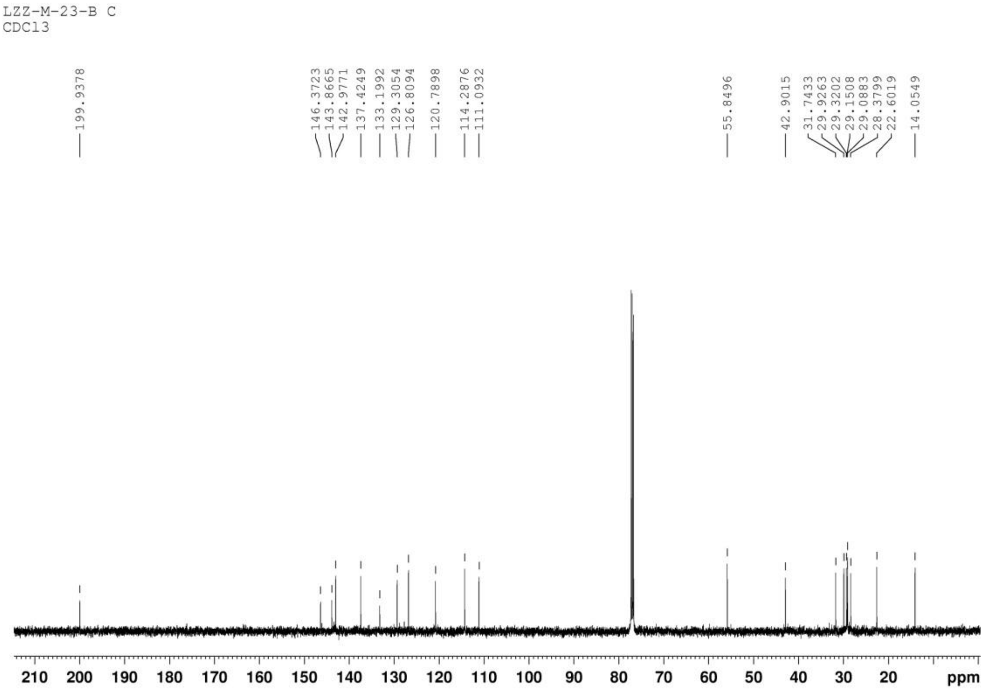

S74. HSQC spectrum of **11**

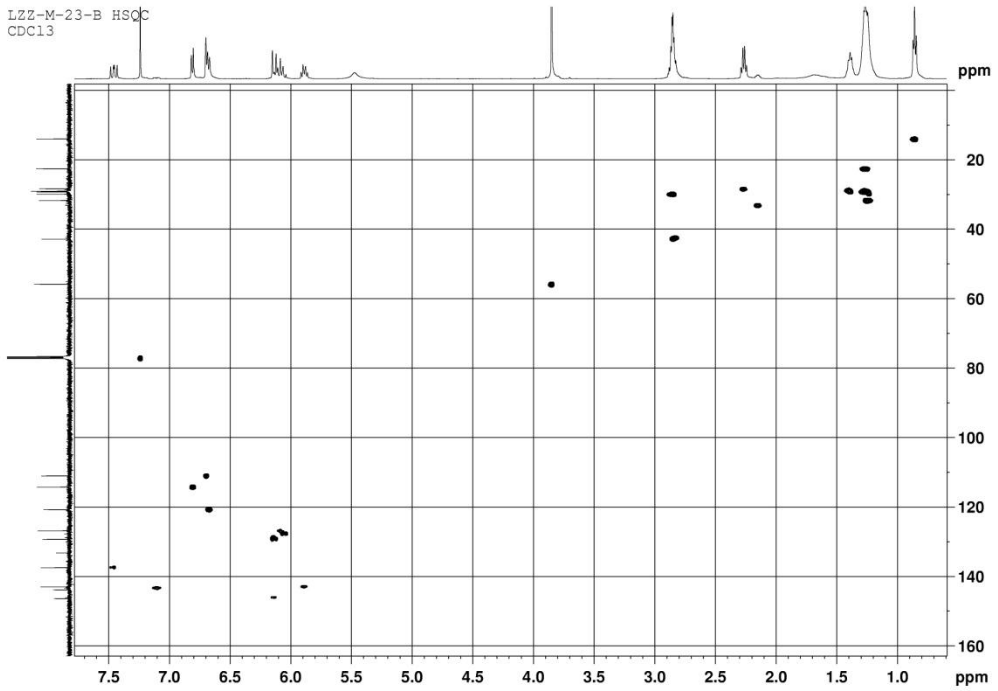

S75. HMBC spectrum of **11**

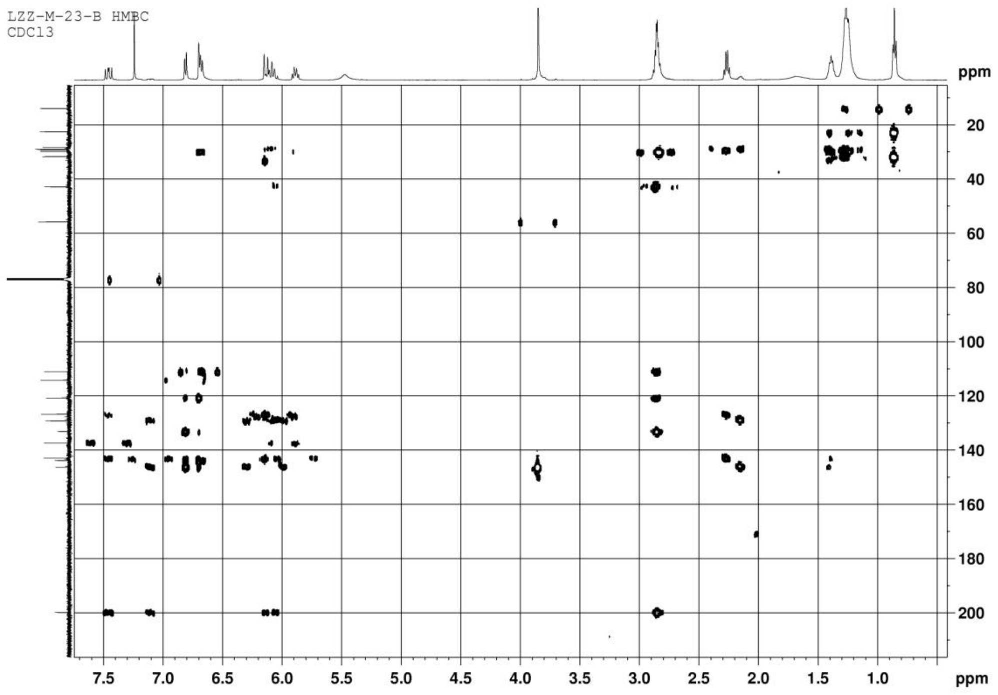

S76. NOESY spectrum of **11**

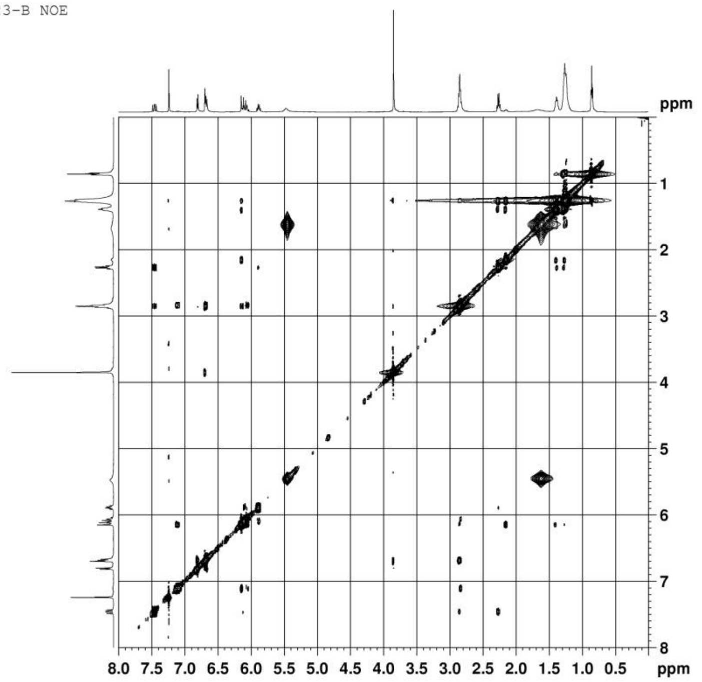

S77. HRESIMS spectrum of **11**

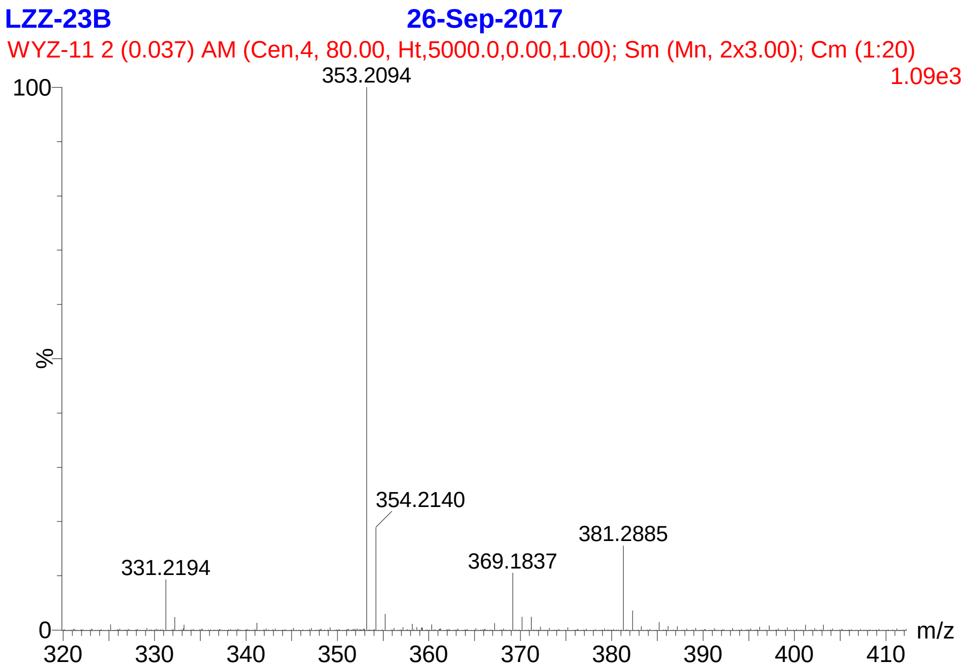

S78. IR spectrum of **11**

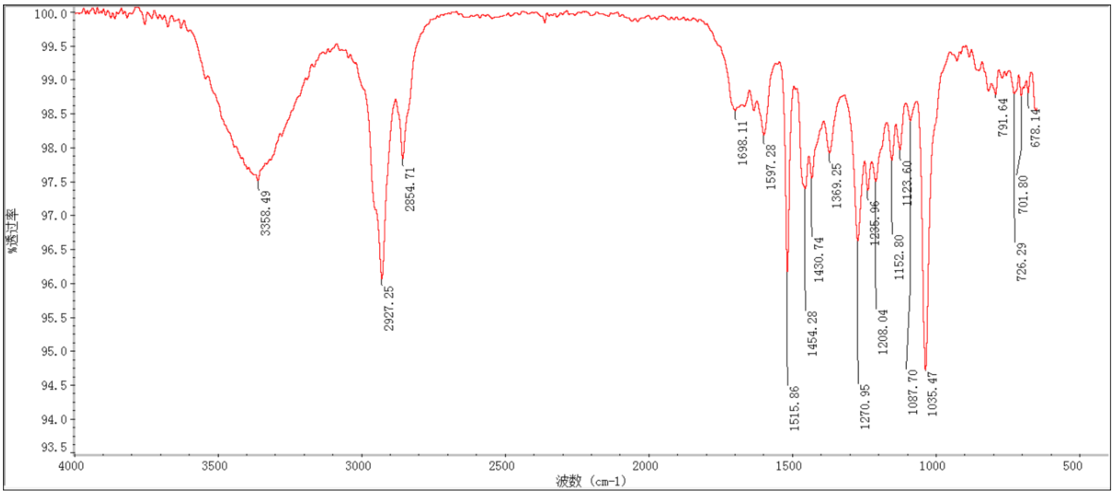

S79. UV spectrum of **11**

Thermo Scientific ~ VISIONpro SOFTWARE V4.41

Operator Name	(None Entered)	Date of Report	2017/11/7
Department	(None Entered)	Time of Report	17:56:25下午
Organization	(None Entered)		
Information	(None Entered)		

Scan Graph

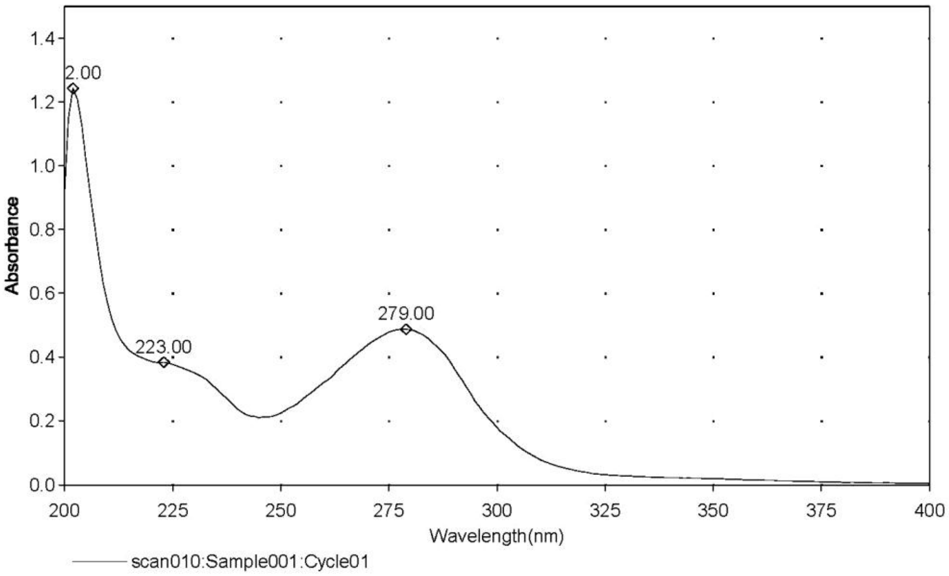

Results Table - scan010,Sample001,Cycle01

nm	A	**Manual Method**
202.00	1.242	Report Values at 3 Wavelengths
223.00	.383	202.00 nm 223.00 nm 279.00 nm
279.00	.487	Sort By Wavelength

Page 1, Scan Graph

S80. ^{1}H NMR spectrum of **12** (500MHz, CDCL$_3$)

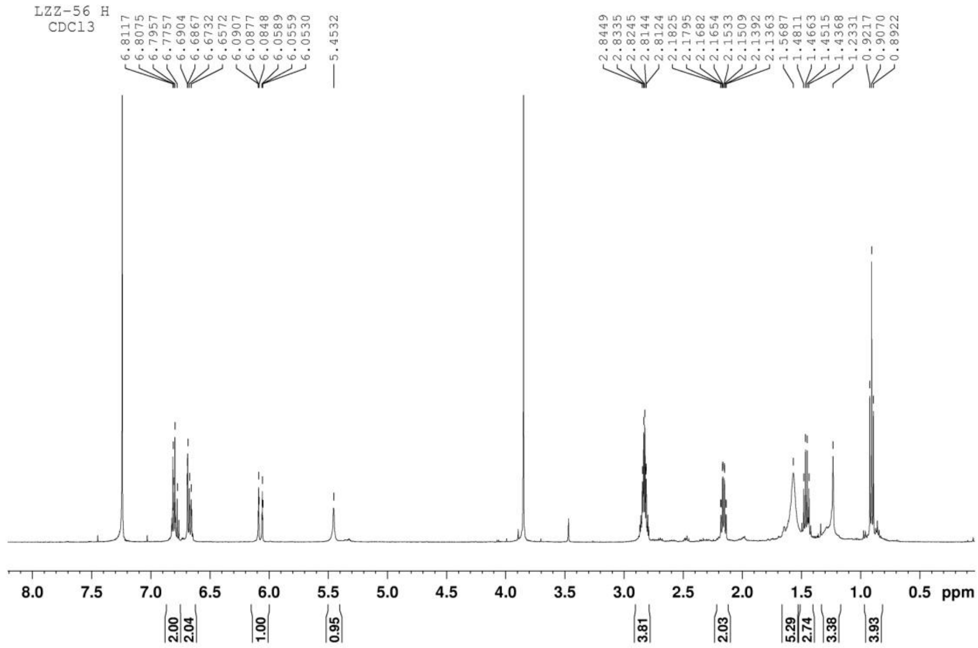

S81. ^{13}C NMR spectrum of **12** (125MHz, $CDCL_3$)

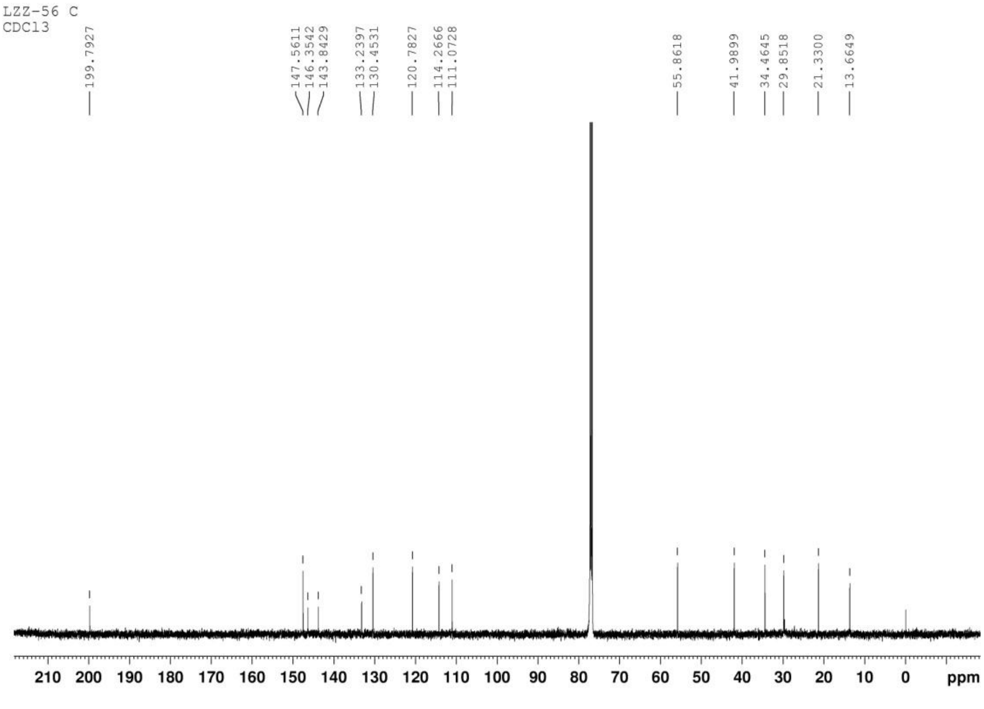

S82. ^{1}H NMR spectrum of **13** (500MHz, $CDCL_3$)

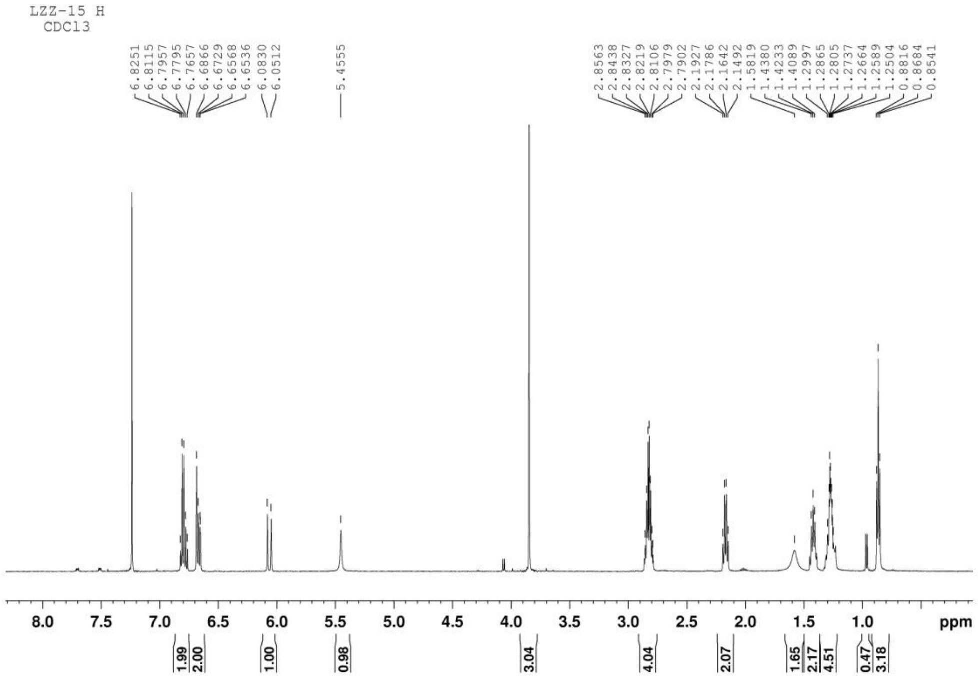

S83. ^{13}C NMR spectrum of **13** (125MHz, $CDCL_3$)

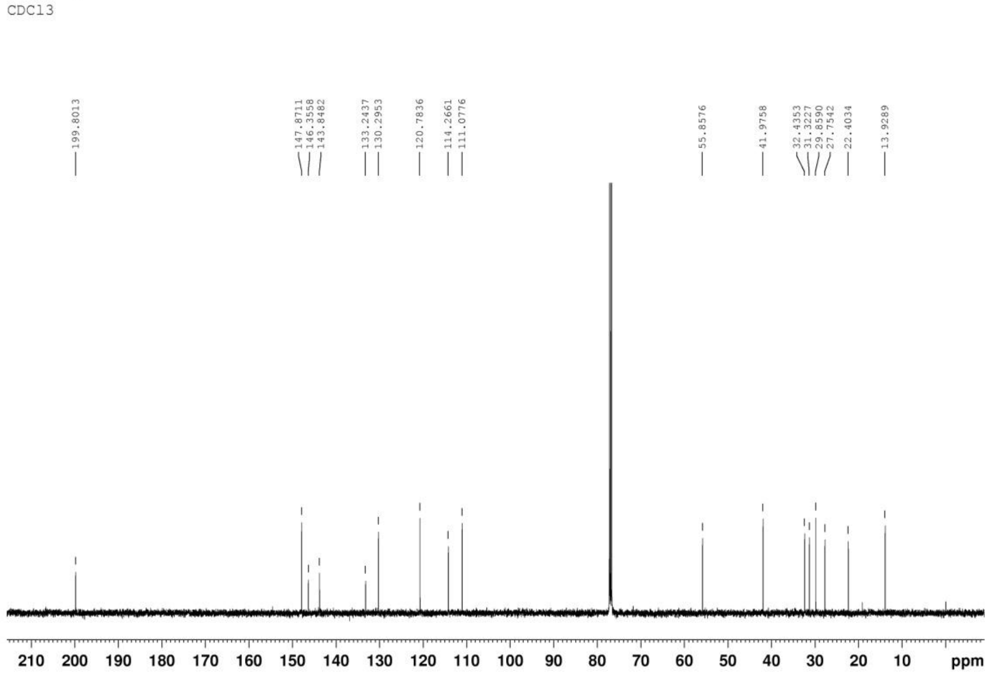

S84. ^{1}H NMR spectrum (500MHz, $CDCL_3$) of **14**

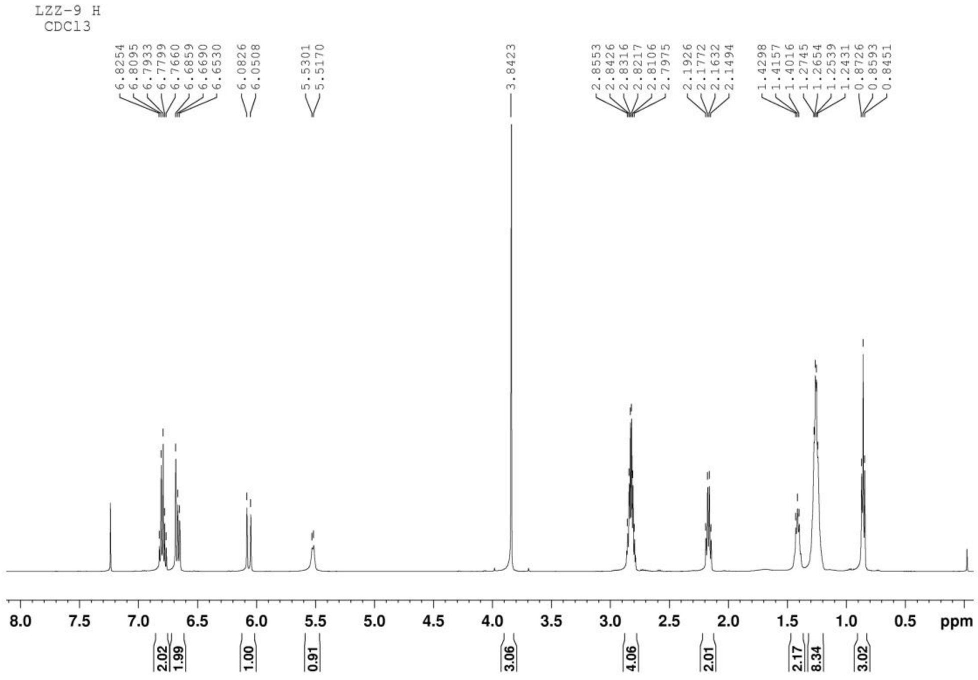

S85. ^{13}C NMR spectrum (125MHz, $CDCL_3$) of **14**

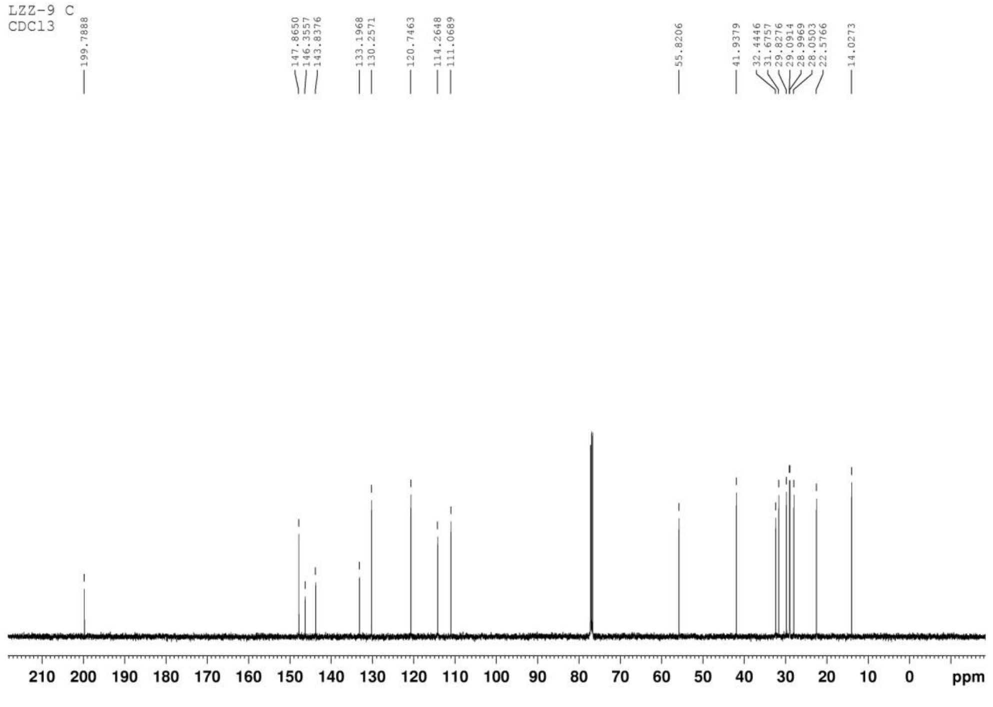

S86. ^{1}H NMR spectrum (500MHz, $CDCL_3$) of **15**

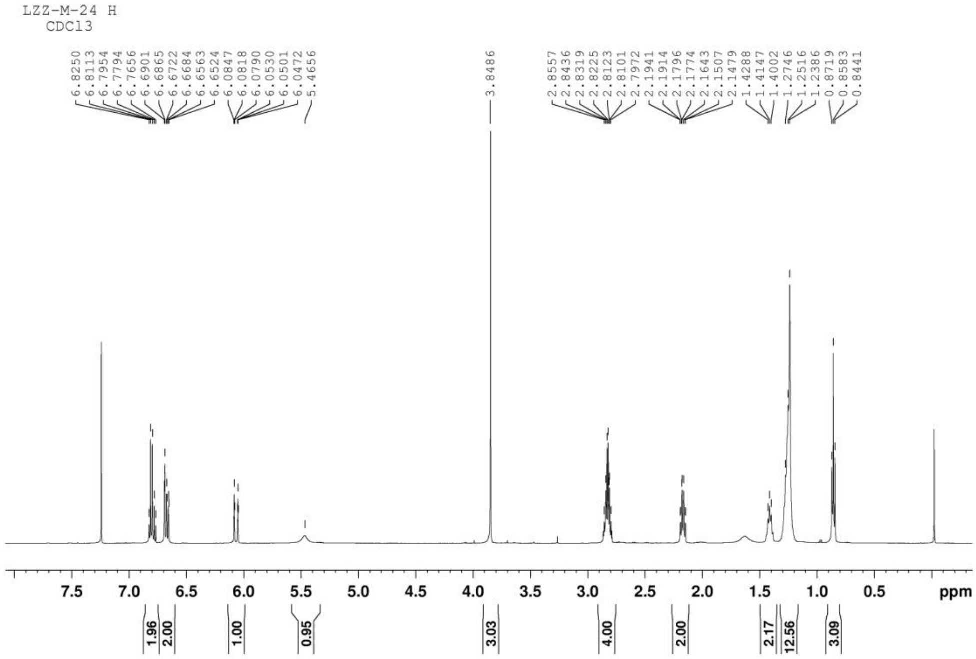

S87. ^{13}C NMR spectrum (125MHz, $CDCL_3$) of **15**

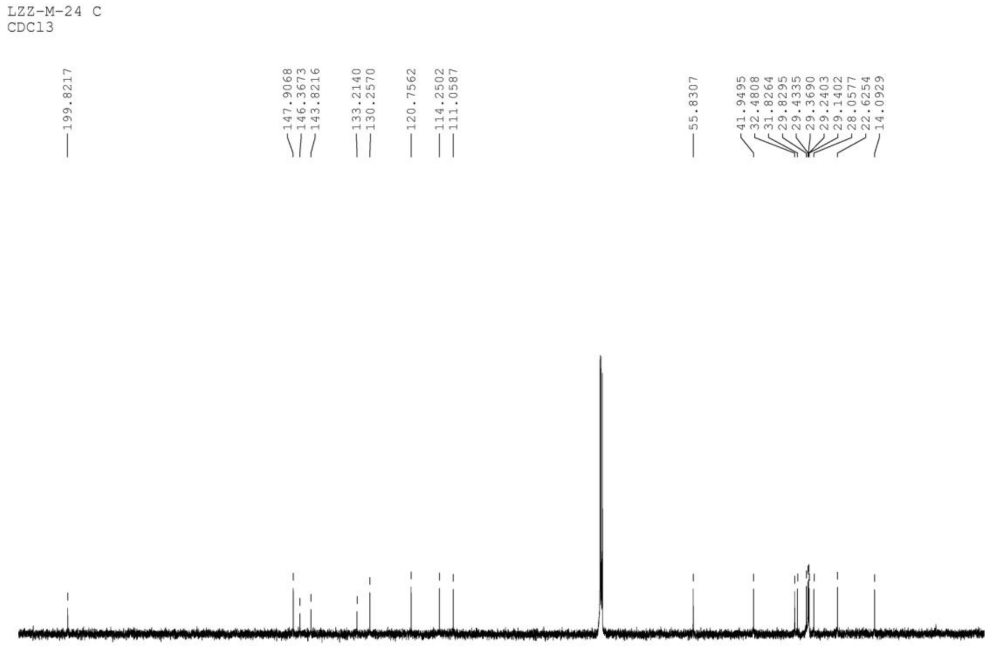

S88. ^{1}H NMR spectrum of 1**6** (500MHz, $CDCL_3$)

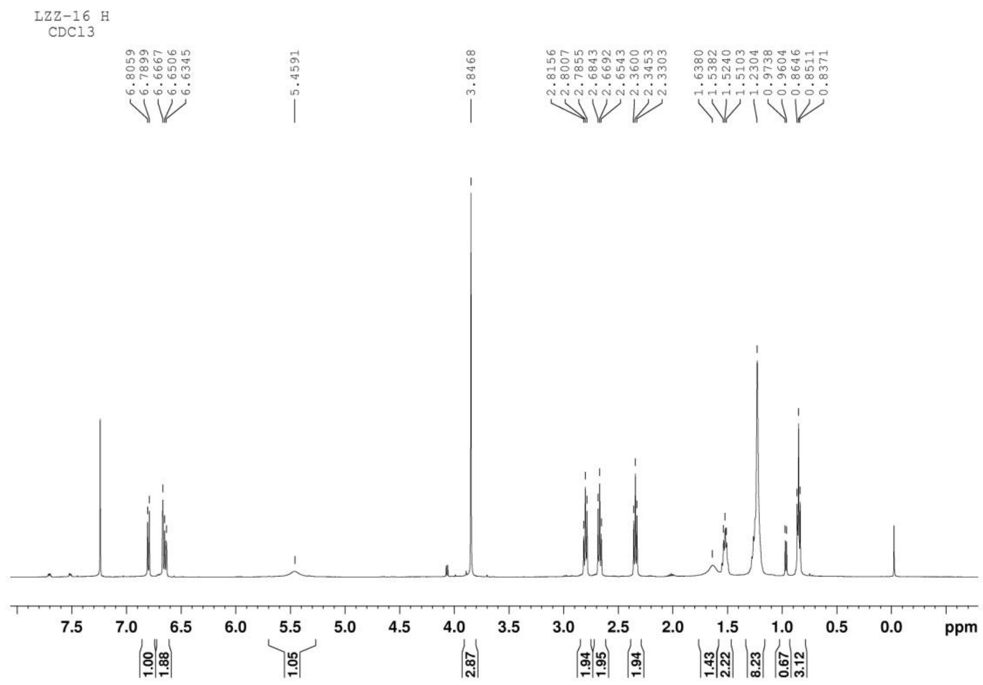

S89. ^{13}C NMR spectrum of 1**6** (125MHz, CDCL$_3$)

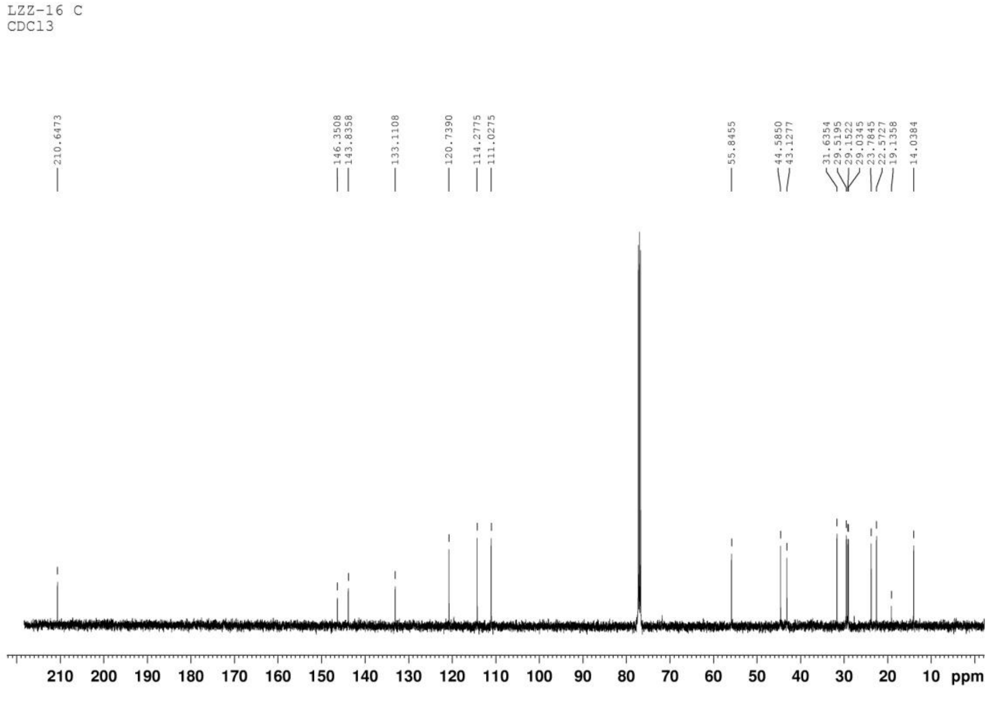

S90. ^{1}H NMR spectrum (500MHz, CDCL$_3$) of **17**

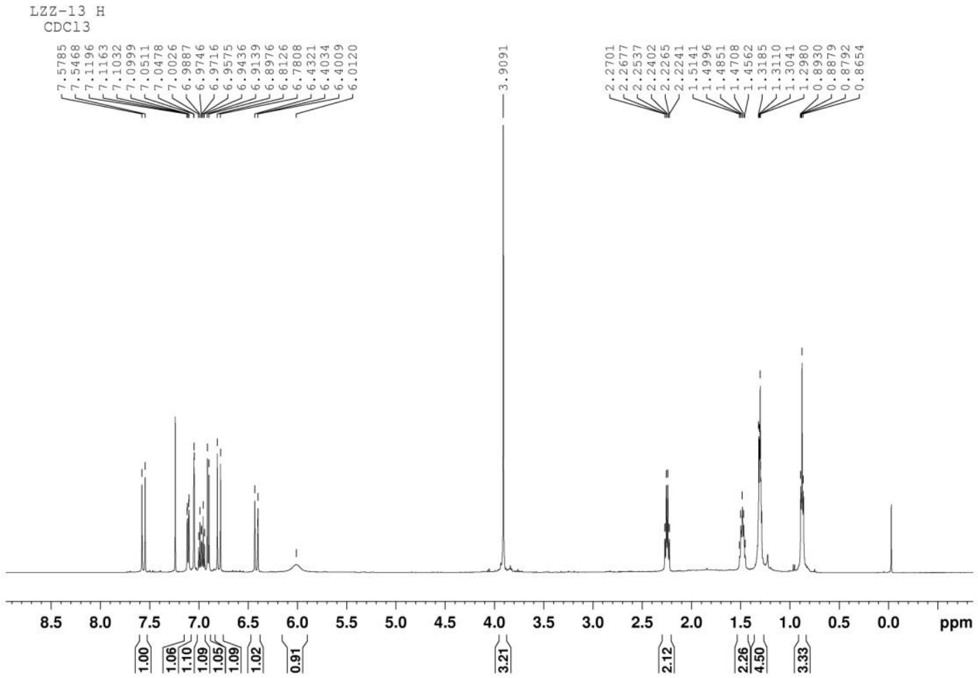

S91. ^{13}C NMR spectrum (125MHz, CDCL$_3$) of **17**

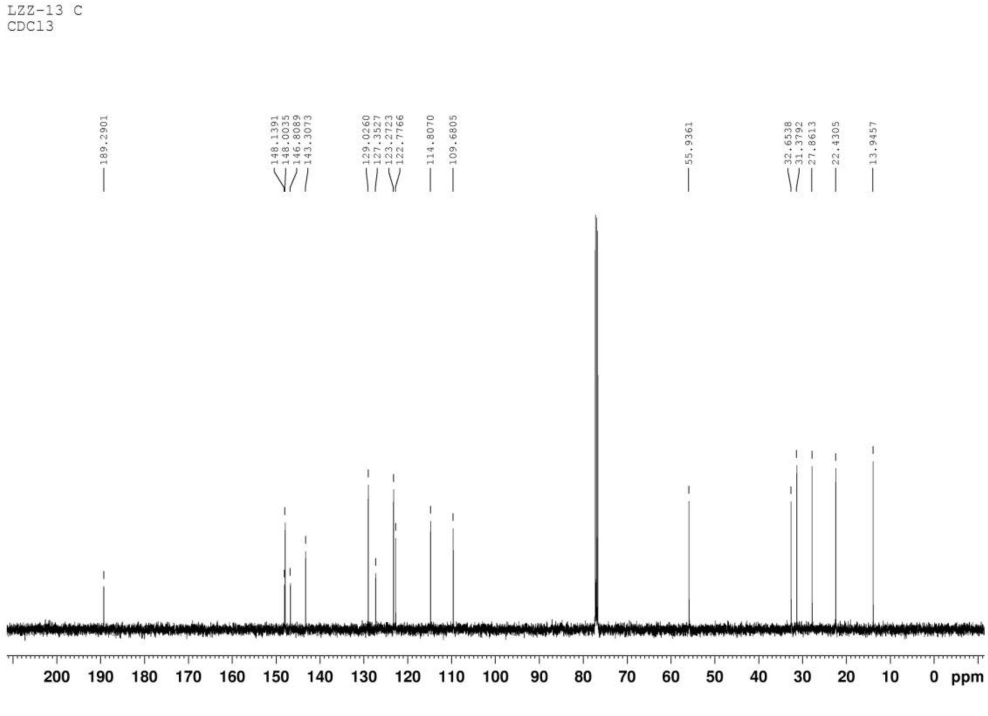

Supplement: Supplementary file 1 [file molecules-23-00315-s001.pdf]
